# Profiling Urinary Sulfate Metabolites With Mass Spectrometry

**DOI:** 10.3389/fmolb.2022.829511

**Published:** 2022-02-23

**Authors:** Christopher C. J. Fitzgerald, Rikard Hedman, Dimanthi R. Uduwela, Bettina Paszerbovics, Adam J. Carroll, Teresa Neeman, Adam Cawley, Lance Brooker, Malcolm D. McLeod

**Affiliations:** ^1^ Research School of Chemistry, Australian National University, Acton, ACT, Australia; ^2^ Australian Racing Forensic Laboratory, Racing NSW, Sydney, NSW, Australia; ^3^ Australian Sports Drug Testing Laboratory, National Measurement Institute, Sydney, NSW, Australia

**Keywords:** sulfation, mass spectrometry, metabolomics, steroid, anti-doping, sulfatase, sulfate ester

## Abstract

The study of urinary phase II sulfate metabolites is central to understanding the role and fate of endogenous and exogenous compounds in biological systems. This study describes a new workflow for the untargeted metabolic profiling of sulfated metabolites in a urine matrix. Analysis was performed using ultra-high-performance liquid chromatography-high resolution tandem mass spectrometry (UHPLC-HRMS/MS) with data dependent acquisition (DDA) coupled to an automated script-based data processing pipeline and differential metabolite level analysis. Sulfates were identified through *k*-means clustering analysis of sulfate ester derived MS/MS fragmentation intensities. The utility of the method was highlighted in two applications. Firstly, the urinary metabolome of a thoroughbred horse was examined before and after administration of the anabolic androgenic steroid (AAS) testosterone propionate. The analysis detected elevated levels of ten sulfated steroid metabolites, three of which were identified and confirmed by comparison with synthesised reference materials. This included 5α-androstane-3β,17α-diol 3-sulfate, a previously unreported equine metabolite of testosterone propionate. Secondly, the hydrolytic activity of four sulfatase enzymes on pooled human urine was examined. This revealed that *Pseudomonas aeruginosa* arylsulfatases (PaS) enzymes possessed higher selectivity for the hydrolysis of sulfated metabolites than the commercially available *Helix pomatia* arylsulfatase (HpS). This novel method provides a rapid tool for the systematic, untargeted metabolic profiling of sulfated metabolites in a urinary matrix.

**GRAPHICAL ABSTRACT:**
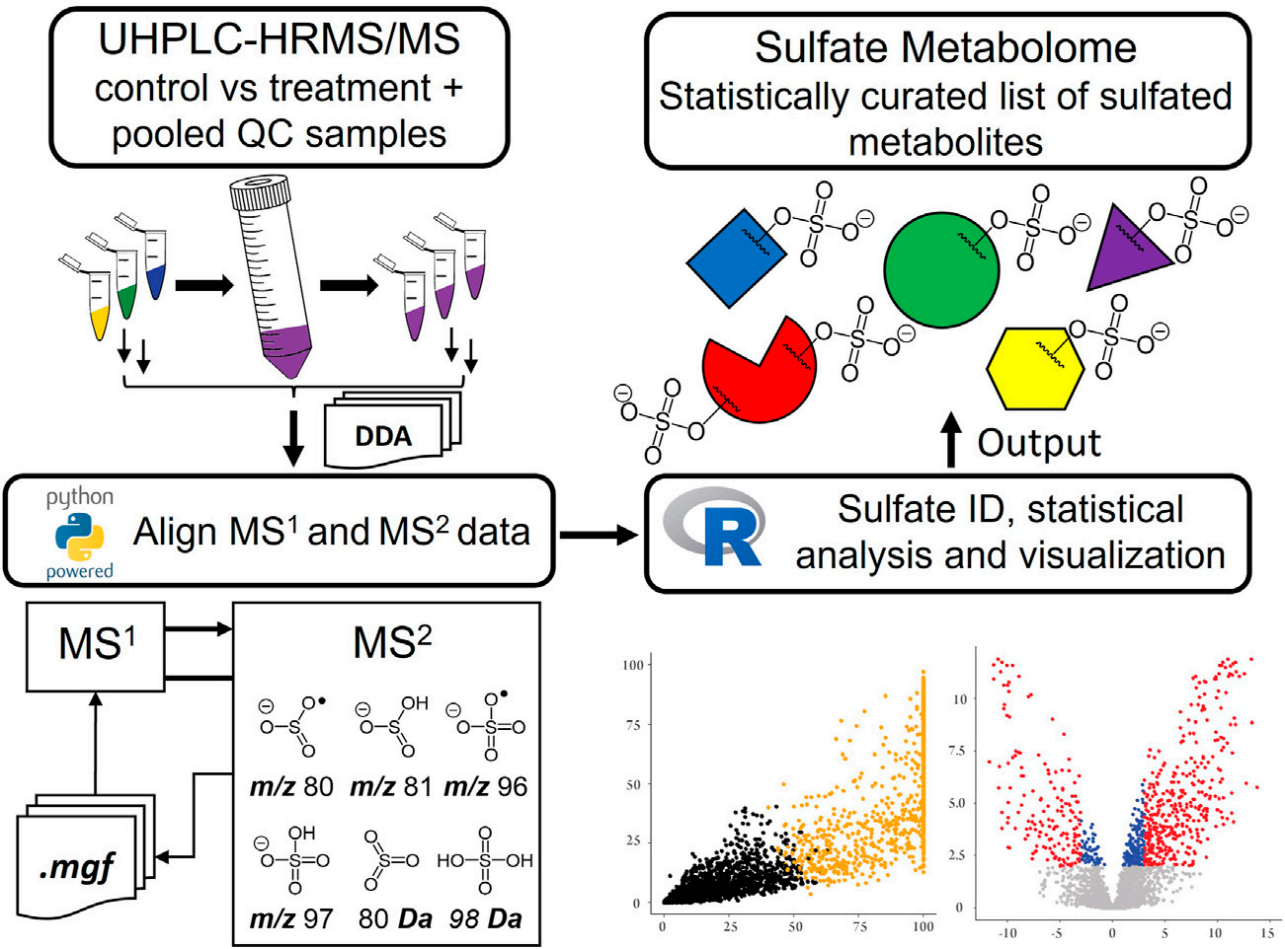


## Introduction

The study of phase II metabolism is essential to understand the biochemical role and fate of endogenous and exogenous compounds and is dominated by the two major classes of conjugates: sulfates and glucuronides (Schänzer, 1996). A compelling example of the importance of phase II conjugation is provided by the field of steroid metabolism, with the conjugates accounting for up to 97% of excreted urinary metabolites (Pranata et al., 2019). Glucuronylation, performed by uridine 5′-diphospho-glucuronosyltransferases (UGTs) has traditionally been the main focus of these investigations, as the major excretory phase II pathway, and due to the ready availability of β-glucuronidase enzymes for selective deconjugation. More recently, the study of sulfate conjugates has gained prominence due to the intriguing interplay between sulfotransferase (SULT) mediated synthesis, sulfatase promoted hydrolysis and a range of transport phenomena (Foster and Mueller, 2018; Uduwela et al., 2018; Günal et al., 2019). The systematic study of the sulfated metabolome in a variety of contexts will be pivotal in revealing the roles of sulfation pathways in biology.

The field of metabolomics initially emerged as a complementary approach to genomics and proteomics (Fiehn et al., 2000; Lafaye et al., 2004). However, metabolic profiling has grown into its own field and serves as a powerful way to identify biomarkers in an unbiased fashion, including with relative quantification (Fiehn et al., 2000; Tolstikov and Fiehn, 2002). Metabolomic methods can be applied to the study of disease states or perturbations to homeostasis, giving unprecedented insight into biological processes (Antignac et al., 2005; Pozo et al., 2018). The study of sulfate conjugated metabolites has been of particular interest in metabolomics and found useful applications in applied sciences such as anti-doping and medical research (Pranata et al., 2019; Mueller et al., 2021). A range of tools are available, including the use of nuclear magnetic resonance (NMR), and various hyphenated chromatographic-tandem mass spectrometry (MS/MS) techniques (Lafaye et al., 2004). Early approaches to examine the sulfate metabolome employed gas chromatography-mass spectrometry (GC-MS) techniques, however, these suffered from various limitations due to the polar character of the conjugates, challenges with reliable enzymatic or chemical cleavage of sulfate esters, and loss of information about conjugation sites and levels (Gomes et al., 2009). This directed research towards liquid chromatography-mass spectrometry (LC-MS) techniques, which permit the direct detection of the intact sulfate conjugates, allowing the sulfate fraction to be specifically targeted during analysis. This type of analysis heavily relies on MS/MS techniques driven by the capacity of sulfate conjugates to readily undergo ionisation during electrospray ionisation (ESI), and display distinctive fragmentation behavior (Thevis et al., 2011; Gómez et al., 2012; Balcells et al., 2017a). Approaches based on tandem mass spectrometry include, multiple reaction monitoring (MRM), precursor ion scanning, or constant ion loss (CIL) scanning, which have all been used to selectively and directly detect sulfate conjugated metabolites (Bowers and Sanaullah, 1996; Bean and Henion, 1997; Hintikka et al., 2008; Gómez et al., 2013; Balcells et al., 2017a; McLeod et al., 2017; Schänzer and Thevis, 2017).

An early example of LC-MS based profiling of sulfate conjugates was in human and rat urine. Here sulfated molecules were identified by monitoring the neutral loss of 80 Da (SO_3_), precursors of *m/z* 80 (^•^SO_3_^−^) and of *m/z* 97 (HSO_4_^−^), using ESI triple-quadrupole MS/MS (Lafaye et al., 2004). These methods were used to identify tens of sulfate metabolites, some of which were then confirmed through synthesis of the corresponding reference materials (Lafaye et al., 2004). A similar study used ultra-high-performance liquid chromatography (UHPLC)/negative ion matrix-assisted laser desorption ionization tandem time-of-flight high resolution mass spectrometry (MALDI-TOF/TOF-MS) to identify 1,129 potential sulfate candidates from the urine of pregnant women with detection based on the neutral loss of 80 Da and/or the formation of the *m/z* 97 product ion. Database matches were investigated for these candidates but none were confirmed against reference materials (Yao et al., 2016). More recently a method for the detection of sulfated metabolites in gut-microbiota using UHPLC-MS in an untargeted fashion was reported (Ballet et al., 2018). In this study sulfated metabolites were identified by comparing features that underwent change after treatment with a purified preparation of the commercially available sulfatase enzyme *Helix pomatia* arylsulfatase (HpS). Structural validation was then achieved through MS/MS fragment identification, by comparison with reference materials or through data base matching. The method was able to confirm the structures of 36 from 206 putative sulfated metabolites identified (Ballet et al., 2018). Although collectively these approaches provide useful tools to study sulfate metabolites, they also have some limitations. In general, these approaches monitor only a selection of the known sulfate ester fragmentation modes and as a result may miss important classes of conjugate or not report information that could aid in characterizing different classes of conjugate. Furthermore, methods such as enzyme hydrolysis, that assess conversions of sulfate esters to their non-sulfated counterparts, only report on species that change and do not necessarily report on those that do not change. Moreover, they may also miss those product species with low ionisation efficiencies. Herein, we report the development of an untargeted metabolomics approach to comprehensively profile the sulfate metabolome in a urinary matrix. It employs ultra-high performance liquid chromatography-tandem high resolution mass spectrometry (UHPLC-HRMS/MS) with data dependent acquisition (DDA) and a novel data analysis pipeline to systematically identify sulfated metabolites by studying fragmentation behavior. The suitability of the method was evaluated in two applications: firstly, as a screening tool to identify potential steroid markers in equine urine post doping with testosterone propionate; and secondly, to monitor the performance of sulfatase enzymes for the hydrolysis of sulfate esters in pooled human urine. This method increases the utility of untargeted metabolomics for sulfate biomarker discovery in a urinary matrix.

## Materials and Methods

### Animal Administration

Animal administration was approved by both Charles Sturt University (Wagga Wagga, NSW, Australia) and Racing NSW Animal Care and Ethics Committees. Testoprop^®^ (testosterone propionate, 250 mg) was administered by intramuscular injection in the neck, opposite to the sampling jugular catheter to a thoroughbred gelding (635 kg, 14-year-old). Urine samples were collected at −72, −48, −24, 0, 2, 4, 6, 8, 12, 24, 36, 48, 72 h and then daily to 28 days after administration. Samples were stored at −20°C at the Racing NSW or the Australian National University (ANU) in sterile Falcon tubes (polypropylene, centrifuge tubes, 50 ml).

### Equine Urine Sample Preparation

The method has been reported previously and adapted according to our work (Waller et al., 2016). To an aliquot of urine (1.1 ml), phosphate buffer (0.55 ml, 100 mM, pH 7.4) was added and the samples were centrifuged (1,100 x *g*, 5 min) to pellet solids. Each supernatant was fortified with a mixture of analytical internal standards nandrolone sulfate (**S1**), cholanediol bis(sulfate) (**S2**), epiandrosterone (^18^O_3_)-sulfate (**S3**) and 5α-androstane-3β,17β-diol 3,(^18^O_3_)17-bis(sulfate) (**S4**) (0.150 ml, final concentration equivalent to 300 ng/ml original urine volume (1.1 ml) per standard). At this stage, the supernatants were split equally into biological samples and pooled quality control (QC) samples (0.818 ml, equivalent to 0.5 ml of original urine volume). The latter were pooled and then redistributed as pooled QC aliquots (0.818 ml). Both supernatant and pooled QC samples (0.818 ml) were then loaded onto a Waters Oasis™ WAX SPE cartridge (3 cc), that was pre-conditioned with methanol (2 ml) and water (2 ml). Samples were washed with NaOH (2 ml, 0.1 M), phosphate buffer (2 ml, 100 mM, pH 7.4), and Milli-Q water (2 ml) before elution with a mixture of ethyl acetate: methanol: diethyl amine (25:25:1 v/v/v, 3 ml) into clean 10 ml glass tubes. Samples were then evaporated to dryness under a reduced pressure at 40°C and stored at -20°C. The dried samples were re-dissolved with acetonitrile: water (20% v/v, 50 µL), filtered using 0.2 µm spin filters and stored at 5°C for analysis.

### Enzyme Hydrolysis in Human Urine Sample Preparation

The human urine samples used in this study were collected with approval by the ANU Human Research Ethics Committee, in accordance with the 2007 National Statement on Ethical Conduct in Human Research (approval number 2013/654). Volunteers gave written informed consent prior to participation; they were all healthy and reported not using steroids within 1 month of supplying a sample. Urine was taken from six subjects (three females and three males ranging between 20–50 years old) and pooled, into phthalate free plastic containers (Nalgene ® bottles, style 2110) and stored at −20°C. The following procedure was based on an established method (Stevenson et al., 2015). Aliquots of pooled human urine (2.1 ml) were pipetted into 15 ml falcon tubes. The samples were either adjusted to a pH of 7.5 ± 0.2 (PaS enzymes and control) or to 4.0 ± 0.2 (HpS enzyme) with the addition of Tris buffer (1.5 ml, 0.2 M) or acetate buffer (1.5 ml, 0.2 M) with mixing, respectively. To their respective tubes, preparations of purified WT-PaS, PVFV-PaS, LEF-PaS, or crude HpS (0.4 ml, PaS preparations 2.0 mg/ml, HpS 5.9 mg/ml) and for the control sample enzyme storage buffer (0.4 ml, 0.1 mM Tris-HCl, pH 7.5 in 50% v/v glycerol) were added. The quantities of enzyme were normalized according to their rates of *para*-nitrophenyl sulfate hydrolysis (Supplementary Table S7) (Stevenson et al., 2015). Hydrolysis reactions were performed in triplicate for a total of 15 samples and controls by overnight incubation at 37°C. Samples were centrifuged (1,100 x *g*, 5 min) and the supernatants were split equally (1.9048 ml each, equivalent to 1 ml of original urine volume) into biological and pooled QC samples. All pooled QC samples were pooled and redistributed into 1.9048 ml pooled QC aliquots. Each supernatant and pooled QC were fortified with a mixture of analytical internal standards nandrolone sulfate (**S1**), cholanediol bis(sulfate) (**S2**), epiandrosterone (^18^O_3_)-sulfate (**S3**) and 5α-androstane-3β,17β-diol 3,(^18^O_3_)17-bis(sulfate) (**S4**) (0.300 ml, final concentration equivalent to 300 ng/ml urine volume (1 ml) per standard). The treatments, control and pooled QC samples were then subjected to SPE as above, with tris-HCl buffer (2 ml, 0.2 M) being used in place of phosphate buffer in the wash phase. Samples were then evaporated to dryness under reduced pressure at 40°C, and the dried samples were stored at −20°C until analysis. For analysis samples were reconstituted in MeOH: water (20% v/v, 100 µL).

### UHPLC-HRMS/MS Analysis

The study was carried out using a Q-Exactive Plus Orbitrap mass spectrometer equipped with a heated electrospray ionisation source (HESI-II) interfaced to an Ulti-Mate 3000 system for chromatographic separation (all from Thermo Fisher, Scoresby, Australia). For equine urine samples: the column used was an Acquity UPLC CSH phenyl-hexyl column (2.1 × 100 mm, i.d., 1.7 µm) fixed to an Acquity UPLC CSH phenyl-hexyl VanGuard Pre-Column (2.1 × 5 mm i.d., 1.7 µm). The UHPLC separation was performed at flow rate 0.4 ml/min, using gradient mixing of two mobile phase components. Solution A: 20 mM ammonium formate in water; and solution B: 20 mM ammonium formate in acetonitrile: water (90% v/v). The gradient was 0–0.5 min (20% B), 0.5–15 min (20–58% B), 15–20.5 min (58–100% B), 20.5–21.5 min (100–20% B), 21.5–30.0 min (20% B). The injection volume was 5 µL and the column oven temperature was 40°C. For human urine samples: the column used was a polar end capped Thermo Accucore aQ C18 column (2.1 × 100 mm, 2.6 µm). The UHPLC separation was performed at a flow rate 0.4 ml/min, using gradient mixing of two mobile phase components; solution A: 5 mM ammonium formate in water; and solution B: 5 mM ammonium formate in methanol: water (99% v/v). The gradient was 0–20 min (1–100% B), 20–25 min (100% B), 25–26 min (100–1% B) and 26–35 min (1% B). The injection volume was 5 µL and the column oven temperature was 40°C. For HRMS analysis: the spray voltage was 2.50 kV, capillary temperature 250°C, S-lens RF level 50, and auxiliary heater temperature 350°C. Mass calibration was performed in negative mode: using propionic acid (*m/z* 73.0295), isobutyric acid (*m/z* 87.0452), heptanoic acid (*m/z* 129.0921), in addition to Pierce ESI negative ion calibration solution. Scan spectrum acquisition with a resolution of 70,000 (Full Width at Half Maximum; FWHM) and scan range of *m/z* 200 to 2000 was used in negative mode. The automatic gain control (AGC) was set to 3 × 10^6^. Data dependent acquisition (DDA) MS/MS spectra (*m/z* 50–500) were collected, between 1 and 20 min, with a resolution of 17,500 (FWHM) on the top 10 precursors in each scan window. The intensity threshold (minimum intensity to initiate a DDA scan) was 8.0 × 10^4^. The apex trigger was set to 2–6 s, and dynamic exclusion was used to exclude already selected ions for the following 3 s. A static exclusion list was also used to exclude precursor ions from DDA that did not emanate from the urine samples but were instead derived from solvents and other method components that were observed in extraction blank samples. This list consisted of the 1,000 most abundant ions detected from two incubated water extraction blank samples, which were taken through the whole sample treatment. For experiments with pooled QC samples, the injection sequence started by running two blanks followed by running at least 10 system suitability samples and two pooled QC samples, followed by the main sequence. For both applications: biological samples were performed in triplicate and analysed in a random order, with pooled QC samples dispersed evenly throughout. In targeted parallel reaction monitoring (PRM) MS/MS experiments for metabolite confirmation, a list of single *m/z* was selected and fragmented over multiple NCE’s, for both reference material and biological samples.

### Data Workflow Pipeline and Processing

Data alignment, LOWESS normalization of total ion chromatograms (TIC), and Principal Component Analysis (PCA) analysis was performed in MS-DIAL by applying tolerances to both retention time (RT) (±3 s) and mass accuracy (Δ*m/z* ± 5 ppm), alongside the recommended settings for DDA-HRMS systems (Tsugawa et al., 2015). Further data analysis was performed either in python or R (Supplementary Section S3). A Python script was used to extract and append MS/MS data for each aligned feature that contained a sulfate-derived fragment. The *k-*means clustering function in R was used to sort metabolic features (Hartigan and Wong, 1979; Team, 2017). Two clusters were chosen as they represented the data appropriately (Supplementary Figures S3, S4) (Kaufman and Rousseeu, 2009). Clustering was performed based on the normalized data of eight unique parameters all derived from the MS/MS spectra. These included the six characteristic sulfate-derived fragments (Table 1). (Attygalle et al., 2001; Yi et al., 2006; Farrell et al., 2011; Balcells et al., 2017b; McLeod et al., 2017; Esquivel et al., 2018) The remaining two parameters were Intensity Ratio (IR): the total sum of sulfate-derived fragments divided by the sum of all fragments, and Maximum Abundance (MA): the normalized relative abundance (%) of the largest sulfate-derived fragment. Data were clustered into non-sulfate and sulfate groupings. High throughput differential metabolite level analysis and data visualization was performed in R, using the *limma* and *ggplot2* packages*,* respectively (Ginestet, 2011; Ritchie et al., 2015; Team, 2017). The output gives a final curated list of metabolic features that were sorted into sulfate and non-sulfate metabolites, with associated statistics [e.g., adjusted *p*-value, log_2_ Fold Change (FC)], UHPLC-HRMS/MS data (RT and *m/z*), and product ion data for any sulfate-derived fragments detected by MS/MS (see Supplementary Table S14 for an example).

**TABLE 1:** Masses used to identify sulfate-derived fragments and neutral lossess (Attygalle et al., 2001; Yi et al., 2006; Farrell et al., 2011; Balcells et al., 2017b; McLeod et al., 2017; Esquivel et al., 2018).

Ion (*m/z*)	Nominal	Accurate
^•^SO_3_^−^	80	79.9573
HSO_3_^−^	81	80.9652
^•^SO_4_^−^	96	95.9523
HSO_4_^−^	97	96.9601
Neutral loss (Da)		
SO_3_	80	79.9568
H_2_SO_4_	98	97.9674

### Synthesis of Anabolic Androgenic Steroid Sulfate Metabolite Reference Materials

Briefly, the synthesis of sulfated reference materials was performed as follows and was adapted from previous work (Waller and McLeod, 2014): SO_3_•py (30 mg, 188 mmol) was added to a solution of free steroid (5 mg) in dimethylformamide (0.5 ml) the resulting reaction mixture was capped and stirred at room temperature for 3 h. The reaction was then quenched with water (10 ml) and loaded onto a pre-conditioned Waters Oasis C18 SPE cartridge (3 cc). The reaction mixture was washed with aqueous ammonia solution (2 ml, 5% v/v) followed by water (2 ml). Steroid sulfates were then eluted in aqueous ammonia methanol solution (3 ml, 5% v/v). The methanolic ammonia fraction was then concentrated in vacuo to yield the desired steroid sulfate as an ammonium salt.

## Results and Discussion

### Application 1: The effects of Testosterone Propionate Doping on the Urinary Sulfate Metabolome in the Equine

Anabolic androgenic steroids (AAS) and their metabolites are routinely detected in anti-doping urinary analysis (Schänzer and Thevis, 2017). Intact phase II AAS metabolites such as sulfate esters play an important role in anti-doping analysis as long-term biomarkers for both exogenous and endogenous steroids (Schänzer et al., 2013; Piper et al., 2016; Kiousi et al., 2021). Further, sulfation of metabolites is reported to predominate in horses when compared to humans (Scarth et al., 2011). In this application we use the untargeted profiling workflow to assess change in the sulfate urinary metabolome after the intramuscular administration of the AAS testosterone propionate in a thoroughbred gelding (horse 1). This study included a comparison of pre (−24 h) and post (+12 h) administration urine samples, performed in triplicate. These samples were also compared against samples from a non-drug treated gelding (horse 2) at three time points, to assess natural variability over time. Samples were extracted using weak anion exchange (WAX) solid phase extraction (SPE) to isolate the sulfated fraction from the urine.

#### Data Acquisition, Batch Analysis and Alignment

Metabolomic data was acquired using UHPLC-HRMS/MS with data dependent acquisition. The normalized collision energy (NCE) for MS/MS analysis was optimized by studying fragmentation patterns of 20 steroid monosulfate and bis(sulfate) reference materials (Supplementary Table S1). An NCE of 60 eV was chosen for the DDA experiments as all sulfate-derived fragments could be observed with high relative abundance (Table 1). Following data acquisition, detected features were aligned using suitable tolerances (RT ± 3 s and Δ*m/z* ± 5 ppm) in MS-DIAL. Normalization was performed by the use of locally weighted scatterplot smoothing (LOWESS) program (Sangster et al., 2006; Godzien et al., 2015; Broadhurst et al., 2018; Considine et al., 2018; Dudzik et al., 2018). This gave an initial list of 6,230 total detected features. Following this, features without MS/MS spectra, an S/N ratio below three and peaks that appeared in less than two samples, were excluded giving a final curated list of 3467 features. Data was assessed through PCA with good quality data indicated by the tight clustering of replicate samples and pooled QC samples (Supplementary Figure S1) (Broadhurst et al., 2018).

To allow identification of sulfated metabolites, MS/MS spectra of LC/MS features from pooled QC samples were exported to Mascot Generic Format files (.MGF) and analyzed with a custom Python script (Supplementary Section S7) designed to search for accurate mass evidence of known sulfate-associated ions and neutral losses from the precursor ion. During this process two new parameters termed intensity ratio (IR) and maximum abundance (MA) were calculated for each detected feature. These parameters were simple ratios generated from the sulfate-derived fragment ions observed in MS/MS spectra. The IR was the ratio of sulfate-derived fragment ions as a percentage of all product ions in the MS/MS spectrum. The MA was the relative abundance of the sulfate reporter ion with the highest relative abundance. As sulfate metabolites typically display MS/MS spectra dominated by characteristic product ions or neutral losses these ratios were used to help identify sulfate metabolites. These data on sulfate-derived fragments were mapped onto the alignment results from MS-DIAL on the basis of retention time and accurate precursor *m/z* (closest retention time match within tight *m/z* and RT error tolerance limits) using a second custom Python script. (Supplementary Section S7).

#### Identification of Sulfated Metabolites

**FIGURE 1:**
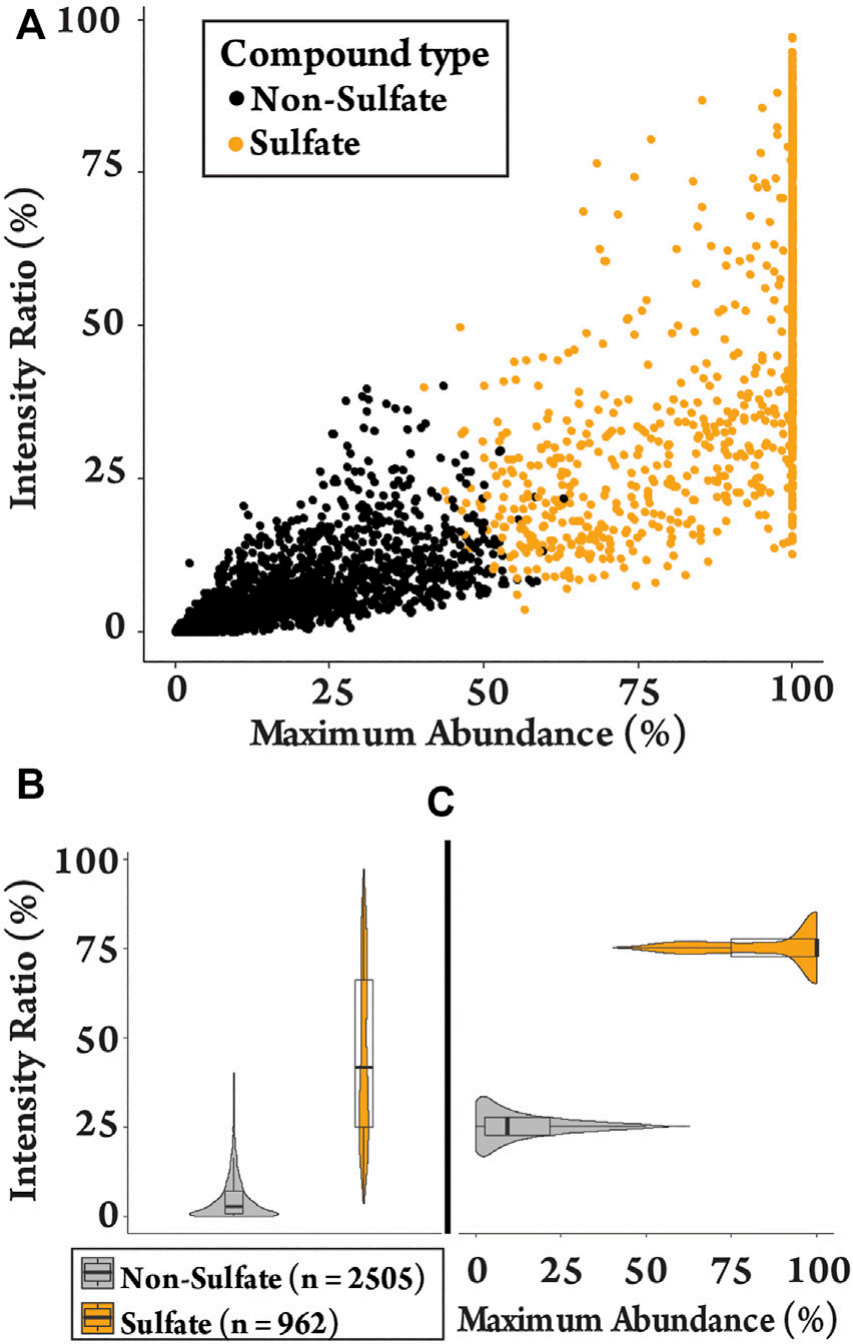
Plots of the detected features (*n* = 3467) in equine urine after administration of testosterone propionate. **(A)** Clustering plot contrasting MA against IR of sulfated and non-sulfated features. **(B)** The spread of IR values for sulfated and non-sulfated features. **(C)** The spread of MA values for sulfated and non-sulfated features.

*k-*means clustering was used to differentiate between sulfated and non-sulfated features in the samples in an unbiased manner. This aims to partition data into groups based on the minimization of the sum of squares to their assigned cluster center. Grouping was based on the six sulfate-derived fragments (Table 1) together with the ratios MA and IR, and were grouped to the nearest center (mean) across the eight parameters, clustered into two groups (Supplementary Figure S3) (Hartigan and Wong, 1979). This separated the 3467 features into 2,505 non-sulfates and 962 sulfates (28%). The clustering of features is visualised with a plot of MA vs. IR (Figure 1A), with a full summary of clustering provided in the supplementary information (Supplementary Table S2). As expected, sulfated features tended to have a larger MA value (i.e., a sulfate-derived fragment is a major peak) and a larger IR value (i.e., sulfate-derived fragments make up a relatively large proportion of total product ions). The distributions of these ratios are visualised as violin plots (Figures 1B,C). They show that the IR of sulfated molecules were spread with a median IR of 42% (Figure 1B, mean = 46%, range = 4–97%) contrasted with the non-sulfated features with a median IR of 3% (mean = 5%, range = 0–40%). The median MA of 99% (Figure 1C, mean = 88%, range = 40–100%) also contrasted to the non-sulfated features with a median MA of 9% (mean = 5%, range = 0–63%). This clustering of sulfated and non-sulfated features was later used to prioritizatise sulfated metabolites for further investigation.

#### Speciation of Sulfates

**FIGURE 2:**
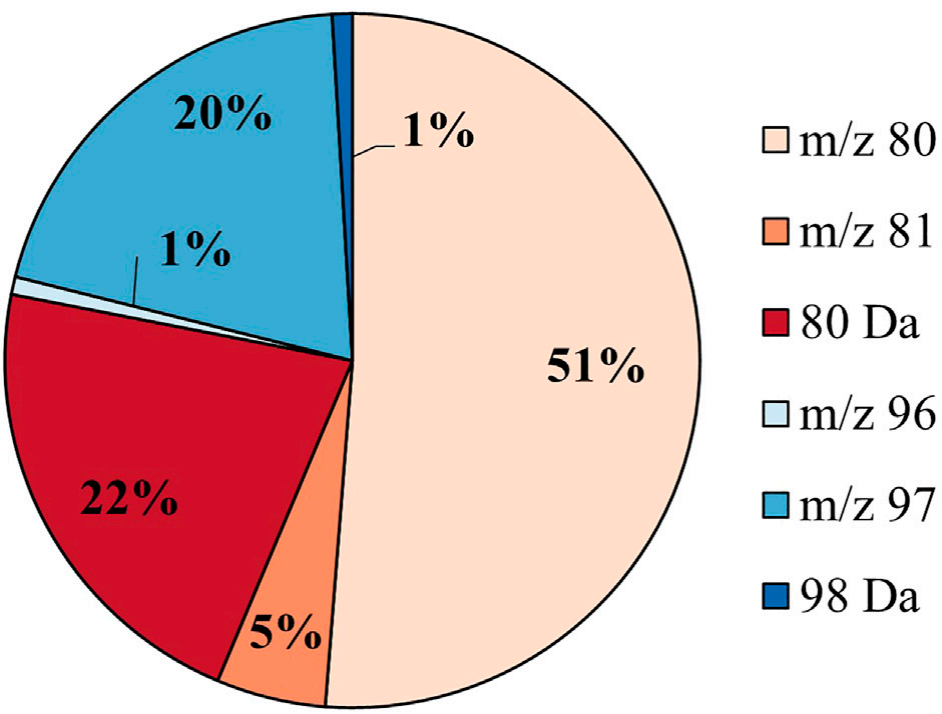
Sulfate speciation for all putative sulfates (*n* = 962) in equine urine. The sulfates are categorized by their most abundant sulfate-derived fragment as defined in Table 1.

The putative sulfated metabolites can be differentiated further based on the observed major sulfate-derived transition (Table1). In the equine urine samples (total sulfates, *n* = 962), the dominant sulfate-derived transition was the ^•^SO_3_^−^ ion (*m/z* 80, *n* = 493, 51%), with the HSO_4_^−^ ion (*m/z* 97, *n* = 194, 20%), and the neutral loss of SO_3_ (80 Da, *n* = 209, 22%) also prominent. Other sulfate-derived fragments including the HSO_3_^−^ (*m/z* 81, *n* = 49, 5%), ^•^SO_4_^−^ (*m/z* 96, *n* = 8, 1%) ions, and H_2_SO_4_ neutral loss (98 Da, *n* = 9, 1%) were observed at a lower frequency (Figure 2). The sulfate-derived ions ^•^SO_3_^−^, HSO_3_^−^ and neutral loss SO_3_ are typically associated with phenolic and other unsaturated sulfate metabolites, while the ion HSO_4_^−^ is associated with saturated sulfates (McLeod et al., 2017). The majority of sulfate metabolites (78%) showed two or more sulfate derived fragments on applying a relative abundance threshold of 5% (data not shown).

#### High Throughput Differential Metabolite Level Analysis

**FIGURE 3:**
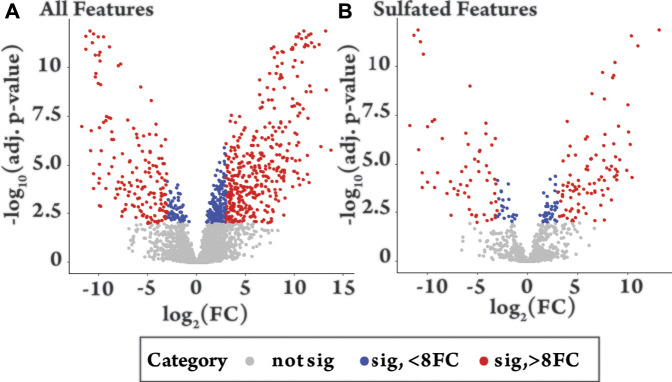
Volcano plots of −log_10_ of the adjusted (adj.) *p*-value against log_2_ fold change (FC) comparing equine urine samples post-administration of testosterone propionate (+12 h) to pre-administration (−24 h). Colour groupings. Grey = not significant (adjusted *p*-value > 0.01), Blue = significant (*p*-value < 0.01) with fold change 0–8, Red = significant (adjusted *p*-value < 0.01) with a fold change >8. **(A)** “All features”, shows the differential metabolite level analysis over all detected features in the plus 12 h sample [*n* = 3467, grey *n* = 2662 (77%), blue *n* = 235 (7%), red *n* = 570 (16%)]. **(B)** “Sulfated Features”, shows differential metabolite level analysis over all sulfated features in the plus 12 h sample [*n* = 962, grey *n* = 747 (77%), blue *n* = 61 (6%), red *n* = 154 (16%)].

To identify features that change significantly following drug administration, a high throughput differential analysis, commonly used to assess gene expression, was applied using the packages *Limma* and *Glimma* in R, from Bioconductor (Supplementary Section S7) (Ritchie et al., 2015; Su et al., 2017; Team, 2017). The results of the analysis comparing the pre- (−24 h) and post drug-administration samples (+12 h) are shown as volcano plots for all features (Figure 3A) and sulfated features (Figure 3B), with the results summarized in Supplementary Table S3. Similar patterns were observed for all features and sulfated features in terms of significant change (red and blue, Figure 3) and positive and negative fold change. Of the 962 sulfated features, 215 had significantly changed (adjusted *p*-value < 0.01) (red and blue, Figure 3B) representing 6% of the total detected features in equine urine after doping. Of these, a larger proportion were upregulated, specifically 136 were upregulated and 79 were downregulated, as indicated by the direction of their fold change. The analysis also showed relatively large inter-horse variation in detected features, which is likely due to individual differences in metabolism between the two horses (Supplementary Figure S8). Comparisons also revealed limited intra-horse variation over time (horse 2, Supplementary Figure S9), but a relatively large intra-horse variation pre- and post-drug administration (Supplementary Figure S8), a pattern of change that could be indicative of increased metabolic activity following administration of the AAS testosterone propionate.

#### Discovery of a Novel Steroid Sulfate Metabolite in Equine Urine

The 215 LC/MS features found to display significant changes in relative signal intensity following testosterone propionate administration could represent direct metabolites of testosterone propionate or other metabolites modulated indirectly in response to testosterone propionate administration. However, this study specifically sought to identify sulfated metabolites derived from the direct metabolism of testosterone propionate. A list of possible metabolites was generated by applying combinations of up to three phase I metabolic transformations, including hydroxylation, bond oxidation and reduction to testosterone, followed by sulfation (Supplementary Table S4). Steroid sulfates in the 215 molecules matching the accurate mass (*m/z* ± 5 ppm) against the generated list of predicted metabolites were identified, using R. Where possible these putative metabolites were confirmed against synthetically derived reference materials by comparison of UHPLC retention time and MS/MS behaviour (Aru et al., 2020).

From this search 30 possible steroid sulfates were identified with theoretical accurate mass matching proposed metabolic transformations (Supplementary Table S4). Of these molecules, 10 were found to be elevated in the range of 16–1024-fold after testosterone propionate administration (+24 h) and by comparison to the control (horse 2), Supplementary Table S5. The identities of testosterone sulfate (**1**) (*m/z* 367.1583), epiandrosterone sulfate (**2**) (*m/z* 369.1739), and 5α-androstane-3β,17α-diol 3-sulfate (**3**) (*m/z* 371.1895) were established after comparison to synthesised reference materials (Table 2, for synthesis of reference materials see Supplementary Section S4). Confirmation was performed according to criteria set out by the Association of Official Racing Chemists (AORC) for UHPLC retention times and MS/MS transitions (Supplementary Table S6) (Association of Offical Racing Chemist, 2020). Scan MS data showed the expected isotope signatures. Of the three confirmed structures, the first two are known biomarkers of testosterone metabolism in equine and human systems (Piper et al., 2017; Esquivel et al., 2019). The final metabolite 5α-androstane-3β,17α-diol 3-sulfate (**3**) has not been described in literature or in online data bases such as ChEBI (Hastings et al., 2016). The upregulated nature of these steroid sulfates also support the general idea of perturbation caused by doping with testosterone propionate (Pozo et al., 2010; Scarth et al., 2011; Vimercati et al., 2017). Future directions for this work could aim at identifying the remaining seven putative steroid sulfate metabolites against reference materials. Longitudinal or population studies would also be required to assess the importance of these markers in doping with testosterone propionate.

**TABLE 2:** Structures of steroid sulfate metabolites and associated high throughput differential level analysis data from the untargeted profiling (log_2_ fold change and adjusted *p*-value). Identified structures were confirmed against synthesized reference materials according to AORC retention time and MS/MS criteria, see Supplementary Table S6.

*m/z*	RT (min)	Log_2_FC	Adjusted *p*-value	Structure	Identity
367.1583	8.77	10	2E-07	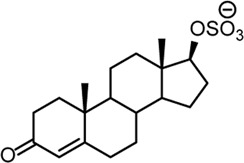	testosterone sulfate (**1**)
369.1739	7.69	9	6E-06	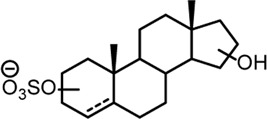	unidentified
369.1739	10.16	6	5E-06	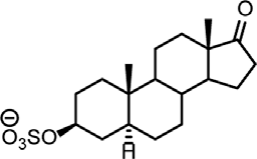	epiandrosterone sulfate (**2**)
371.1895	10.53	8	2E-05	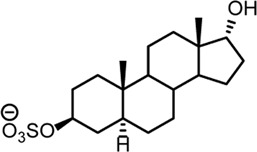	5α-androstane-3β-17α-diol-3-sulfate (**3**)
383.1537	6.37	7	1E-03	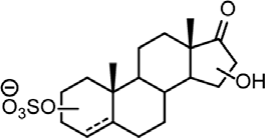	unidentified
385.1690	6.74	4	7E-08	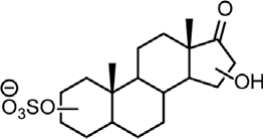	unidentified
387.1845	7.59	10	2E-05	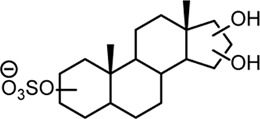	unidentified
387.1846	7.39	8	8E-03		unidentified
387.1846	4.56	9	5E-05		unidentified
387.1849	4.48	9	2E-05		unidentified

### Application 2: Profiling in Sulfatase Treated Human Urine

Hydrolysis is frequently used prior to the analysis of conjugated steroids. In GC-MS analysis, sulfate conjugates are typically hydrolysed to the free steroid and derivatised in analytical workflows, to improve thermal stability and volatility. Hydrolysis is also commonly used in both GC-MS and LC-MS approaches to aid confirmation of metabolites against the more readily available unconjugated steroid reference materials. For sulfated conjugates, hydrolysis is often performed using the commercially available *Helix pomatia* aryl sulfatase (HpS)*.* However, without extensive purification (Ballet et al., 2018), these crude enzyme preparations are known to contain additional enzyme activities such as glucuronidase, oxidase, and reductase, making it unsuitable for many applications (Gomes et al., 2009). Alternatively, chemical solvolysis can be used as a means of deconjugation, however, this can lead to analyte degradation and increased matrix interference (Gomes et al., 2009). Recently, a new recombinantly expressed and purified arylsulfatase from *Pseudomonas aeruginosa* has been investigated for the selective hydrolysis of sulfatase esters (Stevenson et al., 2015). Directed evolution was employed to improve the catalytic efficiency of testosterone sulfate hydrolysis, with improvements in substrate scope and thermostability relative to the wild-type (WT-PaS) enzyme also observed (Uduwela et al., 2018). This application sought to compare the performance of a commercially available HpS preparation to three PaS preparations for hydrolysis of urinary sulfates. Two improved mutants PVFV-PaS and LEF-PaS along with WT-PaS were selected for this evaluation, representing different points along the evolutionary pathway (Uduwela et al., 2018).

#### Profiling the Hydrolytic Activity of Sulfatases in Pooled Human Urine

The workflow described above was used to assess the change in the sulfate metabolome in pooled human urine after incubation with each enzyme preparation. For the hydrolysis study, aliquots of pooled human urine from six healthy people (three females and three males ranging between 20–50 years old) were treated with each enzyme preparation or a control in triplicate and incubated overnight. Enzyme activity was normalized for the hydrolysis of the common *p*-nitrophenyl sulfate prior to the experiment using recommended pH ranges (Supplementary Table S7) (Stevenson et al., 2015). Following this, samples were extracted and then subjected to the described workflow.

Implementing the analytical workflow resulted in 5,774 features with associated MS/MS data from an initial 15,608 detected features in the pooled human urine. The PCA analysis showed tight grouping of all PaS enzyme mutants, distinct from the control, HpS and pooled QC samples (Supplementary Figure S2) (Broadhurst et al., 2018). *k-means* clustering resulted in a total of 1,430 putative sulfates (25%) being identified from the 5,774 features (Supplementary Figure S5). In this, sulfate-derived fragment speciation was dominated by the ions *m/z* 80 (*n* = 630, 44%) and *m/z* 97 (*n* = 550, 38%), with other minor species also observed including neutral loss 80 Da (*n* = 194, 14%), *m/z* 81 (*n* = 52, 4%), neutral loss 98 Da (*n* = 4, 0.3%) and *m/z* 96 (*n* = 8, 1%) (Supplementary Figure S6). In contrast to the equine urine samples this showed (Supplementary Figure S10) a higher proportion of saturated sulfates (*m/z* 97) than unsaturated sulfates (*m/z* 80, *m/z* 81 and 80 Da). The majority of sulfate metabolites (64%) showed two or more sulfate derived fragments on applying a relative abundance threshold of 5% (data not shown).

**FIGURE 4:**
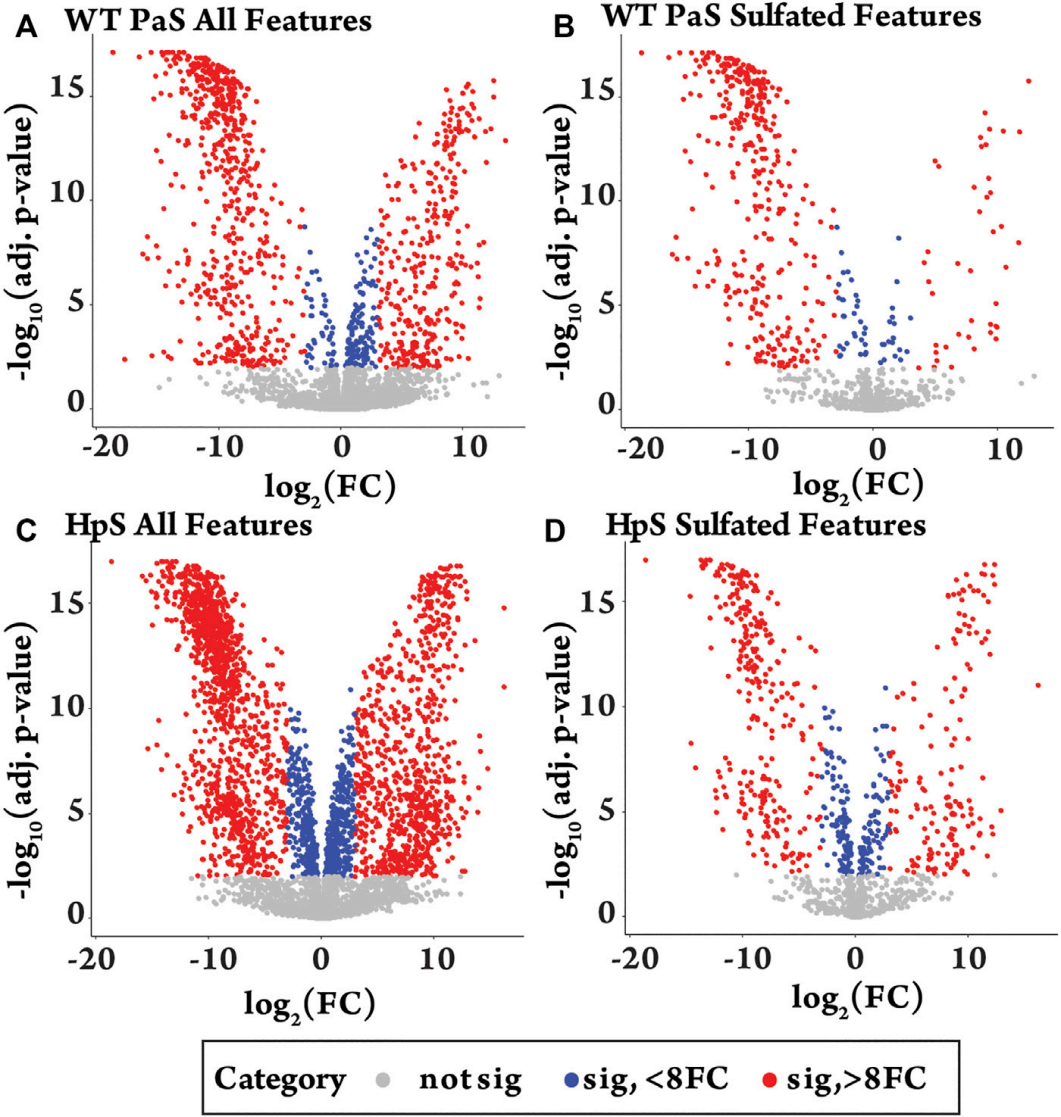
Differential metabolite level analysis for enzyme hydrolysis of pooled human urine. **(A)** All detected features (*n* = 5774) in the WT-PaS treated urine relative to a control sample. **(B)** Sulfate features (*n* = 1430) in WT-PaS treated sample. **(C)** All detected features (*n* = 5774) in the HpS treated urine relative to a control sample. **(D)** Sulfate features (*n* = 1430) in HpS treated sample.

**FIGURE 5:**
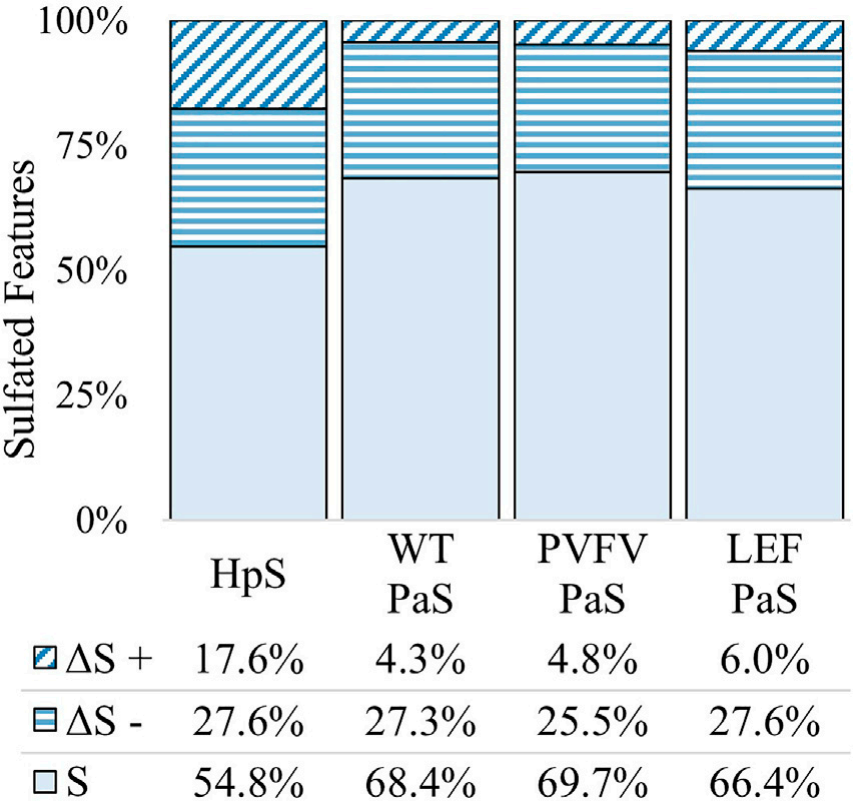
Total change in sulfated features for each sulfatase enzyme treatment of pooled human urine. Unchanged sulfated features are represented by “S” where the adjusted *p*-value > 0.01. Changed sulfated features are represented by “ΔS”, with an adjusted *p*-value < 0.01. The direction of fold change is indicated by “−” or “+” signs. Values reported as a proportion (%) of total sulfated features (*n* = 1430).

**FIGURE 6:**
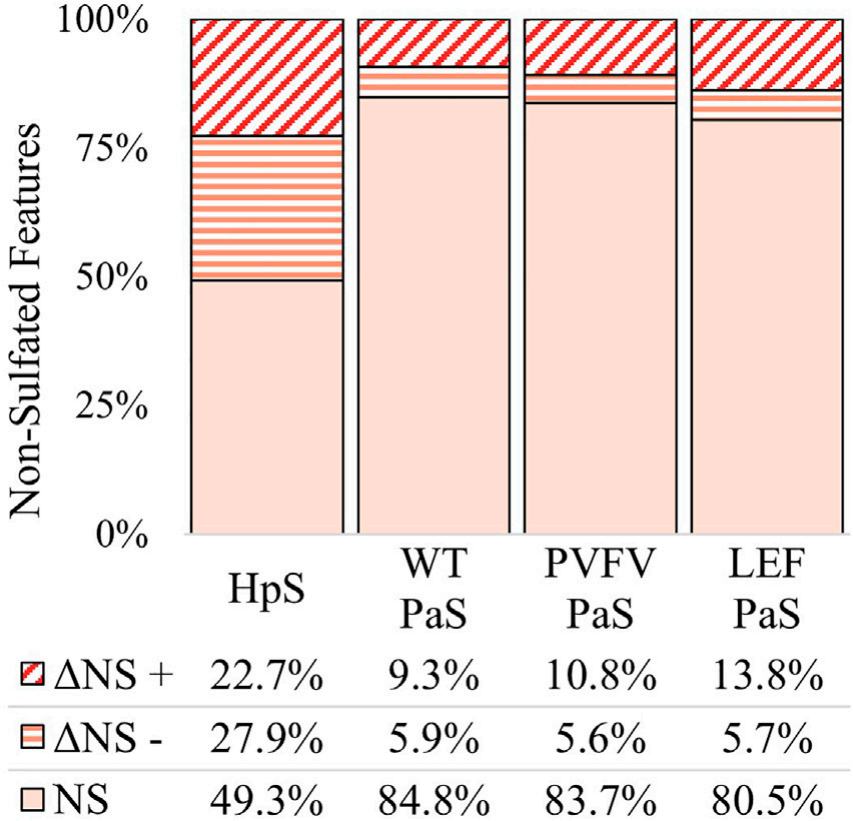
Total change in non-sulfated features for each sulfatase enzyme treatment of pooled human urine. Unchanged non-sulfated features are represented by NS where the adjusted *p*-value > 0.01. Changed non-sulfated features are represented by ΔNS, where adjusted *p*-value < 0.01. The direction of fold change is indicated by “−” or “+” signs. Values reported as a proportion (%) of total non-sulfated features (*n* > 4344).

The differential metabolite level analysis revealed that of the four enzyme treatments, the crude HpS preparation had distinct activity from the three PaS treatments (Figures 4–6). HpS possessed the largest number of significantly changed molecules in both the non-sulfated and sulfated fraction of the urinary metabolome. In the sulfated fraction (Figure 5), HpS had a total of 45% (646/1,430) of total sulfates significantly changed, which contrasts with the three PaS treatments displaying changes ranging from 30 to 34% (WT, 452/1430, PVFV, 433/1430, LEF, 481/1430). In the non-sulfated fraction (Figure 6), HpS resulted in 50.6% (2,202/4344) of molecules undergoing significant change compared to the PaS treatments, 15–20% (WT, 662/4344, PVFV, 710/4344, LEF, 848/4344).

In terms of enzyme hydrolysis, both HpS and the PaS enzymes showed a similar proportion (25.5–27.6%, Figure 5) of the sulfated features with a significant negative fold change, consistent with sulfatase enzyme hydrolysis. However, in the case of HpS treatment, this was accompanied by a greater proportion of sulfated features with a positive fold change (Figure 5) as well as a greater proportion of non-sulfated features undergoing both positive and negative fold change (Figure 6), which was inconsistent with simple sulfate ester hydrolysis and suggested significant levels of alternative enzyme activity. For the PaS enzyme treatments 82–87% (WT, 391/452, PVFV, 365/433, LEF, 365/481) of significantly changed sulfated features underwent a negative fold change consistent with enzyme hydrolysis compared to only 63% (394/646) for HpS treated samples (Figure 6 and Supplementary Table S3). Taken together, these observations show similar levels of sulfate hydrolysis for the four enzyme treatments but also clearly show higher selectivity for sulfate hydrolysis by the recombinantly expressed and purified PaS enzymes relative to the HpS crude enzyme preparation.

**FIGURE 7:**
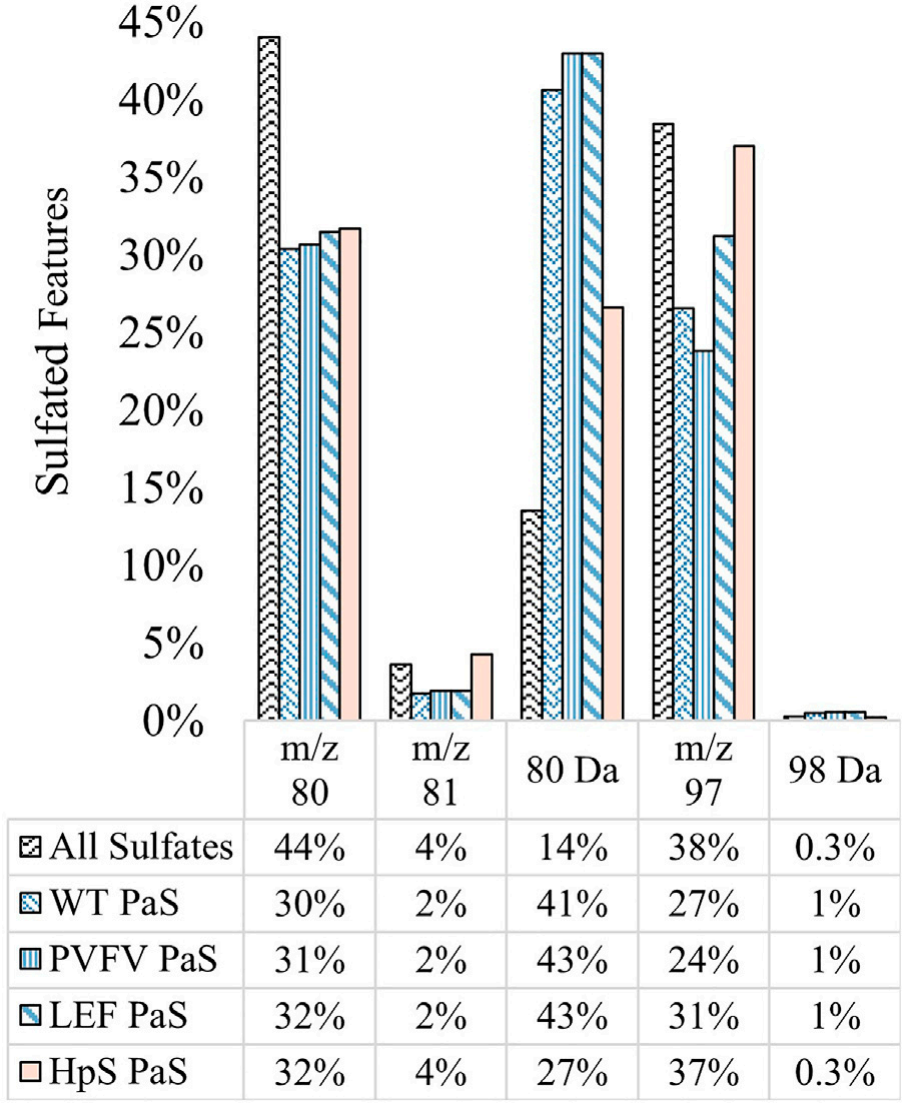
Speciation (%) of hydrolysed sulfates for each sulfatase enzyme treatment of pooled human urine. Hydrolysed sulfates (significant negative fold-change) for each enzyme treatment (WT, *n* = 391, PVFV, *n* = 365, LEF, *n* = 365, HpS, *n* = 394) are grouped according to their most abundant sulfate derived transition. This is contrasted with the speciation of all sulfates detected in pooled human urine (*n* = 1430). Note: neutral loss of 96 Da was not detected in pooled human urine.

The substrate scope for each sulfatase treatment was assessed by comparing the sulfate-derived fragment speciation for hydrolysed sulfates. Figure 7 shows the fragment speciation for all hydrolysed sulfates in comparison to the speciation of all detected sulfates (*n* = 1,430) in pooled human urine. Both enzyme classes displayed a preference for hydrolysis of sulfates, 27–43%, characterised by the neutral loss of SO_3_ (80 Da), when compared to the total proportion of sulfate species 14%. This may indicate a preference for the hydrolysis of electron deficient unsaturated sulfate esters, such as phenolic sulfates, through a process of bond homolysis (to give ^•^SO_3_^−^) followed by electron transfer to give SO_3_ and the corresponding oxy-anion fragment. There were also distinct differences between the two classes of enzyme treatments. Specifically, the PaS treatments hydrolysed a smaller proportion of saturated sulfates compared to the HpS treatment, as indicated by the proportion of the *m/z* 97 ion. Within the PaS enzyme classes, there was also a small observed increase in the hydrolysis of saturated sulfates (*m/z* 97) from the WT-PaS to the LEF-PaS mutant. This trend aligned with the aims of the previous directed evolution study, which sought to improve the catalytic efficiency of the PaS enzyme for the hydrolysis of the saturated alkyl sulfate ester testosterone sulfate (Uduwela et al., 2018).

#### Evidence for Glucuronidase Activity in Crude HpS Extract

The results clearly show the HpS enzyme had lower selectivity for the hydrolysis of sulfated metabolites, as a large proportion, 55% (1,214/2,202), of non-sulfated metabolites underwent significant change, Figure 6. A large part of this non-specific activity is attributable to the crude nature of the HpS extract that contains a range of enzyme activities including glucuronidase, oxidase and reductase activities (Stevenson et al., 2015; Ballet et al., 2018; Uduwela et al., 2018).

To investigate the possible glucuronidase activity of the crude HpS extract, a semi-targeted search was adapted in a retrospective fashion on the data acquired for application 2. The search was performed against a list of putative steroid glucuronides either derived from known steroids or from metabolic transformations of testosterone glucuronide, in a similar approach to that adopted in application 1 (Supplementary Table S8). Each of the 1,214 significantly changed non-sulfated features was matched in MS and MS/MS using accurate mass (±5 ppm) and assigned as glucuronides by matching to characteristic MS/MS transitions (Fabregat et al., 2013). These transitions included neutral loss of 194 Da (loss of glucuronic acid) and 176 Da [loss of glucuronic acid–H_2_O (gluc)], and the fragment ions *m/z* 175 [(gluc-H)^-^], *m/z* 157 [(gluc-H-H_2_O)^-^], *m/z* 113 [(gluc-H-H_2_O-CO_2_)^-^], *m/z* 85 [(Gluc-H-H_2_O-CO_2_-CO)^-^] and *m/z* 75 [(HOCH_2_CO_2_)^-^]. Unlike, Fabregat et al. (2013), matching was done at high resolution, allowing for accurate masses to be used in both MS and MS/MS dimensions (Supplementary Table S9).

From this search 96/1,214 molecules were identified as putative steroid glucuronides and had at least two characteristic MS/MS transitions. Of these, 90/96 underwent significant hydrolysis in the HpS treated sample, while none of these 96 molecules underwent hydrolysis in the PaS treatments (Supplementary Tables S8, S10)*.* The hydrolysis of glucuronide metabolites by HpS enzyme was not unexpected, with the product information sheet indicating at least 30 units of β-glucuronidase for every unit of sulfatase activity. Due to this, it has routinely been used in drug metabolism studies in both medical and anti-doping fields due to this broad substrate scope (Houghton and Maynard, 2010; Garg et al., 2018). A four-step purification of the crude HpS extract to generate higher selectivity for sulfate ester hydrolysis has recently been described, and the resulting preparation used to screen for unsaturated sulfate esters in human urine (Ballet et al., 2018). However, this purified HpS preparation is not commercially available. The PaS variants evaluated in this study show high levels of selectivity as expected for recombinantly expressed and purified enzymes and provide a convenient alternative for the study of the sulfated metabolome.

#### Substrate Selectivity of PaS

**FIGURE 8:**
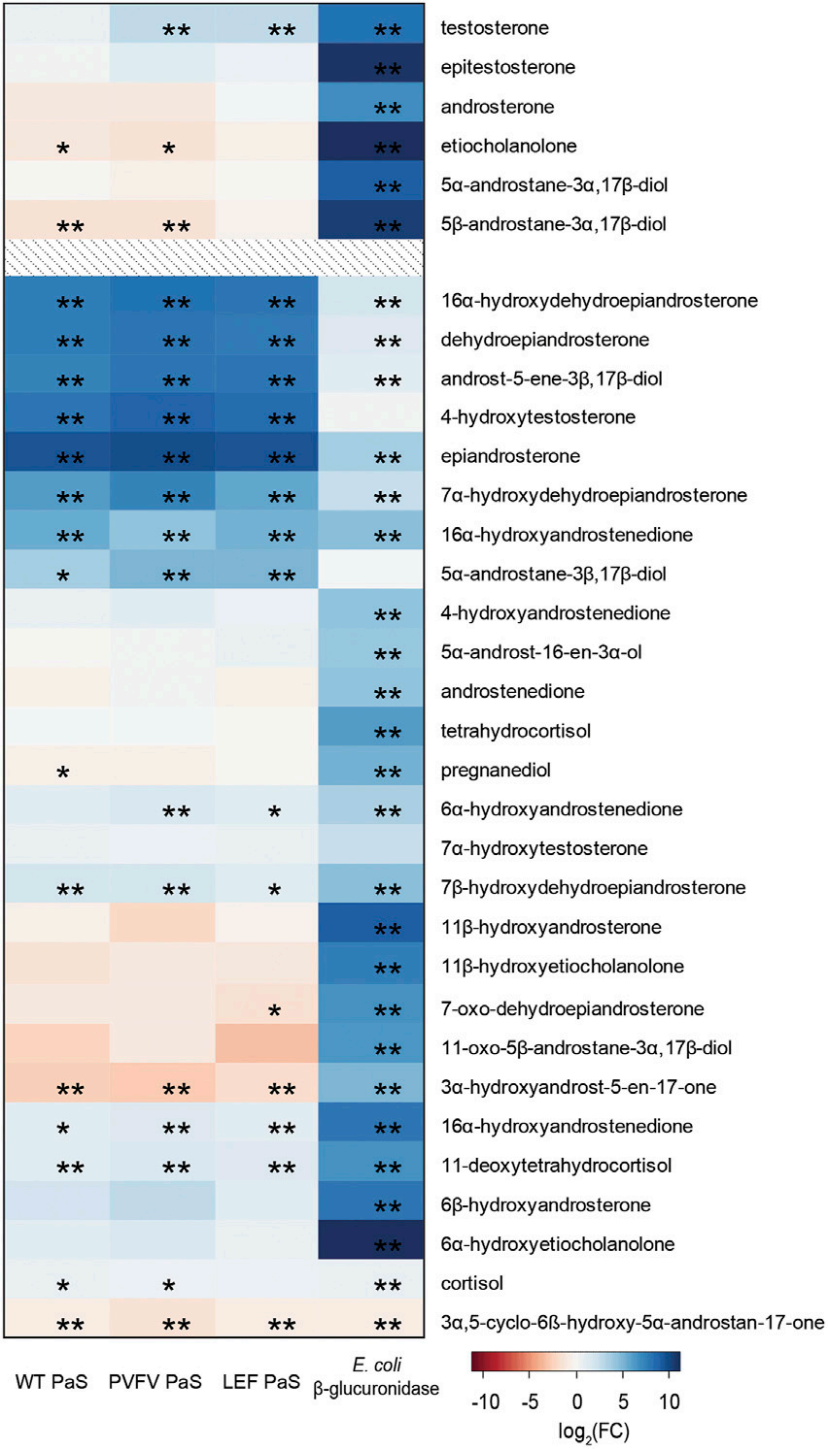
Heat map displaying the log_2_(FC) of the concentration (ng/mL) of 33 free steroids detected in GC-MS when treated with either WT PaS, PVFV PaS, LEF PaS or *E. coli* β-glucuronidase. The top 6 listed free steroids make up part of the steroid module in the athletes biological passport, all listed steroids are endogenously found in human urine. Adjusted *p*-values of *p* < 0.05 is denoted by “*”, and of *p* < 0.01 is denoted by “**”. Differential metabolite level analysis and the heat map was generated using R.

To demonstrate the selectivity of the PaS enzymes a targeted GC-MS analysis of free steroids was performed in treated urine (Figure 8, Supplementary Table S11). In this experiment we measured the concentrations of 33 free steroids in pooled human urine after treatment with either the PaS enzymes or with *Escherichia coli* (*E.coli*) β-glucuronidase (Supplementary Section S3). This β-glucuronidase was specifically chosen as that mandated for glucuronide deconjugation by the steroid module of the World Anti-Doping Agency athlete biological passport (World Anti-Doping Agency WADA, 2017). The concentration of 14 of these free steroids significantly increased after treatment with the PaS enzymes indicating some level of sulfate conjugation. This included testosterone sulfate that showed expected improvements in hydrolysis in moving from WT-PaS to PVFV-PaS and LEF-PaS mutants. Also, for these 14, the concentration of eight steroids, clustered in blue (Figure 8), showed a greater increase in concentration following PaS hydrolysis than observed for *E. coli* β-glucuronidase suggesting higher levels of sulfate conjugation. High levels of sulfate conjugation have also recently been reported for a range of circulating vitamin D metabolites (Jenkinson et al., 2021). These results were also compared against those from the metabolic profiling. Using an accurate mass search (*m/z* ± 5ppm) potential matches were found to each of these eight steroid sulfates in the UHPLC-HRMS/MS data set (Supplementary Table S12). Although the majority of these remain unconfirmed, epiandrosterone sulfate, was matched against an isotope labelled internal standard [epiandrosterone (^18^O_3_)-sulfate (**S3**)] used in the initial metabolic profiling. Epiandrosterone sulfate was found to undergo full hydrolysis for each enzyme treatment (Supplementary Table S13).

In this application we have demonstrated the selectivity of the PaS enzymes towards sulfated metabolites when compared to the commercially available crude HpS extract. As recombinantly expressed and purified enzymes, PaS is recommended as a preferred enzyme for studies of the sulfated metabolome. Overall, the results of this study demonstrate the usefulness of untargeted metabolic profiling methods to monitor minute differences in the sulfate metabolome in urine. Its strength lies in the deconvolution of sulfated from non-sulfated metabolites by monitoring sulfate-derived fragment ions.

## Conclusion

There are several clear pathways forward for this type of untargeted metabolic profiling. Due to its simplicity and relative ease of use it could be employed as a screen to look for new metabolites in applications such as in disease diagnosis or anti-doping. It is also conceivable that similar filters and scripts could be applied to related urinary metabolites such as glucuronides and phosphates, and di-anionic metabolites (McLeod et al., 2017). Limitations of this approach lie with the acquisition speeds of the MS instrumentation and the DDA method. The DDA method suffers from only sampling higher abundance ions per duty cycle, which can lead to missed detection or the misassignment of a molecule.

Overall, we have presented a novel workflow for the untargeted profiling of sulfated metabolites in urine matrices that combines UHPLC-HRMS/MS instrumentation and a new data processing pipeline. This provided a rapid tool for the qualitative assessment of the sulfate metabolome in equine and human urine. In equine urine 215 of 962 putative sulfate metabolites were found to significantly change after testosterone propionate administration in a single horse. Of these, 10 upregulated features were predicted to be steroid sulfate metabolites based on accurate mass searches. The identity of three steroid sulfates were confirmed as testosterone sulfate (**1)**, epiandrosterone sulfate (**2**), and a new metabolite, 5α-androstane-3β-17α-diol-3-sulfate (**3**), according to AORC retention time and MS/MS criteria (Association of Offical Racing Chemist, 2020). The profiling method was also used to examine sulfatase activity in pooled human urine. The new workflow identified 1,430 putative sulfated metabolite features in pooled urine from a total of 5,774 features. Qualitatively, it was observed that the three PaS enzymes selectively hydrolysed sulfate esters and may be preferred in applications targeting the sulfate metabolome. Alternative β-glucuronidase activity associated with the HpS enzyme was also demonstrated. The use of our profiling-based approach could be of value in the identification and monitoring of endogenous and exogenous sulfated metabolites in urine.

## Data Availability

The datasets presented in this study can be found in online repositories. The names of the repository/repositories and accession number(s) can be found in the article/Supplementary Material.
