# Supplementary material for: Profiling Urinary Sulfate Metabolites With Mass Spectrometry
Source: Front Mol Biosci. 2022 Feb 23;9:829511. doi: 10.3389/fmolb.2022.829511 (PMC8906285; doi:10.3389/fmolb.2022.829511)
Supplement: Supplementary file 1 [file DataSheet1.pdf]

Supplementary Information

Profiling Urinary Sulfate Metabolites with Mass Spectrometry

Table of Contents

S.1 Supplementary Figures 3

S.2 Supplementary Tables 12

S.3 Additional Methodology Details 34

S.4 Experimental for the Synthesis of Reference Materials and Stable Isotope Labelled Internal Standards 43

S.5 References 50

S.6. Spectra of synthesised standards 52

S.7 Script based data analysis 81

S.8 Data Availability 101

List of Figures

Figure S.1: 3
Figure S.2: 3
Figure S.3: 4
Figure S.4: 5
Figure S.5: 6
Figure S.6:........ 7
Figure S.7:........ 8
Figure S.8: 9
Figure S.9:........ 10
Figure S10:........ 11

List of Tables

Table S.1:........ 12
Table S.2:........ 16
Table S.3:........ 16
Table S.4:........ 17
Table S.5:........ 19
Table S.6:........ 21
Table S.7:........ 22
Table S.8:........ 23
Table S.9:........ 26
Table S.10:........ 27
Table S.11:........ 31
Table S.12:........ 32
Table S.13:........ 33
Table S.14:........ 36
Table S.15:........ 38

S.1 Supplementary Figures

During alignment, the raw data undergoes LOWESS normalisation followed by principal component analysis (PCA) in MS-DIAL. These functions are built into MS-DIAL. The generated PCA for each data set is shown below in Figures S.1 and S.2. The PCA plots are generated in R using the Scatterplot3D package.[1] The PCA analysis give an idea of how the data is segregated after initial alignment. All data was found to cluster within its biological triplicate sets. For both studies, the pooled quality control (QC) samples cluster tightly indicating good data quality.[2]

A)

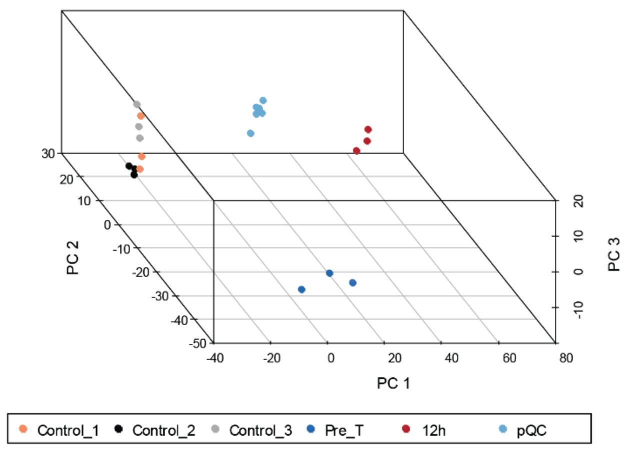

B)

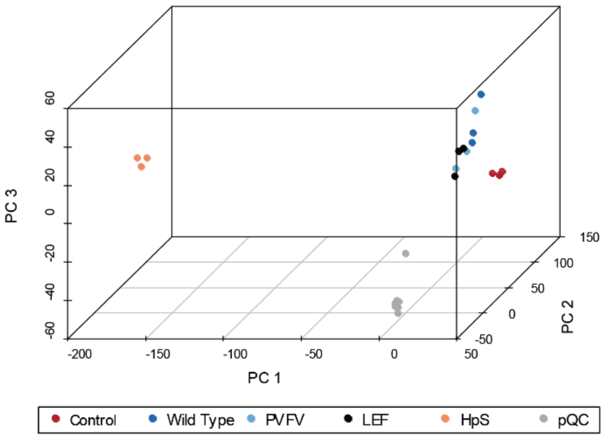

k-means clustering

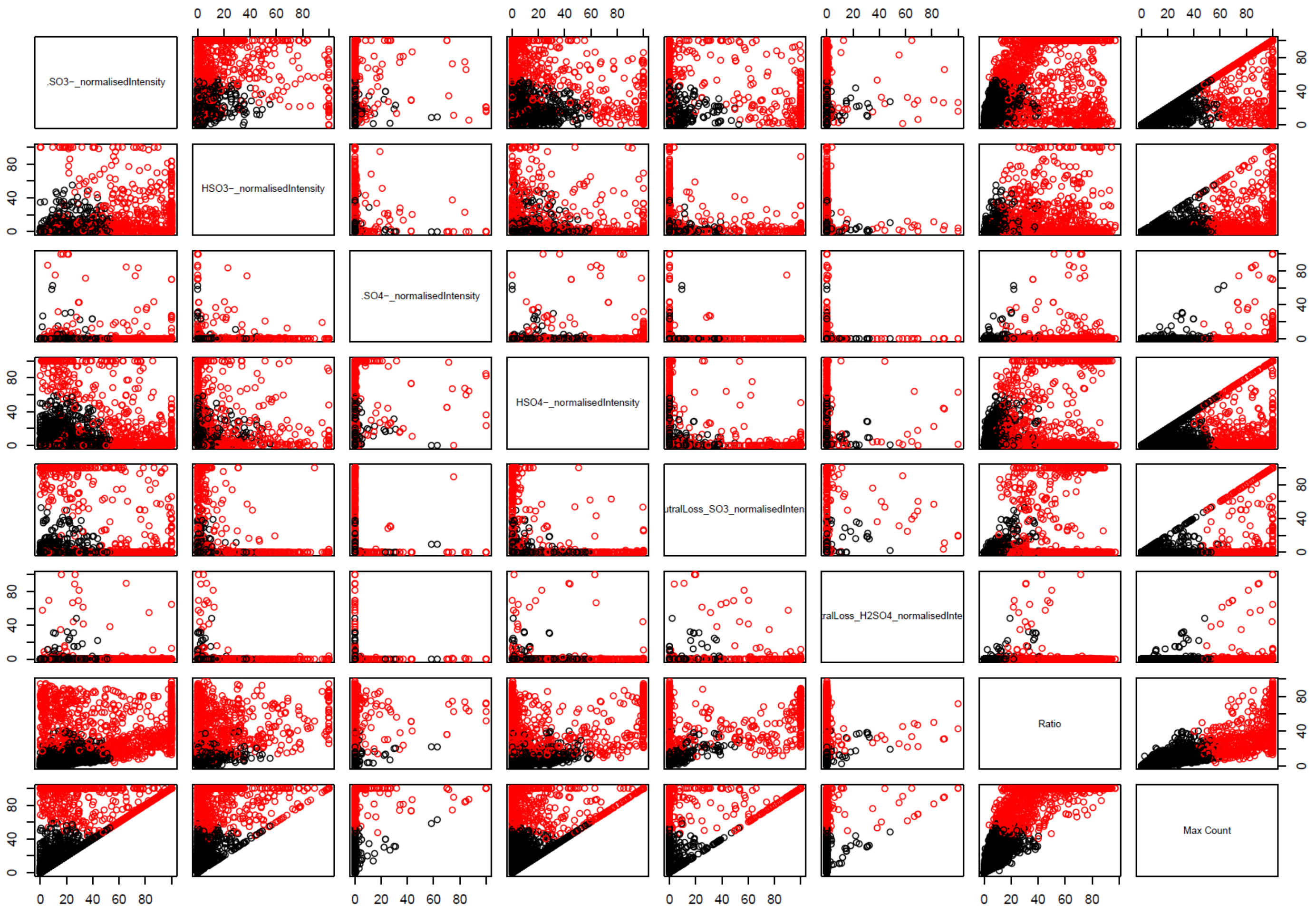

Figure S.3: Shows the *k-means* clustering into two groupings over the eight parameters in the equine urine drug administration study, application 1. Each box shows the distribution of sulfates (red) and non-sulfates (black) in pairwise comparisons of the clustering variables. Ratio and Max Count refer to the calculated quantities Intensity Ratio (IR) and Maximum Abundance (MA), respectively, as described in the methods section. In general, non-sulfates cluster towards the bottom left for all pairwise comparisons.

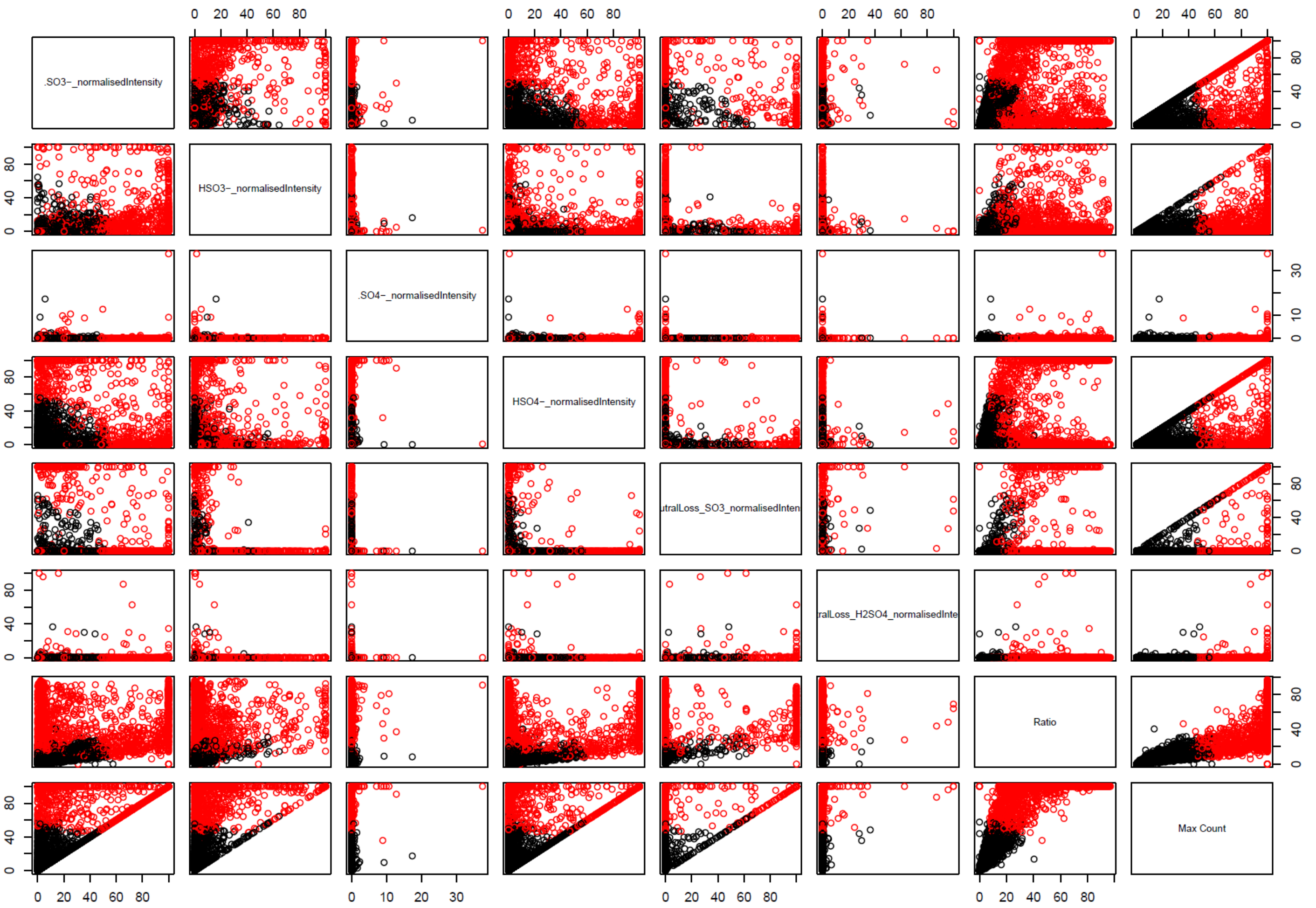

Figure S.4: Shows the *k-means* clustering into two groupings over the eight parameters in the human urine sulfatase enzyme study. Each box shows the distribution of sulfates (red) and non-sulfates (black) in pairwise comparisons of the clustering variables. Ratio and Max Count refer to the calculated quantities Intensity Ratio (IR) and Maximum Abundance (MA), respectively, as described in the methods section. In general, non-sulfates cluster towards the bottom left for all pairwise comparisons.

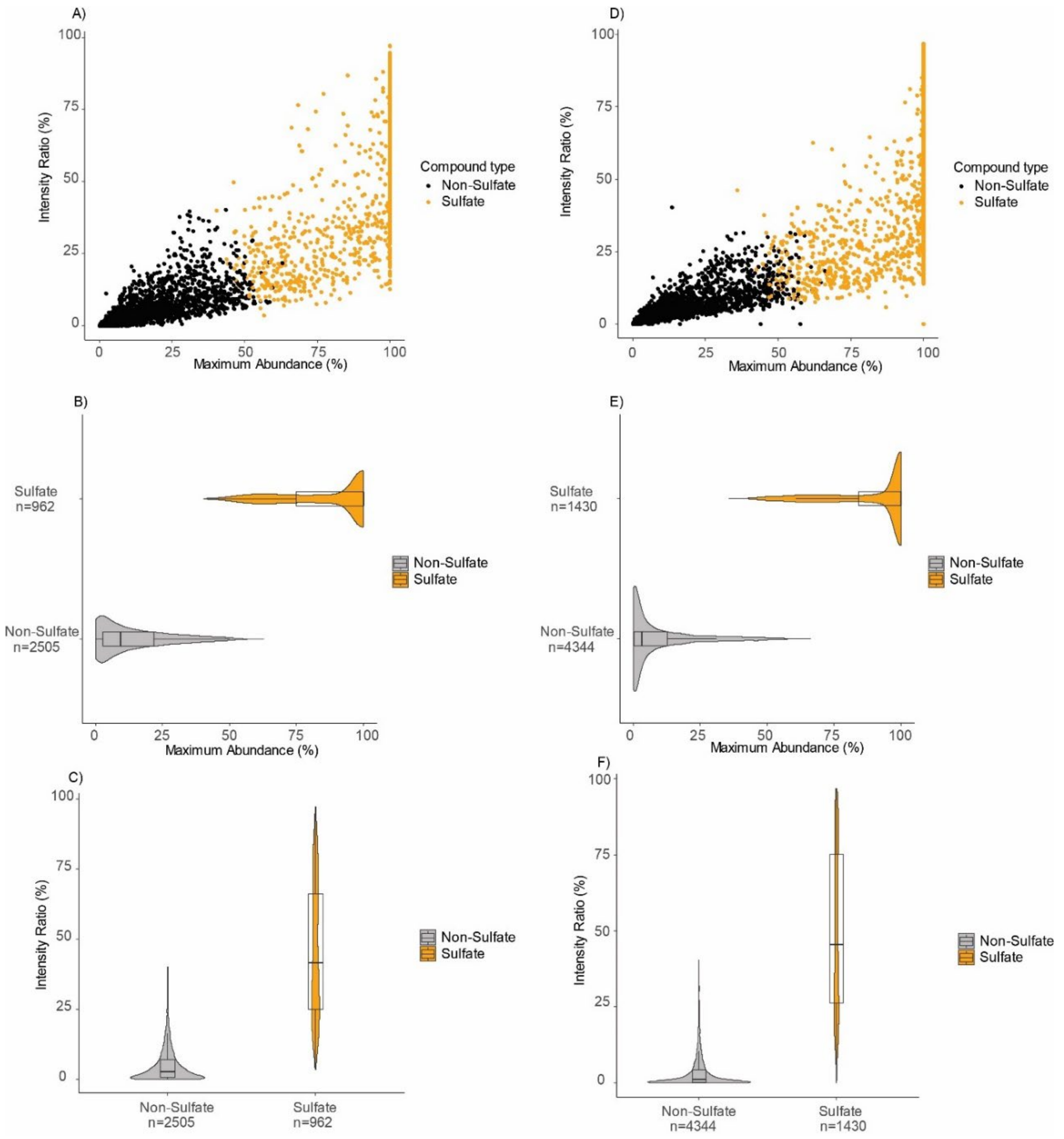

Figure S.5: *k-means* clustering plots of both the equine testosterone administration study and the sulfatase enzyme study. Molecules were clustered into two groups using the *k-means* clustering algorithm in R.[3] Clustering was based on six sulfate-derived fragment ions in MS/MS spectra (*m/z* 80, 81, 96, 97, and neutral losses of 80 Da and 98 Da) and the MA and IR values for each molecule. A) Clustering plot contrasting MA against IR of sulfated and non-sulfated molecules found in the equine urine drug administration study. B) The spread of MA values for each molecule in equine urine, the median of sulfated molecules was 99% MA (mean = 88%) and for the non-sulfated molecules 9% MA (mean = 5%). C) The spread of IR values for each molecule in equine urine, the median of sulfated molecules was 42% IR (mean = 46%) and for non-sulfated molecules 3% IR (mean = 5%). D) Clustering plot contrasting MA against IR of sulfated and non-sulfated molecules found in the sulfatase hydrolysis study in pooled human urine. E) The spread of MA values for each molecule in detected in the sulfatase treated urine, the median of sulfated molecules was 100% MA (mean = 90%) and 3% MA (mean = 9%) for the non-sulfated molecules. F) The spread of IR values for each molecule in the sulfatase treated urine, the median of sulfated molecules was 46% IR (mean = 50%) and for non-sulfated molecules 1% IR (mean = 3%).

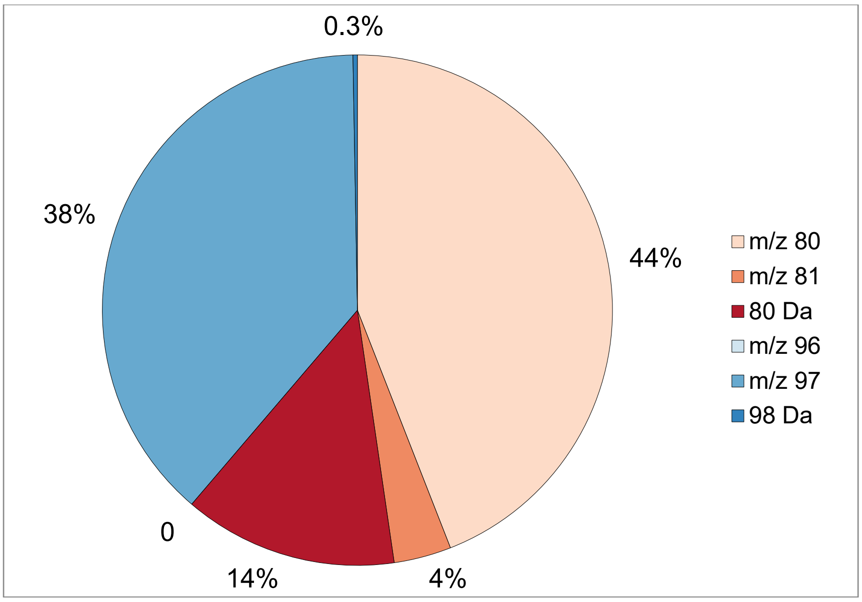

Figure S.6: Sulfate speciation for all putative sulfates (n=1430) in the sulfatase hydrolysis study in pooled human urine. The sulfates are categorised by their highest abundance sulfate-derived fragment ion. Speciation is as follows, *m/z* 80 (*n* = 630, 44%), *m/z* 81 (*n* = 52, 3.6%), neutral loss 80 Da (*n* = 194, 13.6%), *m/z* 97 (*n* = 550, 38.5%), neutral loss 98 Da (*n* = 4, 0.3%). Note, no *m/z* 96 was detected in the pooled human urine samples.

High throughput differential metabolite level analysis

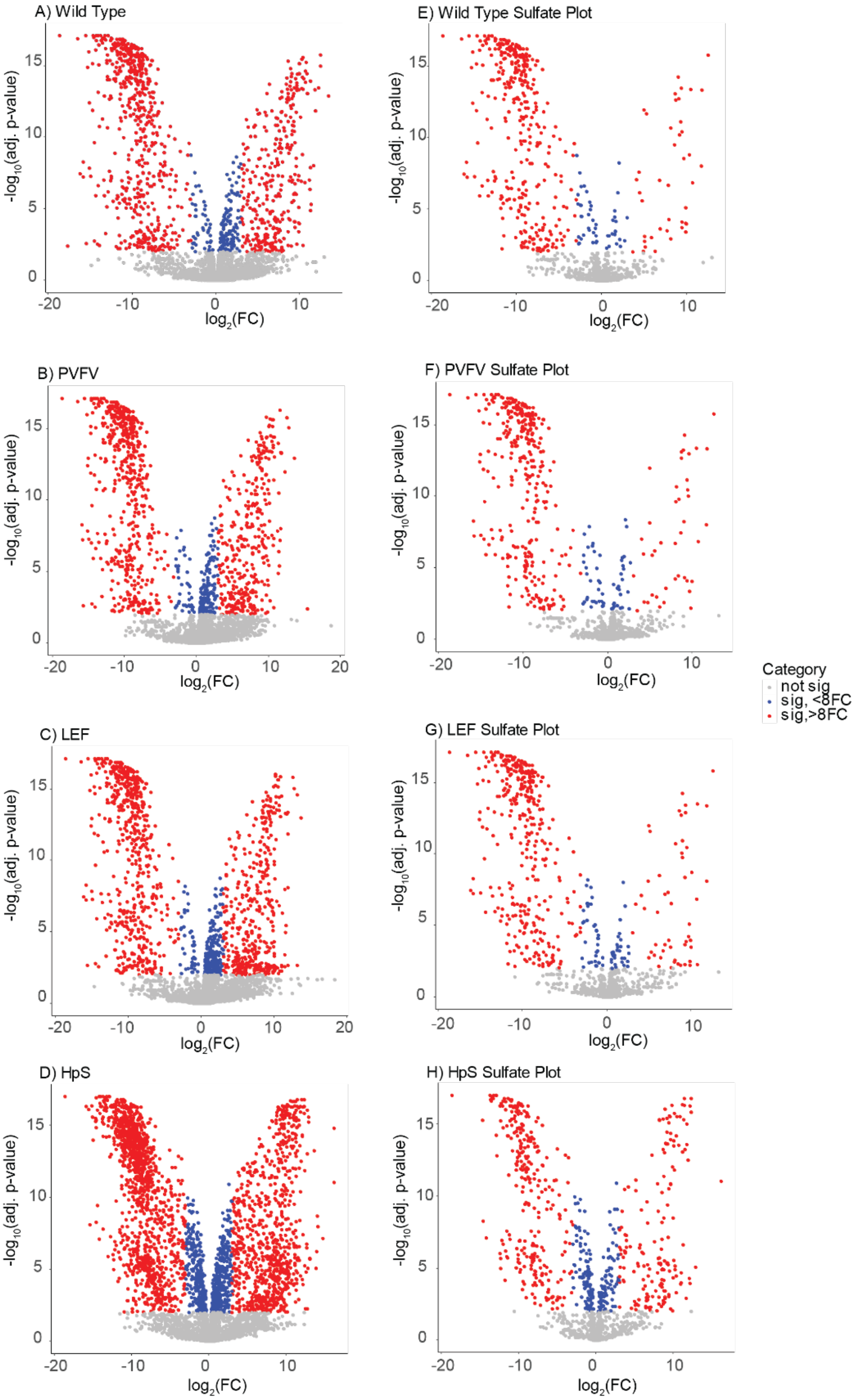

Figure S.7: A-D) Volcano plots comparing treatment of pooled human urine with *Pseudomonas aeruginosa* arylsulfatase (PaS) enzymes WT-PaS, PVFV-PaS, LEF-PaS and *Helix pomatia* arylsulfatase (HpS), relative to a control sample. The $\log_2$ fold change and $-\log_{10}$ of the adjusted p-value is shown for each detected metabolite (n = 5774). **E-H)** Volcano plots of sulfate metabolites (n = 1430) identified through *k-means* clustering for the same enzyme treatments.

Variation between Horse 1 (doped) and Horse 2 (non-doped control).

Intra and inter horse variability was assessed by contrasting all samples against the horse 1 -24 h sample (Figure S.8) and against the horse 2 T_1 sample (Figure S.9). The results in Figure S.9 suggest that there is greater variability between (inter) the urine of different horses than within (intra) the same horse. This makes the variability observed in Figure S.8 between the -12h and plus 12h sample likely a result of testosterone propionate administration.

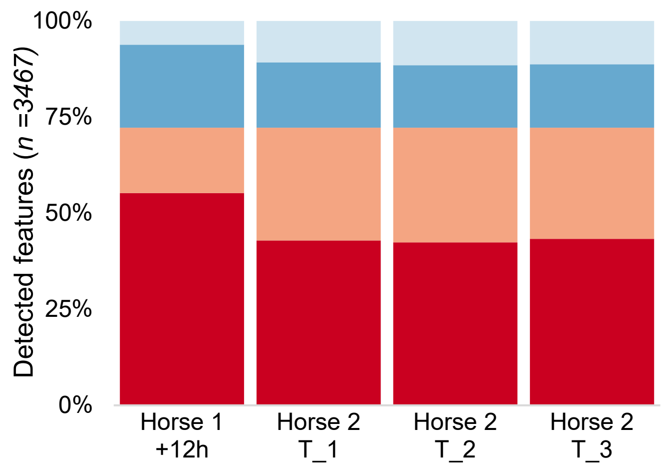

	Horse 1 +12h	Horse 2 T_1	Horse 2 T_2	Horse 2 T_3
ΔS	6%	11%	12%	11%
S	22%	17%	16%	16%
ΔNS	17%	29%	30%	29%
NS	55%	43%	42%	43%

Figure S.8: Total change in detected features in equine urinary metabolome in application 1. The plus 12 h sample is plotted with three non-doped control samples (horse 2) with all comparisons to the horse 1 minus 24 h sample. Features were split into non-sulfate (NS, red) and sulfate (S, blue) groups. Unchanged features are represented by S and NS, where adjusted $p > 0.01$. Changed features are represented by ΔS and ΔNS, where adjusted p-value < 0.01. Reported as a proportion (%) of total detected features ($n = 3467$). See table S.3 for further detail. The plot shows greater inter-horse variability (horse 2) than intra-horse variability (horse 1) following testosterone propionate administration.

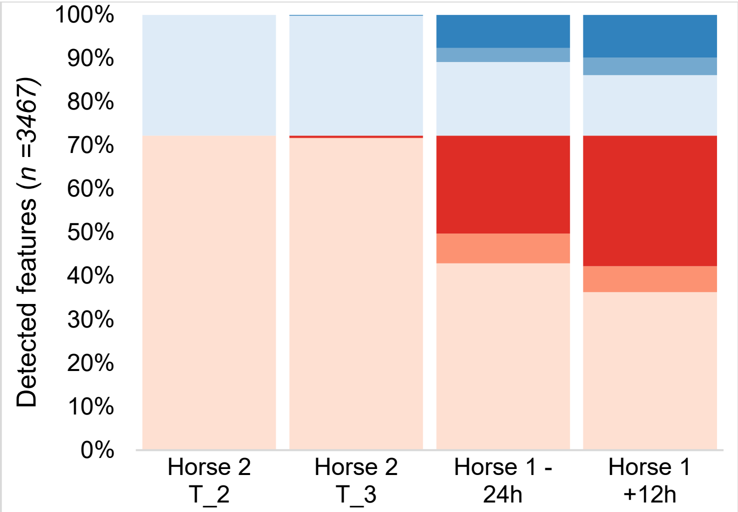

	Horse 2 T_2	Horse 2 T_3	Horse 1 -24h	Horse 1 +12h
ΔS +	0%	0%	8%	10%
ΔS -	0%	0%	3%	4%
S	28%	28%	17%	14%
ΔNS +	0%	1%	22%	30%
ΔNS -	0%	0%	7%	6%
NS	72%	72%	43%	36%

Figure S.9: Total change in detected features in equine urinary metabolome in application 1 The horse 2 time point 2 (T_2) and time point 3 (T_3) samples are plotted alongside the horse 1 minus 24 h (-24 h) and plus 12 h (+12 h) samples, with all comparisons to horse 2 time point 1 (Horse 2 T_1). Unchanged features are represented by S and NS, where $p > 0.01$. Changed features are represented by ΔS and ΔNS, where adjusted p-value < 0.01. Reported as a proportion (%) of total detected features ($n = 3467$). The plot shows lower inter-horse variability (horse 2) than intra-horse variability (horse 1) both before and after testosterone propionate administration.

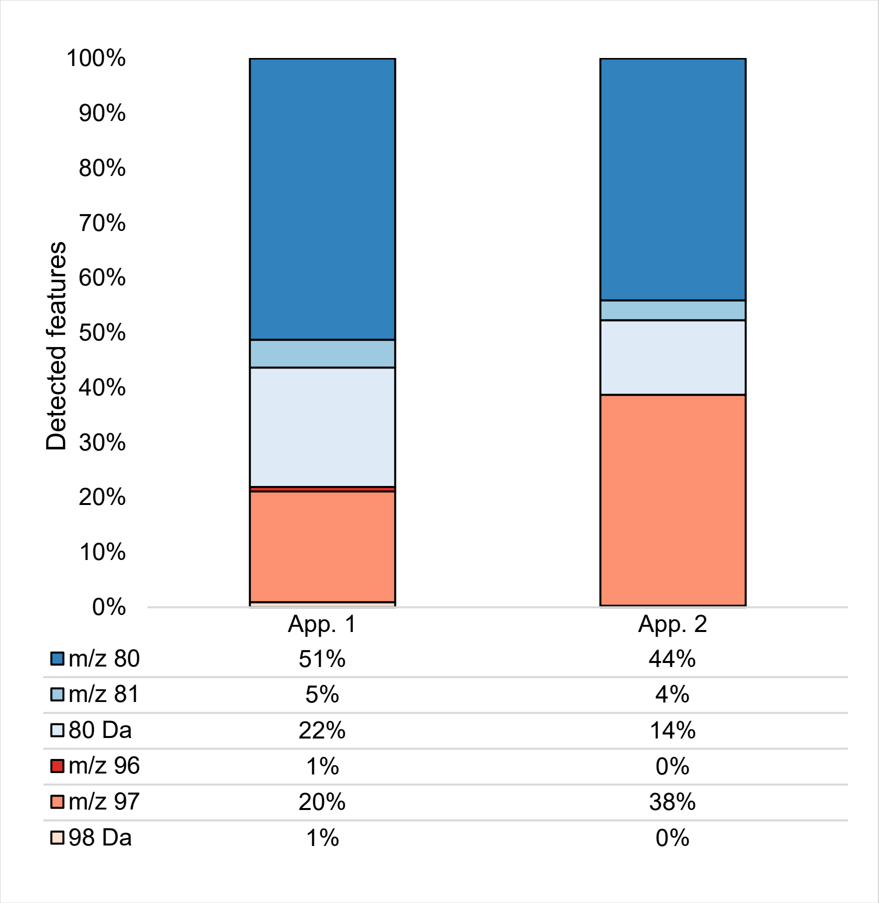

Figure S10: The differences in speciation between equine urine (n= 962) and pooled human urine (n=1430) as a percentage of detected sulfated features in each study.

S.2 Supplementary Tables

HPLC-MS/MS method development

A collection of reference materials comprised of ten mono-sulfate and eight bis(sulfate) compounds were used to develop the chromatographic method and to select suitable fragmentation energies (Table S.1). All were prepared as ammonium salts, except deuterated testosterone sulfate, which was a triethylammonium salt. Nandrolone sulfate, deuterated testosterone sulfate and cholanediol bis(sulfate) were used as internal standards for method development or metabolite confirmation. Three separate solutions were prepared, containing a mixture of non-isomeric reference compounds at 300 ng/mL in methanol:water (80:20, v/v), since some of the isomeric compounds co-eluted during chromatography. Reference compounds listed in Table S.1 had been previously prepared or were synthesised for the express purpose of this study.[4]

Table S.1: Fragmentation of reference compounds with relative abundance (%) of precursor and sulfate-derived fragment ions at a normalised collision energy 40 and 60.

Sulfate<br>(Elemental comp.)<br>[CAS number]	Structure	*m/z*	NCE	Precursor	*m/z* 80	*m/z* 81	*m/z* 96	*m/z* 97	NL 80 DA or 98 Da
3β,11β-dihydroxy-5α-androstan-17-one bis(sulfate)<br>$(C_{19}H_{30}O_9S_2)$<br>[2099023-13-7]	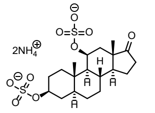	232.0593	40	100	0	0	0	81	0
			60	1.5	8.2	0	0	100	0
estradiol bis(sulfate)<br>$(C_{18}H_{24}O_8S_2)$<br>[3233-70-3]	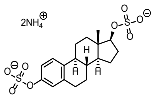	215.0384	40	58	100	0.26	0	27	25
			60	0	100	0	29	56	14
androst-5-ene-3β,17β-diol bis(sulfate)<br>$(C_{19}H_{30}O_8S_2)$<br>[21861-04-1]	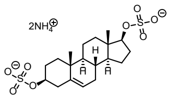	224.0618	40	41	0	0	0	100	0
			60	0	2.9	0	0.47	100	0
5α-androstane-3β,17β-diol bis(sulfate)<br>$(C_{19}H_{32}O_8S_2)$	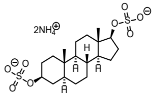	225.0697	40	100	0	0	0	59	0
			60	13	2.4	0	0	100	0

[55928-42-2]

Table S.1 (continued)

5α-pregnane-3β,*20S*-diol bis(sulfate)<br>($C_{21}H_{36}O_8S_2$)<br>[27935-53-1]	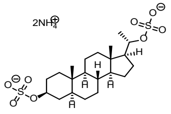	239.0853	40	49	0.35	0	0	100	0
			60	0.1	1.5	0	0	100	0
5β-androstane-3α,17β-diol bis(sulfate)<br>($C_{19}H_{32}O_8S_2$)<br>[55928-43-3]	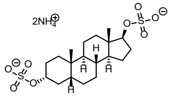	225.0697	40	100	0	0	0	53	0
			60	2.5	2.9	0	0	100	0
5α-androstane-3α,17β-diol bis(sulfate)<br>($C_{19}H_{32}O_8S_2$)<br>[21152-49-8]	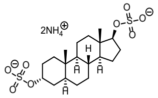	225.0697	40	77	0	0	0	100	0
			60	0.18	59	0	0.52	100	0
estrone sulfate<br>($C_{18}H_{22}O_5S$)<br>[481-97-0]	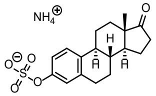	349.1115	40	96	31	14	0	0	96
			60	0.98	22	3.5	0	0	100
nandrolone sulfate<br>($C_{18}H_{26}O_5S$)<br>[98804-55-8]	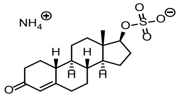	353.1428	40	100	0	0	0	14	0
			60	1.5	8.2	0	0	100	0
deuterated testosterone sulfate<br>($C_{19}H_{25}D_3O_5S$)<br>[361432-51-1]	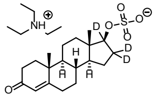	370.1773	40	100	0	0	0	5.1	0
			60	46	7.5	0	0	100	0
testosterone sulfate<br>($C_{19}H_{28}O_5S$)<br>[651-45-6]	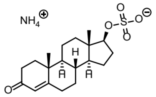	367.1585	40	100	0.42	0	0	8.0	0
			60	30	6.5	0	0	100	0

Table S.1 (continued)

dehydroepiandrosterone sulfate ($C_{19}H_{28}O_5S$) [651-48-9]	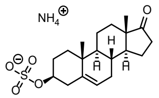	367.1585	40	33	0	0	0	100	0
			60	0.13	1.9	0	0	100	0
epitestosterone sulfate ($C_{19}H_{28}O_5S$) [4579-56-0]	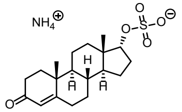	367.1585	40	99	0	0	0	95	0
			60	1.4	1.8	0	0.49	100	0
5α-androstane-3β,17β-diol 3-sulfate ($C_{19}H_{32}O_5S$) [17605-24-2]	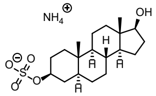	371.1898	40	100	0	0	0	23	0
			60	11	1.3	0	0	100	0
dihydrotestosterone sulfate (17β-hydroxy-5α-androstan-3-one) ($C_{19}H_{30}O_5S$) [2641-48-7]	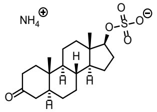	369.1741	40	100	0.18	0	0	8.6	0
			60	41	1.8	0	0	100	0
5α-androstane-3α,17β-diol 3-sulfate ($C_{19}H_{30}O_5S$) [1048-59-5]	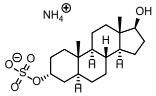	371.1898	40	100	0	0	0	35	0
			60	2.0	2.4	0	0	100	0
5β-androstane-3α,17β-diol 3-sulfate ($C_{19}H_{30}O_5S$)	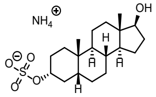	371.1898	40	100	0	0	0	39	0
			60	4.0	1.1	0	0	100	0
androsterone sulfate ($C_{19}H_{30}O_5S$) [977-36-6]	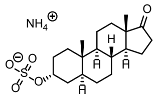	369.1741	40	100	0	0	0	56	0
			60	3.5	1.4	0	0	100	0

Table S.1 (continued)

cholanediol bis(sulfate) (5β-cholane-3α,24-diol bis(sulfate) ($C_{24}H_{42}O_8S_2$) [66450-87-1]	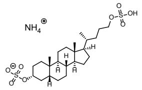	260.1088	40	100	0	0	0	72	0
			60	0.2	2.1	0	0	100	0
lithocholic acid sulfate ($C_{24}H_{40}O_6S$) [64936-81-8]	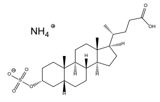	455.2473	40	94	0	0	0	100	0
			60	0	1.9	0	0	100	0

Table S.2: Descriptive statistics of the spread of non-sulfates and sulfates in the IR and MA parameters in applications (App.) 1 and 2. For a visual representation see Figures 1 & S.7. *SD= Standard deviation, IQR = Interquartile range. Calculated in the base package of R.[5]

	Parameter	Cluster	SD	Mean	Median	IQR	%25 Q	%50 Q	%75 Q
App.1	IR	non-sulfate	6	5	3	6	1	3	7
		sulfate	24	46	42	41	25	42	66
	MA	non-sulfate	13	14	9	19	3	9	22
		sulfate	17	88	100	25	75	100	100
App.2	IR	non-sulfate	5	3	1	4	0	1	4
		sulfate	26	50	46	49	26	46	75
	MA	non-sulfate	13	9	3	12	0	3	13
		sulfate	15	90	100	16	84	100	100

Table S.3: High throughput differential metabolite level analysis of non-sulfates (NS) and sulfated (S) features in both applications. *Δ denotes significantly changed features with an adjusted p-value < 0.01. The direction of fold change is indicated by "-" or "+" signs.

**Application 1**							
**Biological Sample**	**NS**	**S**	**ΔNS -**	**ΔNS +**	**ΔS -**	**ΔS +**	**Total Features**
**Plus 12 hours**	1915	747	141	449	79	136	3467
**Horse 2_T1**	1488	588	779	238	263	111	3467
**Horse 2_T2**	1470	562	748	287	254	146	3467
**Horse 2_T3**	1503	571	698	304	232	159	3467
**Application 2**							
**HpS**	2142	784	1214	988	394	252	5774
**WT-PaS**	3682	978	258	404	391	61	5774
**PVFV-PaS**	3634	997	243	467	365	68	5774
**LEF-PaS**	3496	949	249	599	395	86	5774

Table S.4: Theoretical structures used to search for potential steroid sulfate metabolites of testosterone propionate in application 1. Predicted structures consist of up to 3x phase I metabolic transformations of testosterone plus sulfation. "Masses found in screen" indicate if and how many times the accurate mass was detected as a metabolic feature in the metabolomic profiling. All matches were performed to a mass tolerance of ±5 ppm. A total of 30 detected metabolic features matched the accurate masses (±5 ppm) of the hypothetical structures below. All matches were performed in R using base code.[5]

Masses found in Screen	Chemical Formula	Possible Theoretical structure	Transformation	Theoretical accurate mass (*m/z*)
2	$C_{19}H_{27}O_5S^-$	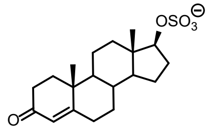	No change	367.1583
0	$C_{19}H_{25}O_5S^-$	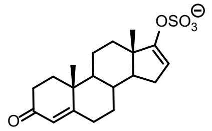	1 x Oxidation (enolation)	365.14227
3	$C_{19}H_{29}O_5S^-$	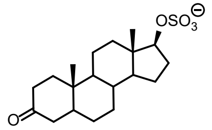	1 x Reduction	369.1739
1	$C_{19}H_{31}O_5S^-$	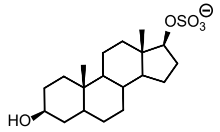	2 x Reduction	371.1895
0	$C_{19}H_{23}O_6S^-$	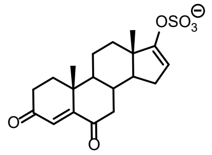	1 x Hydroxylation 2 x Oxidation (enolation)	379.1215
2	$C_{19}H_{25}O_6S^-$	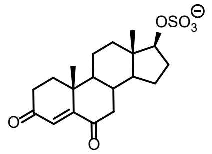	1 x Hydroxylation 1 x Oxidation	381.1370

3	$C_{19}H_{27}O_6S^-$	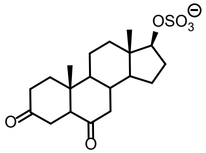	1 x Hydroxylation<br>1 x Reduction<br>1 x Oxidation	383.1528
4	$C_{19}H_{29}O_6S^-$	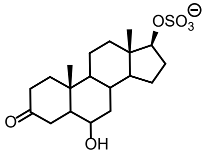	1 x Hydroxylation<br>1 x Reduction	385.1685
9	$C_{19}H_{31}O_6S^-$	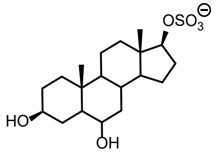	1 x Hydroxylation<br>2 x Reduction	387.1841
0	$C_{19}H_{25}O_7S^-$	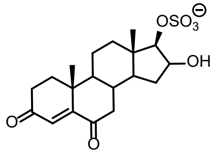	2 x Hydroxylation<br>1 x Oxidation	397.1321
2	$C_{19}H_{27}O_7S^-$	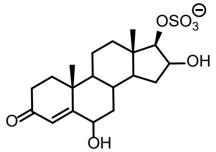	2 x Hydroxylation	399.1478
4	$C_{19}H_{29}O_7S^-$	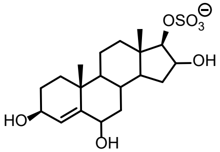	2 x Hydroxylation<br>1 x Reduction	401.1634
0	$C_{19}H_{27}O_8S^-$	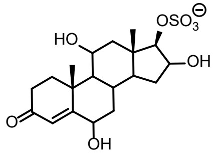	3 x Hydroxylation	415.1427

Table S.5: List of 30 out of 215 putative steroids which matched a theoretical steroid sulfate structure described in Table S.4. The Log_2FC and adjusted p-values for each molecule is shown in the +12 h (horse 1) and the three control samples (horse 2). * Denotes putative steroids that were omitted from the final list due to similarity with levels in the control horse 2. This gave a final list of 10 final putative structures as seen in Table 2 in the manuscript.

**Equine urine sample**				**Log_2FC**				**Adjusted p-value**					
**RT [min]**	***m/z***	**S/N average**	**theoretical *m/z* (± 5 ppm)**	**Horse 1 +12h**	**Horse 2 T_1**	**Horse 2 T_2**	**Horse 2 T_3**	**Horse 1 +12h**	**Horse 2 T_1**	**Horse 2 T_2**	**Horse 2 T_3**	**Cluster**	
8.57	367.1581	69	367.1583	3	1	1	1	5E-01	9E-01	8E-01	8E-01	2	*
8.77	367.1583	137	367.1583	10	-2	-1	0	2E-07	3E-02	2E-01	9E-01	2	
9.96	369.1738	14	369.1739	2	3	1	1	4E-01	1E-01	5E-01	6E-01	2	*
7.69	369.1739	11	369.1739	9	-1	-1	-1	6E-06	2E-01	2E-01	2E-01	2	
10.16	369.1739	26	369.1739	6	-2	-1	-1	5E-06	4E-02	3E-01	4E-01	2	
10.53	371.1895	3	371.1895	8	-1	-1	1	2E-05	2E-01	6E-01	6E-01	2	
7.05	381.1370	14	381.1370	-6	2	2	3	9E-07	6E-03	4E-03	3E-04	2	*
6.95	381.1373	13	381.1370	-6	2	2	3	1E-09	7E-06	8E-06	9E-07	2	*
7.66	383.1531	5	383.1528	0	1	2	3	1E+00	6E-01	5E-01	2E-01	2	*
7.50	383.1536	4	383.1528	1	-2	-2	0	3E-01	3E-02	3E-02	7E-01	2	*
6.37	383.1537	14	383.1528	7	-1	-2	-3	1E-03	5E-01	2E-01	6E-02	2	
5.65	385.1688	3	385.1685	7	6	4	5	3E-04	6E-04	4E-03	1E-03	2	*
5.89	385.1690	14	385.1685	-1	5	5	6	4E-01	2E-05	1E-05	2E-06	2	*
6.74	385.1690	4	385.1685	4	0	0	1	7E-08	7E-01	7E-01	4E-02	2	
7.41	385.1692	3	385.1685	0	-2	-2	-1	4E-01	9E-05	1E-04	9E-03	2	*
5.48	387.1845	25	387.1841	5	7	9	10	1E-02	7E-04	1E-04	5E-05	2	*
7.59	387.1845	14	387.1841	10	-3	-1	-3	2E-05	4E-02	3E-01	4E-02	2	
7.39	387.1846	9	387.1841	8	0	1	1	8E-03	1E+00	7E-01	8E-01	2	
6.32	387.1846	12	387.1841	6	8	8	9	2E-09	3E-10	3E-10	9E-11	2	*
6.14	387.1846	8	387.1841	-1	0	0	1	7E-03	8E-01	1E+00	7E-02	2	*
6.45	387.1846	9	387.1841	0	7	7	9	1E+00	3E-10	3E-10	7E-11	2	*
4.56	387.1846	9	387.1841	9	-3	-3	-3	5E-05	2E-02	2E-02	2E-02	2	
5.60	387.1847	23	387.1841	8	10	10	11	1E-06	4E-08	3E-08	1E-08	2	*

4.48	387.1849	9	387.1841	9	-3	-3	-3	2E-05	2E-02	2E-02	2E-02	2	
8.41	399.1480	5	399.1478	1	6	5	7	5E-01	7E-04	2E-03	2E-04	2	*
8.56	399.1483	3	399.1478	0	6	11	9	1E+00	2E-03	4E-05	1E-04	2	*
4.37	401.1634	13	401.1634	5	4	8	10	2E-02	2E-02	7E-04	1E-04	2	*
4.54	401.1635	7	401.1634	4	8	10	10	6E-02	2E-04	8E-05	8E-05	2	*
3.90	401.1636	9	401.1634	1	-2	-1	0	5E-01	1E-01	3E-01	9E-01	2	*
4.11	401.1640	8	401.1634	9	8	10	8	6E-05	6E-05	2E-05	6E-05	2	*

Table S.6: Confirmation of testosterone sulfate (**1**), epiandrosterone sulfate (**2**) and 5α-androstane-3β,17α-diol 3-sulfate (**3**) metabolites derived from the sulfate fraction in equine urine (horse 1, t = 12 h) according to AORC, RT and MS/MS criteria.[7]

	**Reference material**			**Metabolite**	
**Compound**	**Relative Retention Time (min) [theoretical m/z]**	**[a]Product ion (*m/z*)**	**[b]Relative abundance (%)**	**Relative Retention Time (min) [tolerance]**	**[b]Relative abundance (%) [%tolerance]**
**Testosterone Sulfate (1)**	0.87 [367.1583]	367.1583	36	0.87 [0.86-0.89]	34 [16-56]
		177.0224	3		3 [0-23]
		96.9600	100		100 [60-100]
		79.9573	6		6 [0-26]
**Epiandrosterone Sulfate (2)**	1.00 [369.1739]	369.1735	7	1.00 [0.98-1.02]	6 [0-27]
		96.96	100		100 [60-100]
		79.9573	2		2 [0-22]
**5α-androstane-3β,17α-diol 3 sulfate (3)**	1.04 [371.1895]	371.1906	11	1.04 [1.02-1.06]	10 [0-31]
		96.9601	100		100 [60-100]
		79.9574	1		4 [0-21]

[a] All product ions match in a mass tolerance window of ±5 ppm. [b]Derived from the peak area of extracted ion chromatograms from data acquired in parallel reaction monitoring MS/MS experiments (NCE 60).

Spectrophotometry for para-nitrophenol (PNP)

This method was based on previous work in the literature.[8] Unless otherwise stated, the buffers used for screening and assays are as follows: 50 mM Tris-HCl for buffering at pH 7.5 and 50 mM sodium acetate for buffering at pH 5. The pH of buffers was measured using a pH 700 meter and probe from Eutech Instruments (Singapore). The 50 mM Tris-HCl buffer was used for the determination of PaS enzyme activity, and 50 mM sodium acetate used for the HpS enzyme, based on previous activity assays in literature.[8,9]

Hydrolysis of PNPS was monitored by measuring the absorption of 405 nm light by the PNP anion. Assays were performed in 96-well plate format with filled wells to obtain a 10 mm path length. Reactions or standards (300 µL) were mixed with 50 µL of 1N KOH to ensure complete ionization of PNP and to quench enzyme activity. The extinction coefficient for PNP with this method was 14,147 M^{-1} cm^{-1}, and relative activity determined against a PNP standard curve, ranging in concentration from 0 to 40 µM (R^2 >0.9975), at a pH of 5 and 7.5 respectively. All enzyme concentrations used in this research were normalised according to the activity observed in Table S.7.

Table S.7: The relative hydrolysis activity of PNPS to PNP of all sulfatase enzymes

	WT-*PaS*	PVFV-*PaS*	LEF-*PaS*	HpS
**µM/min**	0.59	0.44	0.66	0.75
**Final Conc µg/mL**	0.34	0.34	0.34	9.80

Table S.8: Theoretical structures used to search for potential steroid glucuronide metabolites in human urine. The search was based on the accurate mass of predicted structures or known structures of steroid glucuronides. All matches were performed to a mass tolerance of ±5 ppm. Predicted testosterone metabolites consist of up to three phase I metabolic transformations of testosterone followed by glucuronylation. "Masses found in screen" indicate if and how many times the accurate mass was detected as a metabolic feature in the metabolomic profiling. A total of 97 detected metabolic features matched the hypothetical structures below. *Indicates a possible metabolite identity according to literature searches.[10–12]

Masses found in Screen	Chemical Formula	Possible/Theoretical structure	Transformation/Name	Theoretical accurate mass (*m/z*)
9	$C_{25}H_{35}O_8^-$	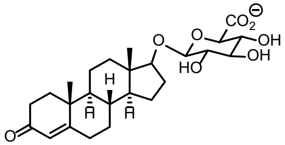	Testosterone glucuronide<br>*epitestosterone glucuronide<br>*dehydroepiandrosterone glucuronide<br>*3α,5-cyclo-6ß-hydroxy-5α-androstan-17-one glucuronide<br>*dehydroandrosterone glucuronide	463.23320
2	$C_{25}H_{33}O_8^-$	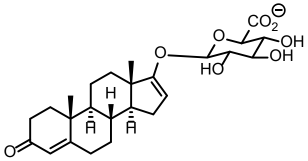	1x reduction<br>*Androstenedione glucuronide	461.21755
7	$C_{25}H_{37}O_8^-$	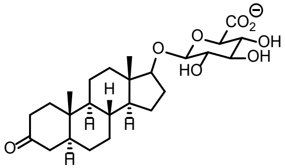	1 x reduction<br>*Androsterone glucuronide<br>*Etiocholanolone glucuronide<br>*Epiandrosterone glucuronide<br>*4-androstene-3ß,17ß-diol glucuronide<br>*17ß-hydroxydehydroepiandrosterone glucuronide<br>*5-androstene-3ß,17ß-diol glucuronide	465.24885
3	$C_{25}H_{39}O_8^-$	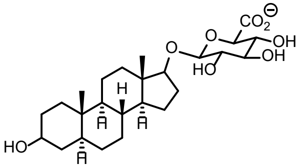	2x reduction<br>*5α-androstane-3α-17ß-diol glucuronide<br>*5ß-androstane-3α-17ß-diol glucuronide<br>*5α-androstane-3ß-17ß-diol glucuronide	467.26450
0	$C_{25}H_{31}O_9^-$	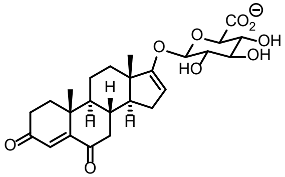	1 x Hydroxylation<br>2 x Oxidation<br>(enolation)	475.19681
3	$C_{25}H_{33}O_9^-$	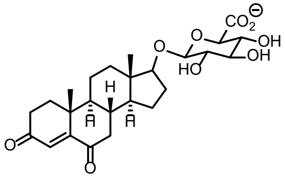	1 x Hydroxylation<br>1 x Oxidation<br>*4-hydroxyandrostenedione glucuronide<br>*6a-hydroxyandrostenedione glucuronide<br>*16α-hydroxyandrostenedione glucuronide<br>*16α-hydroxyandrostenedione glucuronide<br>*7-oxodehydroepiandrosterone glucuronide	477.21246

7	$C_{25}H_{35}O_9^-$	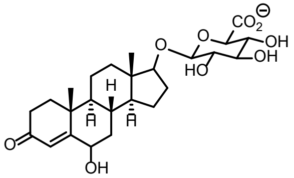	1 x Hydroxylation<br>1 x Reduction<br>1 x Oxidation<br>*7α-hydroxytestosterone glucuronide<br>*4-hydroxytestosterone glucuronide<br>*16α-hydroxydehydroepiandrosterone glucuronide	479.22811
12	$C_{25}H_{37}O_9^-$	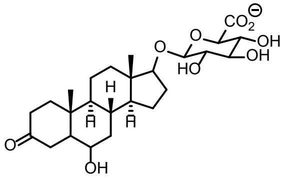	1 x Hydroxylation<br>1 x Reduction<br>*11ß-hydroxyandrosterone glucuronide<br>*11ß-hydroxyetiocholanolone glucuronide<br>*6ß-hydroxyandrosterone glucuronide<br>*6a-hydroxyetiocholanolone glucuronide<br>*7α-hydroxydehydroepiandrosterone glucuronide<br>*11-oxo-5ß-androstane-3α,17ß-diol glucuronide	481.24376
7	$C_{25}H_{37}O_9^-$	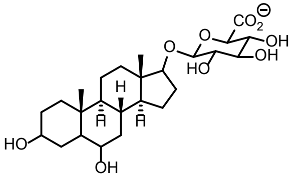	1 x Hydroxylation<br>2 x Reduction	483.25941
2	$C_{25}H_{33}O_{10}^-$	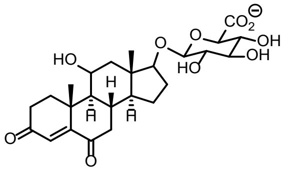	2 x Hydroxylation<br>1 x Oxidation	493.20738
7	$C_{25}H_{35}O_{10}^-$	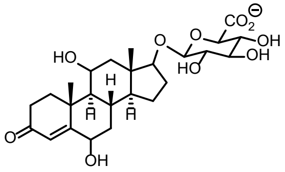	2 x Hydroxylation	495.22303
6	$C_{25}H_{37}O_{10}^-$	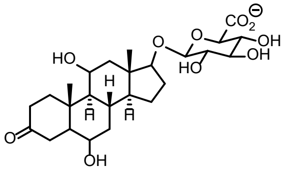	2 x Hydroxylation<br>1 x Reduction	497.23868
3	$C_{25}H_{35}O_{11}^-$	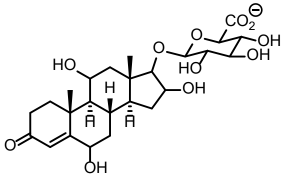	3 x Hydroxylation	511.21794

0	$C_{24}H_{29}O_8^-$	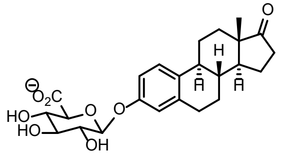	Estrone glucuronide	445.18625
0	$C_{24}H_{31}O_8^-$	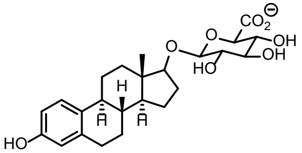	Estradiol glucuronide	447.20190
0	$C_{24}H_{31}O_9^-$	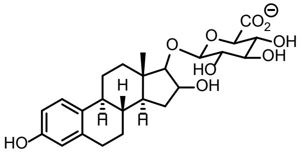	Estratriol glucuronide	463.19681
3	$C_{25}H_{37}O_7^-$	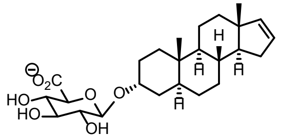	5α-Androst-16-en-3α-ol	449.25393
0	$C_{27}H_{39}O_8^-$	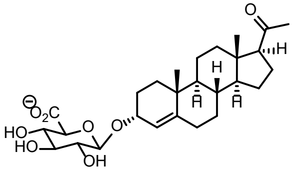	Pregnenolone glucuronide	491.2645
2	$C_{27}H_{43}O_8^-$	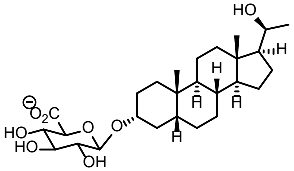	Pregnanediol glucuronide	495.29580
0	$C_{27}H_{37}O_9^-$	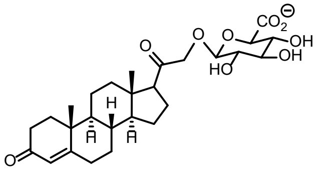	Deoxycorticosterone glucuronide	505.24376
2	$C_{27}H_{39}O_9^-$	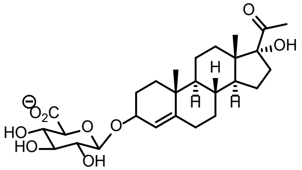	17α-hydroxypregnenolone glucuronide	507.25941

0	$C_{27}H_{37}O_{10}^-$	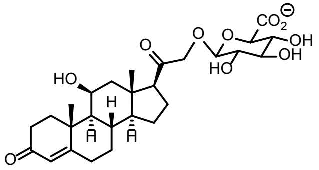	Corticosterone glucuronide<br>*Deoxycoritisol glucuronide	521.23868
9	$C_{27}H_{41}O_{10}^-$	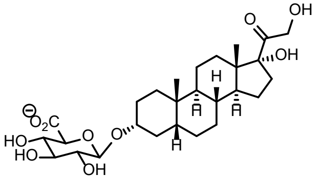	11-Deoxytetrahydrocortisol glucuronide	525.26998
6	$C_{27}H_{37}O_{11}^-$	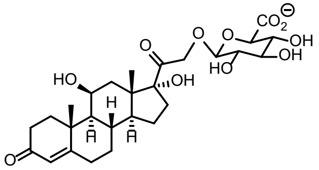	Hydrocortisone glucuronide<br>*Cortisol glucuronide	537.23359
7	$C_{27}H_{41}O_{11}^-$	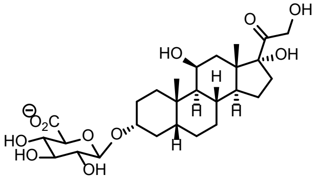	Tetrahydrocortisol glucuronide	541.26489

Table S.9: Masses and ion transitions used to identify glucuronide derived fragments in MS/MS. Based on the open detection for steroid glucuronides method developed by *Fabregat et. al***.**[12] The abbreviation Gluc indicates a dehydrated glucuronic acid ($C_6H_8O_6$).

**Product ion (*m/z*)**	**Formula**	**Nominal mass**	**Accurate mass**
$[HOCH_2CO_2]^-$	$C_2H_3O_3^-$	75	75.0082
$[Gluc\text{-}H\text{-}H_2O\text{-}CO_2\text{-}CO]^-$	$C_4H_5O_2^-$	85	85.029
$[Gluc\text{-}H\text{-}H_2O\text{-}CO_2]^-$	$C_5H_5O_3^-$	113	113.0239
$[Gluc\text{-}H\text{-}H_2O]^-$	$C_6H_5O_5^-$	157	157.0137
$[Gluc\text{-}H]^-$	$C_6H_7O_6^-$	175	175.0242
**Neutral loss (Da)**			
Dehydrated glucuronide (Gluc)	$C_6H_8O_6$	176	176.0321
Glucuronic acid	$C_6H_{10}O_7$	194	194.0427

Table S.10: A list of the 97 putative steroid glucuronide features found in the 1214 significantly changed non-sulfate features in application 2. Matching was performed based on MS and MS/MS transitions. Each molecule was matched against the list of steroid glucuronide structures in Table S.8 and were only considered if they also possessed characteristic MS/MS fragments of steroid glucuronides as seen in table S.9. All matching in both MS and MS/MS was performed to a mass tolerance of ±5 ppm. The $\log_2$ fold change and adjusted p-value are listed for each sulfatase treatment sample in application 2. Of these 91/97 of the putative glucuronides underwent hydrolysis in the HpS treated sample (i.e., had a large negative fold change) relative to the control sample. Significantly changed features (adjusted p-value < 0.01) are highlighted in red.

RT [min]	*m/z*	S/N average	theoretical *m/z* (± 5 ppm)	Log₂FC				Adjusted p-value				Cluster	MS2 transitions (nominal mass)						
													m/z 75	*m/z* 85	*m/z* 113	*m/z* 157	*m/z* 175	176 Da	194 Da
													Species (abundance %)						
				WT	PVFV	LEF	HpS	WT	PVFV	LEF	HpS		$[HOCH_2CO_2]^-$	$[Gluc\text{-}H\text{-}H_2O\text{-}CO_2\text{-}CO]^-$	$[Gluc\text{-}H\text{-}H_2O\text{-}CO_2]^-$	$[Gluc\text{-}H\text{-}H_2O]^-$	$[Gluc\text{-}H]^-$	NL Gluc+H_2O	NL Glucuronide
16.43	449.2551	344	449.2539	0	0	0	-3	1E+00	6E-01	9E-01	3E-09	1	2%	0%	0%	0%	0%	0%	0%
16.12	449.2552	9	449.2539	0	0	0	-8	1E+00	1E+00	9E-01	1E-06	1	33%	21%	1%	0%	0%	0%	0%
13.37	449.2554	19	449.2539	0	0	0	0	8E-01	9E-01	9E-01	2E-02	1	33%	22%	2%	0%	0%	0%	0%
11.04	461.2188	9	461.2176	0	0	0	-9	1E+00	1E+00	1E+00	2E-06	1	20%	17%	2%	0%	0%	0%	0%
11.19	461.2192	9	461.2176	0	0	0	-10	5E-01	6E-01	8E-01	5E-14	1	20%	14%	2%	0%	0%	0%	0%
12.39	463.2345	19	463.2332	0	0	0	-9	1E+00	1E+00	1E+00	5E-06	1	9%	6%	1%	0%	0%	0%	0%
14.40	463.2345	8	463.2332	0	0	0	-5	1E+00	1E+00	1E+00	1E-03	1	24%	19%	4%	0%	0%	0%	0%
11.54	463.2346	123	463.2332	0	0	0	-12	7E-01	1E+00	9E-01	5E-17	1	29%	22%	6%	0%	0%	0%	0%
13.06	463.2347	32	463.2332	0	0	0	-2	1E+00	7E-01	8E-01	2E-06	1	26%	20%	3%	0%	0%	1%	0%
11.98	463.2347	292	463.2332	0	0	0	-5	5E-01	1E+00	1E+00	2E-11	1	29%	15%	3%	0%	0%	0%	0%
12.26	463.2347	70	463.2332	0	0	0	-12	1E-01	9E-01	6E-01	3E-16	1	27%	16%	4%	0%	0%	0%	0%
11.18	463.2348	38	463.2332	0	0	0	-11	1E+00	5E-01	5E-01	2E-13	1	21%	20%	2%	0%	0%	0%	0%
14.58	463.2353	4	463.2332	0	0	0	-3	8E-01	1E+00	8E-01	3E-07	1	14%	10%	1%	0%	0%	0%	0%
10.88	463.2355	30	463.2332	0	0	0	-10	2E-01	8E-01	2E-02	4E-15	1	18%	14%	3%	0%	0%	1%	0%
10.36	465.2501	15	465.2489	0	0	0	-11	5E-01	1E+00	9E-01	2E-14	1	30%	22%	1%	0%	0%	0%	0%
13.81	465.2502	56	465.2489	0	0	0	-11	1E+00	1E+00	1E+00	5E-06	1	33%	19%	0%	0%	0%	0%	0%
12.59	465.2502	729	465.2489	0	0	0	-12	1E+00	1E+00	9E-01	2E-10	1	32%	22%	5%	0%	0%	0%	0%
11.65	465.2502	94	465.2489	0	0	0	-14	6E-01	7E-01	1E+00	5E-17	1	30%	19%	0%	0%	0%	0%	0%
12.80	465.2503	1160	465.2489	0	0	0	-6	6E-01	1E+00	9E-01	3E-11	1	32%	21%	3%	0%	0%	0%	0%
13.56	465.2505	75	465.2489	0	0	0	-5	9E-01	1E+00	1E+00	8E-13	1	32%	22%	2%	0%	0%	0%	0%

11.99	465.2508	30	465.2489	0	0	0	-12	5E-01	7E-01	1E+00	8E-15	1	16%	14%	1%	0%	0%	0%	0%
14.19	467.2657	39	467.2645	0	0	0	-11	6E-01	7E-01	3E-01	8E-14	1	33%	22%	4%	0%	0%	0%	0%
13.05	467.2659	131	467.2645	0	0	0	-13	1E+00	1E+00	8E-01	2E-15	1	36%	28%	3%	0%	0%	0%	0%
12.41	467.2662	33	467.2645	0	0	0	-9	1E+00	1E+00	1E+00	2E-06	1	25%	19%	2%	0%	0%	0%	0%
10.28	477.2138	26	477.2125	0	0	0	-11	2E-01	6E-01	5E-01	5E-16	1	26%	21%	5%	0%	0%	0%	0%
9.07	477.2144	14	477.2125	0	0	0	-10	3E-01	3E-01	4E-01	9E-15	1	10%	13%	5%	0%	0%	0%	0%
9.28	477.2148	9	477.2125	0	0	0	-9	1E+00	1E+00	5E-01	3E-12	1	23%	22%	3%	0%	0%	0%	0%
13.41	479.2273	31	479.2281	0	0	0	3	4E-01	4E-01	5E-01	3E-08	1	6%	6%	0%	0%	0%	0%	0%
8.51	479.2293	7	479.2281	0	0	0	-11	1E+00	1E+00	1E+00	2E-14	1	19%	23%	6%	0%	0%	0%	0%
8.63	479.2294	7	479.2281	-1	0	-1	-3	3E-01	5E-01	4E-01	9E-06	1	17%	16%	5%	0%	0%	0%	0%
10.27	479.2294	108	479.2281	0	0	0	-15	4E-01	8E-01	8E-01	9E-17	1	30%	22%	6%	0%	0%	3%	1%
11.10	479.2295	28	479.2281	0	0	0	-13	7E-03	6E-02	6E-01	5E-17	1	30%	12%	2%	0%	0%	0%	0%
10.41	479.2295	21	479.2281	0	0	0	-12	4E-01	9E-01	9E-01	2E-14	1	27%	21%	5%	0%	0%	0%	0%
9.67	479.2297	79	479.2281	0	0	0	-13	1E+00	1E+00	1E+00	5E-08	1	29%	22%	4%	0%	0%	0%	0%
11.44	481.2448	6	481.2438	0	0	0	-5	8E-01	1E+00	1E+00	1E-07	1	19%	21%	5%	0%	0%	0%	0%
6.32	481.2449	5	481.2438	-1	0	0	-8	7E-01	1E+00	1E+00	4E-07	1	33%	19%	4%	0%	0%	0%	0%
9.85	481.2449	315	481.2438	0	0	0	-4	2E-01	1E+00	8E-01	7E-11	1	27%	25%	2%	0%	0%	0%	0%
11.31	481.2450	8	481.2438	0	0	0	-11	8E-01	1E+00	6E-01	3E-15	1	22%	20%	4%	0%	0%	0%	0%
8.81	481.2451	18	481.2438	0	0	0	-3	3E-01	7E-01	7E-01	4E-07	1	30%	28%	5%	0%	0%	0%	0%
10.53	481.2451	5	481.2438	0	0	0	-6	6E-01	1E+00	1E+00	6E-11	1	16%	17%	3%	0%	0%	0%	0%
11.88	481.2451	5	481.2438	0	0	0	-7	1E+00	1E+00	1E+00	2E-05	1	20%	12%	4%	0%	0%	0%	0%
8.31	481.2451	14	481.2438	0	0	0	-11	2E-01	3E-01	3E-01	2E-15	1	30%	23%	5%	0%	0%	0%	0%
7.01	481.2452	5	481.2438	0	0	0	-10	5E-01	5E-01	9E-01	2E-13	1	26%	23%	4%	0%	0%	0%	0%
11.19	481.2452	14	481.2438	0	0	0	-10	8E-01	8E-01	8E-01	1E-15	1	22%	19%	4%	0%	0%	0%	1%
8.96	481.2452	18	481.2438	0	0	0	-11	1E+00	1E+00	1E+00	9E-08	1	27%	22%	3%	0%	0%	0%	0%
9.63	481.2454	86	481.2438	0	0	0	-14	2E-01	1E+00	6E-01	3E-17	1	29%	24%	4%	0%	0%	0%	0%
12.56	483.2603	7	483.2594	0	0	0	-9	4E-01	2E-01	1E+00	1E-14	1	26%	20%	5%	0%	0%	3%	0%
10.94	483.2606	47	483.2594	0	0	0	-9	1E+00	1E+00	1E+00	6E-05	1	26%	22%	5%	0%	0%	0%	0%
12.20	483.2606	12	483.2594	0	0	0	-10	6E-01	6E-01	4E-01	1E-15	1	24%	23%	5%	0%	0%	0%	0%
12.42	483.2609	26	483.2594	0	0	0	-11	8E-01	1E+00	8E-01	1E-14	1	25%	28%	6%	0%	0%	0%	0%
12.09	483.2610	19	483.2594	1	1	1	-10	1E-01	5E-02	6E-02	2E-12	1	25%	27%	6%	0%	0%	0%	0%
10.72	483.2610	18	483.2594	0	0	0	-3	6E-01	1E+00	9E-01	4E-09	1	25%	23%	5%	0%	0%	0%	0%
11.05	483.2611	36	483.2594	0	0	0	-9	1E+00	1E+00	1E+00	6E-06	1	29%	23%	3%	0%	0%	0%	0%
7.91	493.2084	13	493.2074	-1	0	0	-9	3E-02	2E-01	3E-01	8E-13	1	5%	15%	4%	0%	0%	1%	0%
11.74	493.2090	6	493.2074	0	0	0	-9	8E-01	5E-01	4E-01	3E-10	1	8%	12%	3%	0%	0%	0%	0%

7.68	495.2241	159	495.2230	2	-9	-7	-10	1E+00	3E-01	3E-01	1E-01	1	8%	11%	9%	0%	0%	0%	0%
7.73	495.2241	332	495.2230	0	-10	-10	-7	1E+00	1E-01	1E-01	2E-01	1	7%	16%	11%	0%	0%	0%	0%
7.33	495.2243	110	495.2230	0	0	0	-12	9E-01	1E+00	1E+00	4E-07	1	5%	17%	5%	0%	0%	9%	0%
7.84	495.2243	328	495.2230	0	0	0	-14	1E+00	1E+00	1E+00	4E-10	1	5%	14%	5%	0%	0%	17%	0%
7.62	495.2243	330	495.2230	2	7	7	-3	9E-01	5E-01	4E-01	7E-01	1	8%	14%	8%	0%	0%	0%	0%
8.08	495.2244	6	495.2230	0	0	0	-5	7E-01	1E+00	1E+00	8E-08	1	7%	12%	4%	0%	0%	1%	0%
10.76	495.2244	21	495.2230	0	0	0	-5	7E-01	6E-01	1E+00	2E-11	1	6%	10%	1%	0%	0%	0%	0%
14.43	495.2968	254	495.2958	0	0	0	-15	1E+00	7E-01	9E-01	5E-17	1	35%	23%	2%	0%	0%	0%	0%
14.29	495.2971	34	495.2958	0	0	0	-11	4E-01	8E-01	6E-02	5E-17	1	24%	25%	2%	0%	0%	0%	0%
5.86	497.2396	6	497.2387	0	0	0	-9	3E-01	1E+00	5E-01	8E-12	1	21%	21%	2%	0%	0%	0%	0%
10.94	497.2397	7	497.2387	0	0	0	-7	1E+00	1E+00	1E+00	4E-04	1	7%	16%	1%	0%	0%	0%	0%
10.33	497.2400	26	497.2387	0	0	0	-10	1E+00	1E+00	9E-01	1E-06	1	28%	26%	5%	0%	0%	0%	0%
8.91	497.2401	6	497.2387	-1	0	0	-9	2E-01	5E-01	3E-01	3E-11	1	11%	15%	9%	0%	0%	0%	0%
8.08	497.2401	44	497.2387	0	0	0	-12	4E-01	1E+00	8E-01	6E-15	1	5%	14%	3%	0%	0%	18%	0%
10.47	497.2403	16	497.2387	0	0	0	-7	9E-01	1E+00	1E+00	3E-05	1	23%	23%	5%	0%	0%	0%	0%
12.54	507.2599	7	507.2594	0	0	0	-9	8E-01	9E-01	5E-01	3E-13	1	6%	15%	4%	0%	0%	0%	0%
10.79	507.2604	7	507.2594	0	0	0	-9	7E-01	4E-01	8E-01	1E-14	1	12%	17%	6%	0%	0%	0%	0%
5.73	511.2188	8	511.2179	-1	0	0	-10	1E-01	1E+00	9E-01	6E-13	1	2%	16%	0%	0%	0%	13%	0%
12.18	511.2193	9	511.2179	0	0	1	-1	7E-01	9E-01	2E-01	1E-01	1	5%	36%	8%	0%	0%	0%	0%
5.12	511.2194	7	511.2179	0	0	0	-9	6E-01	8E-01	6E-01	7E-12	1	4%	12%	1%	0%	0%	6%	0%
13.17	525.2707	22	525.2700	0	0	0	-11	1E+00	9E-01	1E+00	3E-16	1	18%	19%	6%	0%	0%	0%	0%
9.91	525.2708	192	525.2700	0	0	0	-2	9E-01	6E-01	8E-01	7E-08	1	37%	23%	0%	0%	0%	0%	0%
12.66	525.2709	6	525.2700	0	0	0	-10	4E-01	3E-01	8E-01	3E-14	1	28%	21%	7%	0%	0%	0%	0%
12.13	525.2709	20	525.2700	0	0	0	-11	1E+00	6E-01	7E-01	6E-15	1	29%	20%	5%	0%	0%	0%	0%
11.67	525.2709	24	525.2700	0	0	0	-12	9E-01	9E-01	1E+00	4E-15	1	25%	22%	6%	0%	0%	0%	0%
11.77	525.2709	46	525.2700	0	0	0	-13	2E-01	3E-01	1E+00	3E-16	1	22%	20%	7%	0%	0%	0%	0%
10.83	525.2712	18	525.2700	0	0	0	-11	6E-01	1E+00	5E-01	2E-15	1	28%	19%	6%	0%	0%	0%	0%
10.35	525.2714	14	525.2700	0	0	0	-9	9E-01	1E+00	1E+00	7E-06	1	24%	21%	3%	0%	0%	0%	0%
11.06	525.2716	16	525.2700	0	0	0	-9	1E+00	1E+00	1E+00	8E-07	1	20%	21%	8%	0%	0%	0%	0%
10.97	537.2340	13	537.2336	0	0	0	-10	2E-01	5E-01	8E-01	3E-16	1	15%	15%	6%	0%	0%	0%	0%
10.09	537.2343	52	537.2336	0	0	0	-12	4E-01	8E-01	8E-01	3E-16	1	25%	17%	6%	0%	0%	0%	0%
10.00	537.2344	25	537.2336	0	0	0	-11	9E-02	1E+00	9E-01	3E-16	1	27%	18%	6%	0%	0%	0%	0%
9.20	537.2347	23	537.2336	0	0	0	-11	5E-01	9E-01	7E-01	9E-14	1	14%	17%	5%	0%	0%	0%	0%
10.62	537.2347	24	537.2336	0	0	0	-11	5E-01	7E-01	5E-01	4E-14	1	19%	20%	8%	0%	0%	0%	0%
8.42	537.2349	9	537.2336	0	0	0	-9	1E-01	9E-01	6E-01	7E-15	1	20%	27%	16%	0%	0%	0%	0%

9.32	541.2656	2836	541.2649	0	0	0	-2	4E-02	4E-01	7E-01	8E-09	1	26%	24%	3%	0%	0%	0%	0%
10.73	541.2656	299	541.2649	0	0	0	-13	1E+00	4E-01	5E-01	7E-15	1	11%	18%	7%	0%	0%	0%	0%
9.93	541.2656	1017	541.2649	0	0	0	-15	8E-01	1E+00	1E+00	1E-14	1	30%	21%	10%	0%	0%	0%	0%
9.65	541.2658	328	541.2649	0	0	0	-9	7E-01	1E+00	7E-01	4E-12	1	34%	21%	9%	0%	0%	0%	0%
11.22	541.2658	3228	541.2649	0	0	0	-13	1E+00	1E+00	1E+00	1E-06	1	16%	21%	7%	0%	0%	0%	0%
7.78	541.2659	5	541.2649	-1	0	0	-3	1E-01	1E+00	5E-01	1E-05	1	26%	20%	2%	0%	0%	0%	0%
8.10	541.2659	21	541.2649	-1	0	0	-3	1E-01	5E-01	8E-01	3E-07	1	21%	15%	3%	0%	0%	0%	0%

Table S.11: Concentrations of 33 free steroids detected in pooled human urine samples, after incubation with or without (control) deconjugating enzymes (ng/mL, mean ± SEM, n=3). Significance denoted on two levels (*p<0.05, ** p<0.01).

Steroid	Control	± SEM	WT-PaS	± SEM	PVFV-PaS	± SEM	LEF-PaS	± SEM	E. coli β-gluc	± SEM
testosterone	0.6	0.1	0.74	0.08	1.45**	0.06	1.48**	0.08	10.23**	0.26
epitestosterone	0.2	0.0	0.22	0.01	0.29	0.02	0.25	0.04	8.89**	0.26
androsterone	113.7	2.3	79.65	3.49	80.99	1.79	128.75	46.52	1282.17**	5.67
etiocholanolone	14.0	0.7	8.87*	0.31	8.44*	0.22	12.19	3.47	739.48**	6.96
5α-androstane-3α,17ß-diol	1.5	0.2	1.32	0.04	1.17	0.06	1.52	0.47	34.90**	0.22
5ß-androstane-3α,17ß-diol	1.3	0.2	0.81**	0.05	0.80**	0.07	1.14	0.11	51.34**	0.72
16α-hydroxydehydroepiandrosterone	2.8	0.1	485.21**	59.96	836.39**	76.80	639.71**	52.83	9.97**	0.95
dehydroepiandrosterone	3.6	0.1	658.81**	45.76	916.04**	9.59	705.48**	7.50	10.52**	0.91
androst-5-ene-3ß,17ß-diol	0.9	0.2	121.73**	7.85	192.03**	1.29	185.62**	1.90	1.71**	0.09
4-hydroxytestosterone	0.0	0.0	10.37**	1.27	17.43**	1.68	13.49**	1.23	0.05	0.02
epiandrosterone	0.1	0.0	69.02**	1.50	73.76**	0.95	69.81**	0.33	1.17**	0.10
7α-hydroxydehydroepiandrosterone	0.3	0.1	20.49**	2.45	42.19**	5.32	15.15**	1.19	1.76**	0.14
16α-hydroxyandrostenedione	0.2	0.0	9.08**	0.46	4.44**	0.43	7.84**	0.49	4.94**	0.43
5α-androstane-3ß,17ß-diol	0.3	0.1	3.00*	0.06	7.47**	0.23	7.67**	0.26	0.56	0.30
4-hydroxyandrostenedione	0.0	0.0	0.07	0.02	0.08	0.02	0.06	0.01	0.84**	0.07
5α-androst-16-en-3α-ol	0.3	0.0	0.23	0.02	0.31	0.01	0.47	0.12	5.31**	1.96
androstenedione	3.1	1.2	1.79	0.22	3.39	0.19	1.70	0.04	52.59**	3.18
tetrahydrocortisol	10.0	0.9	10.11	0.36	10.18	0.97	9.66	1.16	675.83**	64.47
pregnanediol	4.2	0.2	2.77*	0.25	3.08	0.19	4.31	0.84	151.76**	2.18
6α-hydroxyandrostenedione	0.1	0.0	0.11	0.01	0.18**	0.03	0.14*	0.04	0.64**	0.06
7α-hydroxytestosterone	0.5	0.2	1.73	1.46	1.49	1.21	1.48	1.16	3.52	2.01
7ß-hydroxydehydroepiandrosterone	0.8	0.5	2.09**	0.16	2.09**	0.05	1.50*	0.13	15.30**	0.44
11ß-hydroxyandrosterone	0.7	0.0	0.46	0.06	0.28	0.16	0.60	0.22	338.32**	20.47
11ß-hydroxyetiocholanolone	0.4	0.1	0.27	0.15	0.21	0.05	0.32	0.17	68.45**	5.33
7-oxo-dehydroepiandrosterone	0.2	0.0	0.12	0.04	0.12	0.04	0.07*	0.02	20.22**	0.55
11-oxo-5ß-androstane-3α,17ß-diol	0.1	0.0	0.04	0.02	0.05	0.01	0.03	0.03	7.89**	0.29
androst-5-en-3α-ol-17-one	2.1	0.2	0.37**	0.05	0.31**	0.05	0.60**	0.13	63.91**	4.09
16α-hydroxyandrostenedione	0.3	0.1	0.52*	0.02	0.71**	0.05	0.56**	0.10	66.87**	7.16
11-deoxytetrahydrocortisol	0.4	0.1	0.84**	0.07	1.14**	0.05	1.00**	0.24	38.23**	2.70
6ß-hydroxyandrosterone	0.0	0.0	0.05	0.01	0.08	0.03	0.03	0.02	2.75**	0.18
6α-hydroxyetiocholanolone	0.1	0.0	0.21	0.09	0.28	0.11	0.17	0.06	182.46**	11.07
cortisol	15.6	1.1	26.74*	1.80	23.74*	3.74	21.79	2.08	28.17**	2.63
3α,5-cyclo-6ß-hydroxy-5α-androstan-17-one	36.7	0.8	21.92**	1.32	14.65**	1.73	20.81**	0.94	19.94**	1.75

Table S.12: Displays matches of *m/z* (±5 ppm) between the sulfated masses of reference molecules with high sulfatase activity with putative candidates found from the untargeted metabolic profile in the pooled human urine enzyme hydrolysis study. 'Potential candidate detected'; *Yes,* indicates that within that metabolome a molecule with the correct *m/z* (±5 ppm) was found to have matched the sulfated mass of the reference molecule that had undergone significant and largely negative change (i.e., adjusted p value < 0.01 and a negative fold change), *No* indicates that the molecule was found not to have a significant change.

		**Potential candidate detected**		
**Name of metabolite**	**Sulfated mass (m/z)**	**WT-pas**	**PVFV-pas**	**LEF-pas**
testosterone	367.1589	Yes	Yes	Yes
epitestosterone	367.1589	Yes	Yes	Yes
androsterone	369.1749	Yes	Yes	Yes
etiocholanolone	369.1749	Yes	Yes	Yes
5α-androstane-3α,17ß-diol	371.1892	Yes	Yes	No
5ß-androstane-3α,17ß-diol	371.1892	Yes	Yes	No
16α-hydroxydehydroepiandrosterone	383.1529	Yes	Yes	Yes
dehydroepiandrosterone	367.1589	Yes	Yes	Yes
androst-5-ene-3ß,17ß-diol	369.1749	Yes	Yes	Yes
4-hydroxytestosterone	383.1529	Yes	Yes	Yes
7α-hydroxydehydroepiandrosterone	383.1529	Yes	Yes	Yes
16α-hydroxyandrostenedione	381.13719	Yes	Yes	No
5α-androstane-3ß,17ß-diol	371.1892	Yes	Yes	No

Table S.13: The results for metabolic profiling of epiandrosterone sulfate in each enzyme treatment in application 2. Data for the stable isotope labelled internal standard (SIL-IS) epiandrosterone [$^{18}O_3$]-sulfate (**S3**) is also shown for the WT-*PaS* treated sample. Log_2 fold change (Log_2FC), adjusted p-value (adj. p-value), and retention time (RT).

Enzyme treatment	Log_2(FC)	adj. p-value	RT (min)	*m/z*
Wild Type	-14	7.5E-17	12.49	369.1749
SIL-IS* (**S3**)	0.1	0.9	12.49	375.1872
PVFV	-14	7.6E-17	12.49	369.1749
LEF	-14	7.5E-17	12.49	369.1749
HpS	-1	9.9E-07	12.49	369.1749

*The SIL-IS epiandrosterone sulfate was added post hydrolysis

S.3 Additional Methodology Details

Alignment and normalisation of data using MS-DIAL

In this work MS-DIAL was used for the alignment of the triplicate sample files and QC samples. All data was then normalised against pooled QC samples using the built-in locally weighted scatterplot smoothing (LOWESS) function.[13,14] This normalised list was then exported as a text file (.txt).

Script based alignment of MS and DDA MS/MS data

The *MS*/MS product ion spectra were aligned to the normalised list using two separate python scripts. The first script uses the 'natsort' function in python to extract both *MS* and *MS/MS* data from the raw data files as centroid mascot generic file (.mgf) format. Note: '.raw' files were converted into .mgf format using the MSConvert program from ProteoWizard.[15] For each identified peak six sulfate product ions are explicitly reported as both raw and normalised intensities. Note; if the product ion spectra do not have one of these peaks the abundance is reported as zero. The second script aligns all measured *MS/MS* data against the list of normalised MS data from MS-DIAL, this was done with a mass tolerance of ±5.0 ppm and retention time tolerance of ±3 seconds. During this process two new columns were added to the data called Intensity Ratio (IR) and Maximum Abundance (MA). Resulting in a single comma delimited '.cvs' formatted table containing all aligned molecular features with their associated MS/MS data.

The IR, equation S.3.1, is equal to the summed intensity of all sulfate reporter ions, divided by the sum of all product ions (minus the precursor intensity), in the product ion spectra for a given molecule detected in first in MS then in MS/MS. Maximum abundance (MA) is defined as the intensity of the sulfate reporter ion (according to manuscript Table 1) with the highest relative abundance in the product ion spectra for each molecule detected in MS/MS. As the fragmentation of sulfate metabolites is typically dominated by sulfate derived product ions or neutral losses, IR and MA were used to give a ready measure of sulfate-like behaviour.

Equation S.3.1

$$IR = \frac{\sum Sulfate\ reporter\ ion\ count}{\sum All\ product\ ion\ count}$$

Unsupervised *k*-means clustering algorithm in R

Following the alignment steps, data undergoes unsupervised clustering, followed by a high throughput differential metabolite level analysis, using R code. *k-mean*s clustering algorithm is used to determine 'sulfate-like behaviour'. This is done by sorting normalised data into two clusters, the data used to do so was the relative abundance of six different reporter ions in the product ion spectra, and the two filtering parameters IR and MA, defined above.[3] The cluster with a higher proportion of sulfate like features was defined as 'sulfate' and that with a lower proportion was defined as 'non-sulfate'.

High throughput differential metabolite level analysis in R

High throughput differential metabolite level analysis of the data was performed in R, using the Bioconductor's *Limma* and Glimma packages.[16,17] It should be noted that both packages were originally developed for genomics micro array data, from which the t-test is moderated [weighted] across all samples giving rise to an adjusted p-value as the output, reducing the need for *ad hoc* decisions about which observations need to be filtered out, or for ones that are false positives.[17] The *toptable()* function gives a summary of basic statistical analysis,

these include, log_2 fold change, standard errors, *t-statstics,* and p-values and adjusted p-values. To create the volcano plots log_2 fold change and adjusted p-value was used. Adjusted p-values were used as they account for false discovery rates in the data.[16] Volcano plots are then generated as visual aids to identify significantly changed metabolomic features. Note: all scripts are available in Section S.7.

Example output of data

Table S.14: Example of data output for putative steroid sulfate metabolites identified from equine urine after administration of testosterone propionate in application 1. This consists of aligned, normalised, and tabulated list of detected metabolic features with appended UHPLC (retention time), HRMS (*m/z,* S/N, charge and possible adduct type), differential metabolite level statistics (p value, adjusted p value, log_2 fold change), *k-means* clustering results (sulfate or non-sulfate), and associated normalised intensity of sulfate product ions from the product ion spectra. Cluster indicates grouping after *k-means* sorting was applied, either identified as non-sulfate (1) or sulfate (2). This output table is divided over three tables to aid readability.

Row id	RT [min]	m/z	z	Adduct type	MS/MS assigned	S/N average	Ratio	Max Count	Precursor Intensity	$.SO_3^-$	HSO_3^-	$.SO_4^-$	HSO_4^-	Precursor	Neutral Loss SO_3	Neutral Loss H_2SO_4	Total Intensity	cluster	Theoretical m/z
3557	8.772	367.1583	-1	[M-H]-	TRUE	137	78	100	8E+06	9	1	7	100	26	0	0	2E+06	2	367.1583
3604	7.690	369.1739	-1	[M-H]-	TRUE	11	70	100	1E+06	12	5	1	100	47	0	0	6E+05	2	369.1739
3605	10.164	369.1739	-1	[M-H]-	TRUE	26	78	95	2E+06	3	1	0	95	5	0	0	1E+06	2	369.1739
3638	10.534	371.1895	-1	[M-H]-	TRUE	3	87	100	6E+05	4	0	0	100	17	0	0	3E+05	2	371.1895
3919	6.374	383.1537	-1	[M-H]-	TRUE	14	62	100	2E+06	6	0	0	100	3	0	0	1E+06	2	383.1528
3965	6.742	385.1690	-1	[M-H]-	TRUE	3.5	87	85	1E+06	55	45	0	85	4	0	0	6E+05	2	385.1685
4009	7.594	387.1845	-1	[M-H]-	TRUE	14	91	100	3E+06	6	4	0	100	10	0	0	1E+06	2	387.1841
4010	7.388	387.1846	-1	[M-H]-	TRUE	9	89	100	4E+06	3	0	0	100	6	0	0	1E+06	2	387.1841
4014	4.564	387.1846	-1	[M-H]-	TRUE	9	82	100	2E+06	2	0	0	100	7	0	0	1E+06	2	387.1841
4016	4.475	387.1849	-1	[M-H]-	TRUE	9	78	100	1E+06	2	0	0	100	7	0	0	8E+05	2	387.1841

…

Horse 1_Plus 12 hours					Horse 2_T1					
log_2 FC	t	p-value	adj. p-value	B	log_2 FC	Average expression	t	p-value	adj. p-value	B
10	15	7E-09	2E-07	10	-2	16	-3	1E-02	3E-02	-3
9	11	3E-07	6E-06	7	-1	12	-2	1E-01	2E-01	-5
6	11	3E-07	5E-06	7	-2	17	-3	2E-02	4E-02	-4
8	9	1E-06	2E-05	6	-1	13	-2	1E-01	2E-01	-5
7	5	2E-04	1E-03	1	-1	13	-1	4E-01	5E-01	-6
4	17	2E-09	7E-08	11	0	19	1	1	1	-6
10	9	1E-06	2E-05	6	-3	13	-3	2E-02	4E-02	-4
8	4	2E-03	8E-03	-1	0	12	0	1E+00	1E+00	-7
9	8	4E-06	5E-05	5	-3	13	-3	8E-03	2E-02	-3
9	9	2E-06	2E-05	6	-3	13	-3	7E-03	2E-02	-3

…

Horse 2_T2						Horse 2_T3					
log_2 FC	Average expression	t	p-value	adj. p-value	B	log_2 FC	Average expression	t	p-value	adj. p-value	B
-1	16	-2	1E-01	2E-01	-5	0	16	0	8E-01	9E-01	-7
-1	12	-2	1E-01	2E-01	-5	-1	12	-2	1E-01	2E-01	-5
-1	17	-1	2E-01	3E-01	-6	-1	17	-1	2E-01	4E-01	-6
-1	13	-1	5E-01	6E-01	-6	1	13	1	5E-01	6E-01	-6
-2	13	-2	1E-01	2E-01	-5	-3	13	-2	3E-02	6E-02	-4
0	19	1	1	1	-6	1	19	3	2E-02	4E-02	-4
-1	13	-1	2E-01	3E-01	-6	-3	13	-3	2E-02	4E-02	-4
1	12	1	6E-01	7E-01	-7	1	12	0	7E-01	8E-01	-7
-3	13	-3	8E-03	2E-02	-3	-3	13	-3	8E-03	2E-02	-3
-3	13	-3	7E-03	2E-02	-3	-3	13	-3	7E-03	2E-02	-3

Validation

A two-fold validation was used. The main validation for this untargeted assay was the use of PCA plots, as tight clustering of pooled QC samples and randomised triplicate samples indicated good data quality (Figures S.1& S.2).[2,18–20] Common criteria for targeted assays was also used to assess the methods. However, this is not necessarily required for this type of profiling analysis. This included checking chromatographic reproducibility RSD%, sample carryover, response linearity, and limit of detection (LOD) estimation for a small number of model steroid sulfates on separate days to the analysis (Table S.15).[21] The extraction protocol was assessed through extraction recovery experiments carried out during, and on separate days, to the analysis.

Table S.15: Validation results for UHPLC-HRMS/MS analytical methods for the untargeted detection of sulfate metabolites in human and equine urine, application 1 and 2.

Analyte	Chemical formula	Precursor ion $[M\text{-}H]^-$	Experiment[a]	t_R (min) (%RSD)[b]	LOD (ng/mL)[c]	Linear regression equation	Correlation coefficient (R^2)	Extraction efficiency (%)[d]
nandrolone sulfate (**S1**)	$[C_{18}H_{25}O_5S]^-$	353.1414	HUS	11.05 (0.055)				102
			EUS	8.89 (1.26)				
cholanediol bis(sulfate) (**S2**)	$[C_{24}H_{41}O_8S_2]^{2-}$	260.1088	HUS	14.49 (0.029)				97
epiandrosterone 3{^{18}O}$_3$ sulfate (**S3**)	$[C_{19}H_{29}\{^{18}O\}_3O_2S]^-$	375.1863	EUS	11.20 (1.01)				
epiandrosterone 3{^{18}O}$_3$ sulfate (**S3**)	$[C_{19}H_{29}\{^{18}O\}_3O_2S]^-$	375.1863	HUS	12.47 (0.05)	7	y = 0.0022x – 0.0057	0.985	
5α-androstane-3β,17β[$^{18}O_3$]-diol bis(sulfate) (**S4**)	$[C_{19}H_{30}[^{18}O_3]O_5S_2]^{2-}$	228.0762	HUS	9.41 (0.08)	3	y = 0.0023x – 0.0017	0.998	
testosterone sulfate (**1**)	$[C_{19}H_{27}O_5S]^-$	375.1863	EUS	9.76 (1.13)	9	y = 0.0046x + 0.0099	0.9998	
epiandrosterone sulfate (**2**)	$[C_{19}H_{29}O_5S]^-$	369.1730	EUS	11.20 (1.01)	4	y = 0.0068x + 0.0101	0.999	

[a]HUS denote the human urine sulfatase screen, application 2, EUS denotes the equine urine screen, application 1.

[b]Retention time [n = 15 for HUS, n = 28 for EUS, (% relative standard deviation)]

[c]Limits of detection (LOD = 3 σ/b) were estimated according to the calibration curves of individual analyte samples as response ratios, where σ is the standard deviation of the regression, b is the slope of the calibration curve. The LOD were performed in stripped urine, at concentration levels of 0, 10, 50, 100, 500 ng/mL for EUS samples and 0, 5, 10, 20, 50 ng/mL for HUS samples.

[d]Extraction recovery (% peak area pre-spike/ peak area post-spike, n = 3) was assessed by pre- and post-spiking of internal standards at 300 ng/mL compared to post-spiking of paired monosulfate and bis(sulfate) internal standards.

Extraction recovery and LOD for UHPLC-HRMS studies

Extraction recovery was calculated by comparing the normalised response ratios of the four internal standards added before extraction and after the extraction and were performed in triplicate at a concentration of 300 ng/mL. To estimate the limit of detection (LOD), reference materials at increasing concentrations (0, 5, 10, 20, 50 ng/mL) were extracted and analysed as previously described, with the responses modelled by linear regression. The limit of detection was calculated from LOD = 3S/b, where S is the standard deviation of the regression, and b is the slope of the calibration curve.[22] Results presented in Table S.15.

Equine urine sample preparation for reference standards

The method has been reported previously.[23] For reference standard preparation, stripped surrogate matrix was used, which comprised of the flow through fraction of un-doped blank urine matrix passed through a C18 cartridge.

General procedure for urine sample preparation for reference standards

An aliquot of urine or surrogate matrix (0.6 mL) was mixed with phosphate buffer (0.3 mL, 100 mM, pH 5.4) and centrifuged (1100 x *g*, 5 min) to pellet solids. The supernatant (0.750 mL) was fortified with a mixture of internal standards (150 µL, 1000 ng/mL, of nandrolone sulfate (**S1**), cholanediol bis(sulfate) (**S2**), epiandrosterone [$^{18}O_3$]-sulfate (**S3**) and 5α-androstane-3β,17β-diol 3,[$^{18}O_3$]17-bis(sulfate) (**S4**) and/or reference materials (150 µL, 1000 ng/mL). Giving a final concentration equivalent to 300 ng/mL original urine volume (0.5 mL) per sample. The supernatant was then loaded onto a Waters™ Oasis WAX SPE cartridge (3cc), that was pre-conditioned with methanol (2 mL) and water (2 mL). Samples were washed with NaOH (2 mL, 0.1 M), phosphate buffer (2 mL, 100mM, pH 7.4), and Milli-Q water (2 mL) before elution with a mixture of ethyl acetate: methanol: diethyl amine (25:25:1 v/v/v, 3 mL) into clean 10 mL glass tubes. All samples were then evaporated to dryness under a reduced pressure at 40 °C and stored at -20 °C until analysis. For analysis, the dry samples were re-dissolved with ACN: water (100 µL, 20% v/v) and filtered using 0.2 µm spin filters and stored at 5 °C until analysis. This resulted in a sample that was approximately 5 times the concentration of the original urine sample (0.5 mL). Results are presented in Tables 2 and S.6.

Targeted GC-MS analysis of free steroids

Materials and reagents

β-Glucuronidase enzyme from *Escherichia coli* K12 was obtained from Roche Diagnostics (Mannheim, Germany). 3M Empore C18 disk solid phase extraction cartridges (Part No. 1214-4002) were purchased from Agilent Technologies Australia (Mulgrave, Australia). *N*-Methyl-*N*-trimethylsilyltrifluoroacetamide (MSTFA) was obtained from Chem. Fabrik Karl Bucher (Waldstetten, Germany).

Gas chromatography tandem mass spectrometry analysis

Steroid profiling was performed by gas chromatography tandem mass spectrometry utilising a 7890B GC coupled to a 7000C MS/MS from Agilent Technologies Australia (Mulgrave, Australia). The GC column was a J&W HP-Ultra 1 (0.2 mm x 0.11 µm x 25 m) from Agilent Technologies Australia. The injection volume was 2.5 µL (15:1 split) at 250 ˚C with an initial pressure of 26 psi (helium carrier gas 1.2 mL/min constant flow). The column temperature was programmed from 115 °C with a hold for 0.8 min, then to 180 °C at 90 °C/min, then to 190 °C at 5 °C/min, then to 230 °C at 3 °C/min, then to 265 °C at 10 °C/min, and then finally to 320 °C at 30 °C/min and holding for 4 min. Transfer interface temperature was 300 ˚C. The MS was operated in electron ionisation mode at 70 eV with source temperature 300 ˚C. Quadrupoles were held at 150 ˚C. Nitrogen was used as collision gas at a flow rate of 1.5 mL/min, while helium was used as quench gas at a flow rate of 4 mL/min. Solvent delay was 3.75 min. Data acquisition was in multiple reaction monitoring (MRM) mode. A minimum of two MRM transitions were determined for each analyte. Results are presented in Table S.11.

The triplicate data was treated under a high throughput differential metabolite level analysis using the linear models for microarray data (*Limma* & *Glimm*a) packages in R from Bio-conductor, resulting in the adjusted p-values of each enzymatic treatment relative to the control sample.[16,17] Subsequent R analysis was performed to calculate the standard error (SEM). The results of the targeted detection are summarised in Table S.11 and were visualised using the R heatmap (Figure 8).

Results

The concentration of 33 steroids in the urine samples was determined at the National Measurement Institute (NMI), Sydney. The same enzymatic deconjugation protocol as for the untargeted analysis was used to deconjugate sulfates. As a comparison, all samples were also treated by *E. coli* β-glucuronidase. The glucuronidase incubation mixtures consisted of 2.1 mL urine, 60 µL mixed internal standard, 1.5 mL phosphate buffer (0.2 M, pH 7) and 50 µL β-glucuronidase. These samples were incubated for 1.5 hours at 50 °C. All samples were then extracted by SPE, using 3M Empore C18-cartridges. The samples were loaded after conditioning with 2 mL methanol and 2 mL water. The cartridges were then washed with 1 mL water, 0.5 mL of 25% methanol in water, followed by 1 mL hexane. After drying the cartridges with air, they were eluted with 2 x 1 mL of 5% methanol in ethyl acetate. As an internal standard, 45 µL of 4 ppm methyltestosterone was added to the eluate. The samples were evaporated to dryness and stored frozen until derivatization. The derivatization was performed by adding 55 µL of MSTFA/TMSI/DTT (1000:2:4), followed by heating at 60 °C for 20 minutes, after which the samples were transferred to GC-vials and analysed by GC-MS/MS. The identification of steroids was performed by comparing the retention times and MRM ion ratios to those of the corresponding calibration standards in the MassHunter Quantitative Analysis instrumentation software (Agilent Technologies Australia). The steroid concentrations were calculated from external calibration curves. Low, medium, and high QC

urines were run with every batch and their quantitative results are continuously monitored in QC charts.

The urinary steroid profiling procedure was validated for the purposes of the doping control of endogenous anabolic androgenic steroids (such as testosterone) according to the requirements set out in the applicable World Anti-doping Agency technical document (WADA TDEAAS).[24] The six components of the steroidal module of the athlete biological passport (testosterone, epitestosterone, androsterone, etiocholanolone, 5α-androstan-3α,17β-diol and 5β-androstan-3α,17β-diol) have been quantitatively validated and meet the specifications as outlined in the technical document. The identification of steroids was performed by comparing the retention times and fragmentation ratios to those of corresponding deuterated internal standards (one for each analyte). Steroid reference materials for GC/MS experiments were obtained from Steraloids (Newport, RI, USA), Merck (Darmstadt, Germany), Toronto Research Chemicals (Ontario, Canada), Atlan Chim Pharma (Saint Herblain, France) or National Measurement Institute Australia (NMIA, North Ryde, Australia). The stable isotopically labelled steroidal components of the internal standard mixture consisted of d3-testosterone, d4-epitestosterone, d4-androsterone glucuronide, d5-etiocholanolone, d3-5α-androstan-3α,17β-diol and d5-5β-androstan-3α,17β-diol and were all obtained from NMIA. The remaining steroid analytes which do not form part of the steroidal module of the athlete biological passport have been validated in a semi-quantitative manner, as for these analytes deuterated internal standards are not available or have not been utilised.

Discussion

A targeted GC-MS study analysis of 33 steroids was designed to compare the efficacy of the PaS sulfatase mutants and the wild type with respect to the industry standard of β-glucuronidase. The results are summarised as a heat map to show concentrations of all steroids increased when treated with β-glucuronidase, indicating that all exist as glucuronide metabolites (Figure 8). They also showed that the concentration of 14 steroids increased significantly after incubation with one or more of the PaS-enzymes, indicating that those steroids have a portion of metabolites that exist in sulfated form. Eight of these steroids, clustered in blue, showed an increase of concentration greater in magnitude in the sulfatase enzymes than that of the β-glucuronidase, indicating that these may exist in a larger ratio of sulfated than glucuronide metabolites.

S.4 Experimental for the Synthesis of Reference Materials and Stable Isotope Labelled Internal Standards

Instrumentation for compound characterisation

Unless otherwise specified, ^{1}H NMR and ^{13}C NMR spectra were recorded in deuterated chloroform ($CDCl_3$), deuterated methanol (CD_3OD), or deuterium monoxide (D_2O), using a Bruker Avance 400 MHz or 700MHz spectrometer at 298 K. Chemical shifts are reported in parts per million (ppm) downfield shift from TMS (δ=0), as follows: chemical shift (δ) (multiplicity, coupling constant(s) *J* (Hz), relative integral), where multiplicity is defined as, s = singlet, d = doublet, t = triplet, q = quartet, m = multiplet, or combinations of the above. The signal due to the residual protonated solvent (i.e., $CHCl_3$) were used as an internal reference. Unless otherwise specified, low resolution mass spectrometry (LRMS) for compound characterisation was performed using negative or positive electrospray ionisation (ESI) on a Micromass ZMD ESI-Quad, and high-resolution mass spectrometry (HRMS) for compound characterisation was performed using negative or positive electrospray ionisation (ESI) on a Waters LCT Premier XE mass spectrometer or Thermo-Fischer Scientific Orbitrap Elite™ Hybrid Ion Trap-Orbitrap mass spectrometer. Infrared spectra were recorded on a Perkin-Elmer 1800 Series FTIR spectrometer. Melting points were measured on an SRS Opti-melt MPA 100 automated melting point system and are uncorrected. Reactions were monitored by analytical thin layer chromatography (TLC) performed on aluminium-backed 0.2 mm thick silica gel 60 F_{254} plates as supplied by Merck. Eluted plates were visualised by staining using a solution of sulfuric acid: methanol (5% v/v), followed by heating. Flash chromatographic separations were carried out following protocols defined by Still *et al.*[25] with silica gel 60 (40 – 63μm) as the stationary phase and analytical reagent (AR) or HPLC grade solvents as indicated. Tetrahydrofuran (THF), methanol, and dichloromethane were dried using a glass contour solvent purification system based on technology originally described by Grubbs *et al.*[26] Optical rotations were performed on a Rudolph Research Analytical, Autopol I Automatic Polarimeter (589 nm fixed wavelength, 10 mm cell).

Chemicals and reagents for synthesis

If not otherwise specified, chemicals were purchased from Sigma-Aldrich (Castle Hill, Australia). Nandrolone (17β-hydroxyestr-4-en-3-one) and cholanediol (5-androstene-3β,17β-diol, 5β-cholane-3α,24-diol), etiocholanolone, testosterone, epiandrosterone, androsterone, were purchased from Steraloids, Inc. (Newport, RI, USA). The *Pseudomonas aeruginosa* arylsulfatase (PaS) enzymes, wild-type (WT-PaS), LEF mutant (LEF-PaS), and PVFV mutant (PVFV-PaS),were expressed and purified as previously described,[8,27] *Helix pomatia* arylsulfatase (HpS) preparation (S92626-10KU, ≥ 10000 Units of sulfatase, ≥ 300000 Units of β-glucuronidase) was purchased from Sigma-Aldrich (MERCK). Dimethylformamide, 99.8% was purchased from Chem-Supply (Gillman, Australia) and methanol-d4, 99.8% D from Cambridge Isotope Laboratories, Andover, MA, USA). MilliQ water was used in all aqueous solutions during sample treatment. LiChrosolv LC-MS grade water and methanol and ammonium formate (99.995 %) from Merck (Bayswater, Australia) was used in the liquid chromatography mobile phases. Solid phase extraction was performed using Sep-Pak C18 Vac 3 cc SPE-cartridges and Oasis WAX 3 cc SPE-cartridges cartridges from Waters (Rydalmere, Australia). All UHPLC samples were filtered using 0.2 μm spin filters from PhaseSep (Doncaster East, Australia). Weighing of internal standards and reference compounds was performed on an Ultra-micro balance UMX2 from Mettler Toledo (Port Melbourne, Australia), with a readability of ±0.0001 mg The internal standards nandrolone sulfate, cholanediol bis(sulfate), stable isotope labelled (SIL) epiandrosterone [18O3]-sulfate

and 5α-androstane-3β,17β-diol 3,[$^{18}O_3$]17-bis(sulfate), were prepared as the corresponding ammonium salts (supplementary information).

Synthesis of internal standards and reference materials

S.4.1 General procedure for small scale steroid conjugate purification by C18 SPE

This was used to separate steroid conjugates from reagents in a reaction mixture or excess ammonium salt (as determined by ^{1}H NMR). The procedure was adapted from literature.[28,29] A C18 SPE cartridge (3 cc) was pre-conditioned with methanol (3 mL) followed by water (3 mL), under positive pressure of nitrogen. The reaction mixture was then loaded onto the cartridge. The cartridge was washed with aqueous ammonium in water (2% v/v, 2 mL), and then water (3 mL). The sulfate conjugate was then eluted with methanol (9 mL) and then concentrated under reduced pressure to yield the steroid conjugate at the ammonium salt. Percentage conversion was then determined by ^{1}H NMR, using the integration of a suitable signal present in both the starting material and product.

S.4.2 General procedure for the separation of conjugated and free steroid

This was used to separated conjugated steroids from any free steroid in a reaction mixture. The procedure was adapted from literature.[28,29] A WAX SPE cartridge (6 cc) was pre-conditioned with methanol (5 mL) followed by water (15 mL), under positive pressure of nitrogen. The conjugate/free steroid mixture was then loaded onto the cartridge. The cartridge was then washed with formic acid in water (2% v/v, 15 mL), water (15 mL) and then methanol (15 mL) to elute any free steroid. Finally, the steroid conjugate was eluted using aqueous ammonia solution in methanol (5% v/v, 15 mL). The eluted fraction was then concentrated under reduced pressure to yield the steroid conjugate as the ammonium salt.

S.4.3 General procedure for small scale steroid sulfation reaction

This was used to undergo conjugation of either sulfate or SIL-sulfate to a free steroid. The procedure was adapted from the literature.[30] Using either ^{18}O-Labelled sulfuric acid (10.0 µL, 173 µmol) or unlabelled sulfuric acid (10.0 µL, 176 µmol), were combined with freshly distilled acetic anhydride (20.0 µL, 212 µmol) and stirred to this anhydrous pyridine (200 µL, 2.48 mmol) was then added. The resulting solution was then stirred at room temperature for 5 min. The steroid (5.0-10 mg) in pyridine (200 µL, 2.48 mmol) was then added. The reaction was capped and stirred at room temperature for 3 hours. The reaction was then quenched with water (10 mL) and subjected to purification by C18 SPE or WAX SPE as outlined in general procedures S.4.1 and S.4.2.

S.4.4 General procedure for small scale reduction of steroid conjugates

This reduction was primarily used to reduce saturated ketones of mono-sulfates. The procedure was adapted from literature.[28] Sodium borohydride (7.0 mg, 0.19 mmol) was added slowly (over one minute) to an ice cooled stirring solution of steroid conjugate (5.0 -10 mg) in methanol (100 µL). After the reaction subsided (no gas evolution observed) it was capped and stirred at room temperature for 2 hours. The reaction was then quenched by the addition of water (7-8 mL) and adjusted to pH 7 (universal indicator strips) by addition of aqueous hydrochloric acid (0.1 M, ≈ 2-3 mL). The resulting solution was then subjected to purification by general procedure S.4.1.

S.4.5 Nandrolone-sulfate, ammonium salt (**S1**)[8]

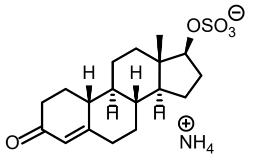

Nandrolone (10 mg, 36.4 μmol) was reacted according to general procedure S.4.3, using unlabelled sulfuric acid, giving the title compound as a colourless solid (> 98 % conversion). ^{1}H NMR (700 MHz, CD_3OD) δ 5.81 – 5.77 (m, 1H), 4.25 (t, *J* = 9.1, 7.9 Hz, 1H), 2.52–2.48 (m, 1H), 2.40–2.27 (m, 4H), 2.23–2.14 (m, 2H), 2.01–1.97 (m, 1H), 1.90 – 1.85 (m, 2H), 1.80 – 1.72 (m, 1H), 1.69 – 1.62 (m, 1H), 1.58 – 1.50 (m, 1H), 1.49–1.42 (m, 1H), 1.42–1.28 (m, 3H), 1.27–1.20 (m, 1H), 1.13–1.03 (m, 2H), 0.88 (s, 3H):^{13}C NMR (176 MHz, CD_3OD) δ 202.9, 170.8, 124.7, 88.0, 51.0, 50.5, 44.0, 43.8, 41.5, 37.7, 37.3, 36.5, 31.9, 29.1, 27.7, 27.1, 24.1, 12.1: LRMS (-ESI): *m/z* 353.2 (100%, $[C_{18}H_{25}O_5S]^-$): HRMS (-ESI) *m/z* 353.14282 calcd. for $[C_{18}H_{25}O_5S]^-$ ($[M-NH_4]^-$), found 353.14144. NMR spectra matched that in the literature.[8]

S.4.6 Cholane-diol bis(sulfate), ammonium salt(**S2**)

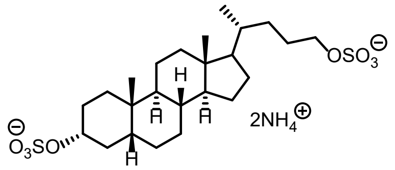

Cholane diol (5.00 mg, 13.8 μmol) was reacted according to the general procedure S.4.3, using unlabelled sulfuric acid. This yielded the title compound as a colourless solid (> 98 % conversion). ^{1}H NMR (700 MHz, CD_3OD) δ 4.27 (m, 1H), 3.96 (t, *J* = 6.4 Hz, 2H), 3.00 (s, 1H), 2.86 (s, 1H), 2.01 (m, 1H), 1.95–1.80 (m, 5H), 1.78–1.73 (m, 2H), 1.66–1.38 (m, 8H), 1.36–0.99 (m, 10H), 0.99–0.92 (m, 6H), 0.69 (s, 3H). ^{13}C NMR (176 MHz, CD_3OD) δ 80.4, 69.6, 57.8, 57.6, 43.9, 43.7, 41.8, 41.5, 37.2, 36.8, 36.4, 35.6, 34.6, 33.1, 29.3, 28.9, 28.3, 27.6, 27.2, 25.3, 23.8, 21.9, 19.1, 12.5. LRMS (-ESI): *m/z* 260.3 (100 %, $[C_{24}H_{41}O_8S_2]^{2-}$); HRMS (-ESI) *m/z* calcd. for $[C_{24}H_{41}O_8S_2]^{2-}$ ($[M-2NH_4]^{2-}$) 260.1088, found 260.1088.

S.4.7 Epiandrosterone [$^{18}O_3$]-sulfate, ammonium salt (**S3**)[28]

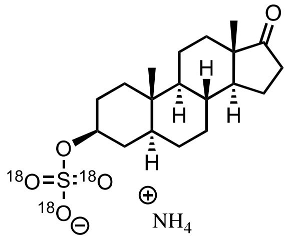

Epiandrosterone (5.00 mg, 17.2 μmol) was reacted according to general procedure S.4.3, using labelled sulfuric acid. This gave the title compound as a colourless solid (> 98% conversion). ^{1}H NMR (700 MHz, CD_3OD) ^{1}H NMR (400 MHz, CD_3OD): δ 4.25 (m, 1H, C3-H), 2.43 (dd, *J* 19.1, 8.8, 1H, C16-H), 2.13–1.90 (m, 3H), 1.86–1.16 (m, 15H), 1.10–0.96 (m, 2H), 0.88 (s, 3H, C18-H_3), 0.87 (s, 3H, C19-H_3), 0.76 (m, 1H); ^{13}C NMR (101 MHz, CD_3OD) δ 224.06 (C17), 79.53 (C3), 55.78, 52.70, 46.28, 38.16, 36.71, 36.65, 36.36, 36.30, 32.80, 32.04, 29.73, 29.58, 22.72, 21.58, 14.20 (C18), 12.60 (C19), one carbon overlapping or obscured; LRMS (-ESI): *m/z* 375.3 (100%, $[C_{19}H_{29}[^{18}O_3]O_2S]^-$); HRMS (-ESI) *m/z* calcd. For $[C_{19}H_{29}[^{18}O_3]O_2S]^-$ ($[M-NH_4]^-$) 375.1863, found 375.1864. NMR spectra matched the literature for the unlabelled compound.[28]

S.4.8 Testosterone sulfate, ammonium salt (**1**)[28]

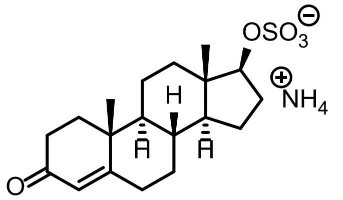

Testosterone (5.00 mg, 17.3 µmol) was reacted according to general procedure S.4.3, using unlabelled sulfuric acid. This yielded the title compound as a colourless solid (> 98% conversion). ^{1}H NMR (400 MHz, CD_3OD) δ 5.71 (s, 1H, C4-H), 4.23 (m, 1H, C3-H), 2.55–2.39 (m, 2H), 2.35–2.23 (m, 2H), 2.24–1.97 (m, 3H), 1.90 (m, 1H), 1.80–1.57 (m, 5H), 1.55 – 1.32 (m, 2H), 1.24 (s, 3H, C18-H_3), 1.21 (m, 1H), 1.12 – 0.94 (m, 3H), 0.87 (s, 3H, C19-H_3); LRMS (-ESI) *m/z* 367.2 (100%, $[C_{19}H_{27}O_5S]^-$); HRMS (-ESI) *m/z* calcd. for $[C_{19}H_{27}O_5S]^-$ ($[M-NH_4]^-$) 367.1574, found 367.1581. Spectroscopic data was found to match the literature.[28]

S.4.9 Epiandrosterone 3-sulfate, ammonium salt (**2**)[28]

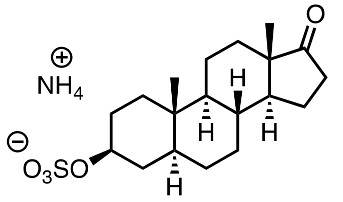

Epiandrosterone (5.00 mg, 17.2 µmol) was reacted according to general procedure S.4.3, using unlabelled sulfuric acid. This yielded the title compound as a colourless solid (> 98 % conversion). ^{1}H NMR (400 MHz, CD_3OD): δ 4.25 (m, 1H, C3-H), 2.42 (m, 1H, C16-H), 2.11–1.91 (m, 3H), 1.85–1.16 (m, 15H), 1.14–1.00 (m, 2H), 0.88 (s, 3H, C18-H_3), 0.87 (s, 3H, C19-H_3), 0.75 (m, 1H); LRMS (-ESI) *m/z* 369.3 (100%, $[C_{19}H_{29}O_5S]^-$); HRMS (-ESI) *m/z* calcd. for $[C_{19}H_{29}O_5S]^-$ ($[M-NH_4]^-$) 369.1738, found 369.1730. Spectroscopic data and spectra were found to match that in the literature for the compound.[28]

S.4.10 5α-Androstane-3β,17β-diol 3-sulfate, ammonium salt (**S5**)

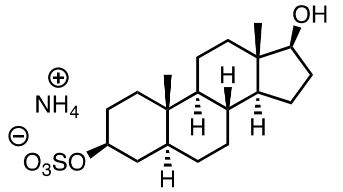

Epiandrosterone 3-sulfate, ammonium salt (**2**) (5.00 mg, 13.6 µmol) was reacted according to general procedure S.4.4. This gave the title compound as a colourless solid (> 98% conversion). ^{1}H NMR (400 MHz, CD_3OD): δ 4.25 (m, 1H, C3-H), 3.56 (t, J 8.6, 1H, C17-H), 2.05–1.90 (m, 2H), 1.87–1.65 (m, 4H), 1.64–1.11 (m, 11H), 1.10–0.85 (m, 4H), 0.86 (s, 3H, C18-H_3), 0.72 (s, 3H C19-H_3), 0.67 (m, 1H); ^{13}C NMR (101 MHz, CD_3OD) δ 82.5 (C3), 79.7, 55.9, 52.4, 46.4, 44.1, 38.3, 38.1, 36.9, 36.6, 36.4, 32.8, 30.7, 29.8, 24.3, 21.9, 12.7 (C18), 11.7 (C19), one peak overlapping or obscured; LRMS (-ESI) *m/z* 371.3 (100%, $[C_{19}H_{31}O_5S]^-$); HRMS (-ESI) *m/z* calcd. for $[C_{19}H_{31}O_5S]^-$ ($[M-NH_4]^-$) 371.1899, found 371.1892.

S.4.10 5α-Androstane-3β,17β-diol 3,[$^{18}O_3$]17-diol bis(sulfate) (**S4**)[4]

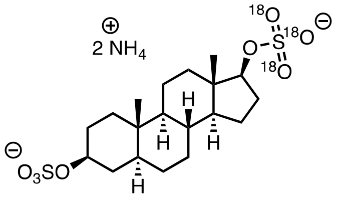

5α-Androstane-3β,17β-diol 3-sulfate, ammonium salt (**S5**) (5.00 mg, 13.5 μmol), was reacted according to general procedure S.4.3, using labelled sulfuric acid. This yielded the title compound as a colourless solid (> 98% conversion). ^{1}H NMR (700 MHz, CD_3OD): δ 4.27–4.18 (m, 2H, C3-H & C17-H), 2.15 (m, 1H), 2.03 (m, 1H), 1.94 (m, 1H), 1.84–1.67 (m, 4H), 1.64–1.49 (m, 3H), 1.49–1.38 (m, 2H), 1.35–1.24 (m, 4H), 1.21–1.12 (m, 2H), 1.09–0.98 (m, 2H), 0.94 (m, 1H), 0.86 (s, 3H, C18-H3), 0.80 (s, 3H, C19-H3), 0.70 (m, 1H); ^{13}C NMR (151 MHz, CD_3OD) δ 88.2, 79.7, 55.8, 51.8, 46.3, 44.0, 38.2, 38.0, 36.8, 36.6, 36.4, 32.8, 29.8, 29.2, 24.4, 21.8, 12.7 (C18), 12.2 (C19), one carbon overlapping or obscured; LRMS (-ESI) *m/z* 228.2 (100%, $[C_{19}H_{30}[^{18}O_3]O_5S_2]^{2-}$); HRMS (-ESI) *m/z* calcd. for $[C_{19}H_{30}[^{18}O_3]O_5S_2]^{2-}$ ($[M\text{-}2NH_4]^{2-}$) 228.07602, found 228.07650. The NMR spectra matched those in the literature for the unlabelled compound.[4]

S.4.12 17α-Acetoxy-5α-androstane-3-one (**S6**)

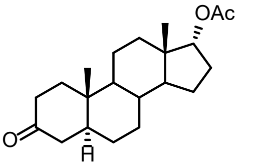

Acetic anhydride (0.5 mL, 5.29 mmol) was added dropwise to a stirring solution of epidihydrotestosterone (20.00 mg, 68.8 μmol) and DMAP (2 mg, 17.2 μmol) in pyridine (0.5 mL), and left at room temperature under a nitrogen atmosphere for 5 hours. The reaction was then extracted with water (1.5 mL), followed by ether (7.5 mL), and the organic phase washed with HCl (3 mL, 1M) followed by saturated sodium bicarbonate solution (3 mL). The organic layer was then dried with anhydrous magnesium sulfate and concentrated under reduced pressure. The crude was subjected to flash chromatography, which yielded the title compound as a colourless solid (20.2 mg, 60.8 μmol, 88 % yield).^{1}H NMR (600 MHz, CD_3OD); δ 4.81 (d, *J* = 6.4 Hz, 1H), 2.47–2.25 (m, 3H), 2.25–2.14 (m, 1H), 2.13–2.09 (m, 1H), 2.08–2.05 (m, 0H), 2.03 (s, 3H), 2.03–1.98 (m, 1H), 1.81–1.71 (m, 2H), 1.68–1.10 (m, 13H), 1.01 (s, 3H), 1.02–0.94 (m, 1H), 0.76 (s, 3H); ^{13}C NMR (101 MHz, $CDCl_3$) δ 212.14, 170.85, 82.00, 53.65, 50.01, 46.84, 44.90, 44.83, 38.75, 38.32, 35.89, 35.74, 32.13, 31.93, 30.14, 29.03, 24.81, 21.45, 21.00, 16.81, 11.64; LRMS (+ESI): *m/z* 687.4 (100 %, $[(C_{21}H_{32}O_3)_2Na]^+$); HRMS (+ESI) *m/z* calcd. for $[C_{21}H_{32}O_3Na]^+$ ($[M+Na]^+$), 355.2249, found 355.2236. Spectroscopic data and spectra was found to match the literature for the compound.[31]

S.4.13 17α-Acetoxy-5α-androstane-3β-sulfate, ammonium salt (**S7**)

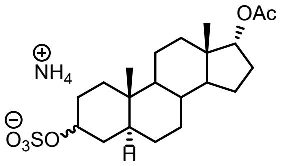

17α-Acetoxy-5α-androstane-3-one (**S6**) (20 mg, 60.2 µmol) was reacted according to general procedure S.4.4. Following this the crude 17α-acetoxy-5α-Androstane-3β-ol (12.8mg, 38.3 µmol, 64 % isolated yield) was reacted according to general procedure S.2.3. This gave the title compound as a colourless solid (14.4 mg, 34.8 µmol, 58 % isolated yield with a ratio of 1:8 of the 3α-OH : 3β-OH epimers). Data reported for major epimer. ^{1}H NMR (600 MHz, MeOD); δ 4.76 (d, J = 6.3 Hz, 1H), 4.30–4.22 (m, 1H), 2.24–2.13 (m, 1H), 1.85–1.72 (m, 4H), 1.67–1.28 (m, 13H), 1.27–1.14 (m, 2H), 1.09–0.96 (m, 3H), 0.86 (s, 3H), 0.84–0.79 (m, 1H), 0.77 (s, 3H), 0.74–0.68 (m, 1H); ^{13}C NMR (151 MHz, MeOD) δ 172.66, 83.50, 79.63, 55.55, 51.34, 46.26, 46.01, 38.29, 37.04, 36.55, 36.33, 33.57, 33.10, 30.82, 29.79, 29.77, 25.60, 21.74, 21.11, 17.09, 12.65; LRMS (-ESI): *m/z* 413.1 (100%, $[C_{21}H_{33}O_6S]^-$); HRMS (-ESI) *m/z* calcd. for $[C_{21}H_{33}O_6S]^-$ ($[M-NH_4]^-$), 413.1998 found 413.2001.

S.4.14 5α-Androstane-3β-17α-diol 17-sulfate, ammonium salt (**3**)

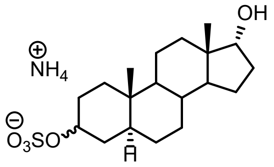

17α-Acetoxy-5α-androstane-3β-sulfate, ammonium salt (**S7**) (14.4 mg, 34.8 µmol) and potassium carbonate (12 mg) were suspended in methanol (2mL), the solution was then stirred for 3 days. After which the methanol was removed under reduced pressure, the crude was then dissolved in 10 mL of water and then purified according to general procedures S.2.1 and S.2.2 giving the title compound as a colourless solid (4 mg, 10.8 µmol, 31 % isolated yield, reported as ratio of1:14 of the 3α-OH : 3β-OH epimers). Data reported for the major epimer. ^{1}H NMR (400 MHz, MeOD) δ 4.33 (d, J = 5.7 Hz, 0H), 4.31–4.20 (m, 1H), 3.64 (d, J = 6.1 Hz, 1H), 2.22–2.08 (m, 2H), 2.09–1.97 (m, 2H), 1.89–1.70 (m, 4H), 1.71–1.53 (m, 2H), 1.52–1.28 (m, 10H), 1.27–1.13 (m, 2H), 1.12–0.97 (m, 2H), 0.88 (s, 3H), 0.79–0.69 (m, 1H), 0.68 (s, 3H); ^{13}C NMR (101 MHz, MeOD) δ 80.74, 79.69, 55.69, 49.94, 46.48, 46.30, 38.32, 37.17, 36.57, 36.35, 33.70, 32.83, 32.76, 29.87, 29.78, 25.61, 21.87, 17.59, 12.68; LRMS (-ESI): *m/z* 371.2 (100%, $[C_{19}H_{31}O_5S]^-$); HRMS (-ESI) *m/z* calcd. for $[C_{19}H_{31}O_5S]^-$ ($[M-NH_4]^-$), 371.1892 found 371.1903.

S.5 References

(1) Ligges, U.; Mächler, M. *J. Stat. Softw.* **2003**, *8* (11), 1–20.

(2) Broadhurst, D.; Goodacre, R.; Reinke, S. N.; Kuligowski, J.; Wilson, I. D.; Lewis, M. R.; Dunn, W. B. *Metabolomics* **2018**, *14* (6), 72.

(3) Hartigan, J. A.; Wong, M. A. *Appl. Stat.* **1979**, *28* (1), 100.

(4) McLeod, M. D.; Waller, C. C.; Esquivel, A.; Balcells, G.; Ventura, R.; Segura, J.; Pozo, Ó. J. *Anal. Chem.* **2017**, *89* (3), 1602–1609.

(5) Team, R. C. R Foundation for Statistical Computing: Vienna, Austria 2017.

(6) S Á Nchez-Guijo, A.; Oji, V.; Hartmann, M. F.; Traupe, H.; Wudy, S. A. *J. Lipid Res.* **2015**, *56*, 1843–1851.

(7) Association of Offical Racing Chemist. AORC MS Criteria (modified 23 Aug 16) - Association of Official Racing Chemists http://www.aorc-online.org/documents/aorc-ms-criteria-modified-23-aug-16/ (accessed Nov 5, 2020).

(8) Stevenson, B. J.; Waller, C. C.; Ma, P.; Li, K.; Cawley, A. T.; Ollis, D. L.; McLeod, M. D. *Drug Test. Anal.* **2015**, *7* (10), 903–911.

(9) Uduwela, D. R.; Pabis, A.; Stevenson, B. J.; Kamerlin, S. C. L.; Mcleod, M. D. **2018**.

(10) Chemical Entities of Biological Interest (ChEBI) https://www.ebi.ac.uk/chebi/init.do (accessed Okt 30, 2020).

(11) Palermo, A.; Botrè, F.; de la Torre, X.; Zamboni, N. *Anal. Chim. Acta* **2017**, *964*, 112–122.

(12) Fabregat, A.; Pozo, O. J.; Marcos, J.; Segura, J.; Ventura, R. *Anal. Chem.* **2013**, *85* (10), 5005–5014.

(13) Tsugawa, H.; Kanazawa, M.; Ogiwara, A.; Arita, M. *Bioinformatics* **2014**, *30* (16), 2379–2380.

(14) Tsugawa, H.; Cajka, T.; Kind, T.; Ma, Y.; Higgins, B.; Ikeda, K.; Kanazawa, M.; VanderGheynst, J.; Fiehn, O.; Arita, M. *Nat. Methods* **2015**, *12* (6), 523–526.

(15) Adusumilli, R.; Mallick, P. *Methods Mol. Biol.* **2017**, *1550*, 339–368.

(16) Su, S.; Law, C. W.; Ah-Cann, C.; Asselin-Labat, M.-L.; Blewitt, M. E.; Ritchie, M. E. *Bioinformatics* **2017**, *33* (13), 2050–2052.

(17) Ritchie, M. E.; Phipson, B.; Wu, D.; Hu, Y.; Law, C. W.; Shi, W.; Smyth, G. K. *Nucleic Acids Res.* **2015**, *43* (7), e47–e47.

(18) Considine, E. C.; Thomas, G.; Boulesteix, A. L.; Khashan, A. S.; Kenny, L. C. *Metabolomics* **2018**, *14* (1).

(19) Godzien, J.; Alonso-Herranz, V.; Coral, •; Emily, B. •; Armitage, G. *Metabolomics* **2015**, *11*, 518–528.

(20) Sangster, T.; Major, H.; Plumb, R.; Wilson, A. J.; Wilson, I. D. *Analyst* **2006**, *131* (10), 1075.

(21) Food and Drug Administration. *Guidance for Industry Bioanalytical Method Validation Guidance for Industry Bioanalytical Method Validation*; 2018.

(22) Mansilha, C.; Melo, A.; Rebelo, H.; Ferreira, I. M. P. L. V. O.; Pinho, O.; Domingues, V.; Pinho, C.; Gameiro, P. *J. Chromatogr. A* **2010**, *1217* (43), 6681–6691.

(23) Waller, C. C.; Cawley, A. T.; Suann, C. J.; Ma, P.; McLeod, M. D. *J. Pharm. Biomed. Anal.* **2016**, *124*, 198–206.

(24) WADA. *WADA Tech. Doc.* **2019**.

(25) Clark Still, W. *J . Org. Chem* **1978**, *43* (14).

(26) Pangborn, A. B.; Giardello, M. A.; Grubbs, R. H.; Rosen, R. K.; Timmers, F. J. *Organometallics* **1996**, *15* (5), 1518–1520.

(27) Uduwela, D. R.; Pabis, A.; Stevenson, B. J.; Kamerlin, S. C. L.; McLeod, M. D. *ACS Catal.* **2018**, *8* (9), 8902–8914.

(28) Waller, C. C.; McLeod, M. D. *Steroids* **2014**, *92*, 74–80.

(29) Ma, P.; Kanizaj, N.; Chan, S.-A.; Ollis, D. L.; McLeod, M. D. *Org. Biomol. Chem.* **2014**, *12* (32), 6208.

(30) Bureeva, S.; Andia-Pravdivy, J.; Petrov, G.; Igumnov, M.; Romanov, S.; Kolesnikova, E.; Kaplun, A.; Kozlov, L. *Bioorganic Med. Chem.* **2005**, *13* (4), 1045–1052.

(31) Pranata, A.; Fitzgerald, C. C.; Khymenets, O.; Westley, E.; Anderson, N. J.; Ma, P.; Pozo, O. J.; McLeod, M. D. *Steroids* **2019**, *143*.

S.6. Spectra of synthesised standards

Nandrolone Sulfate, ammonium salt (S1) LRMS

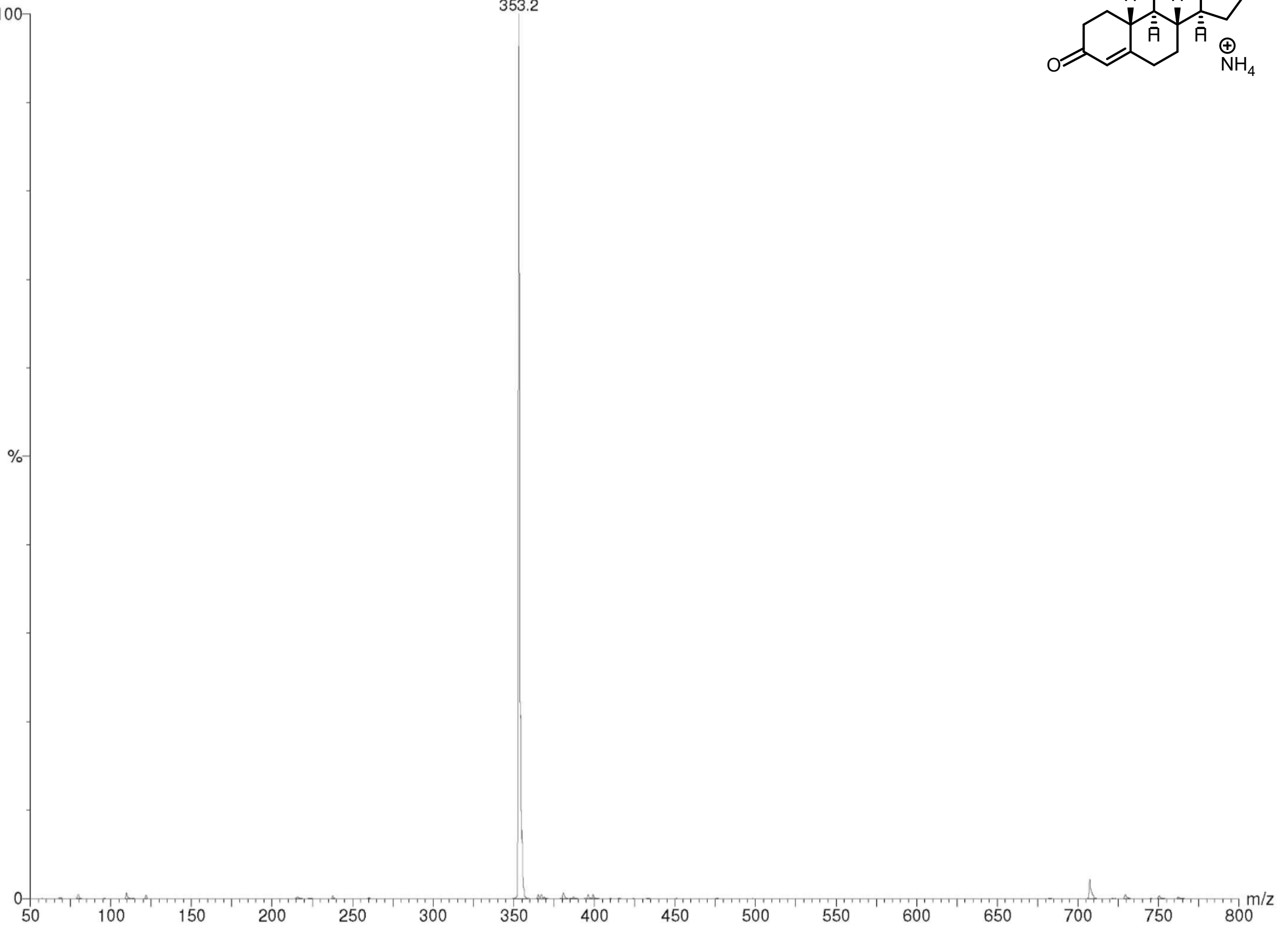

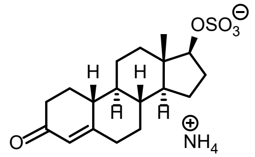

Nandrolone Sulfate, ammonium salt (S1) ^{1}H NMR 700 MHz, CD_3OD

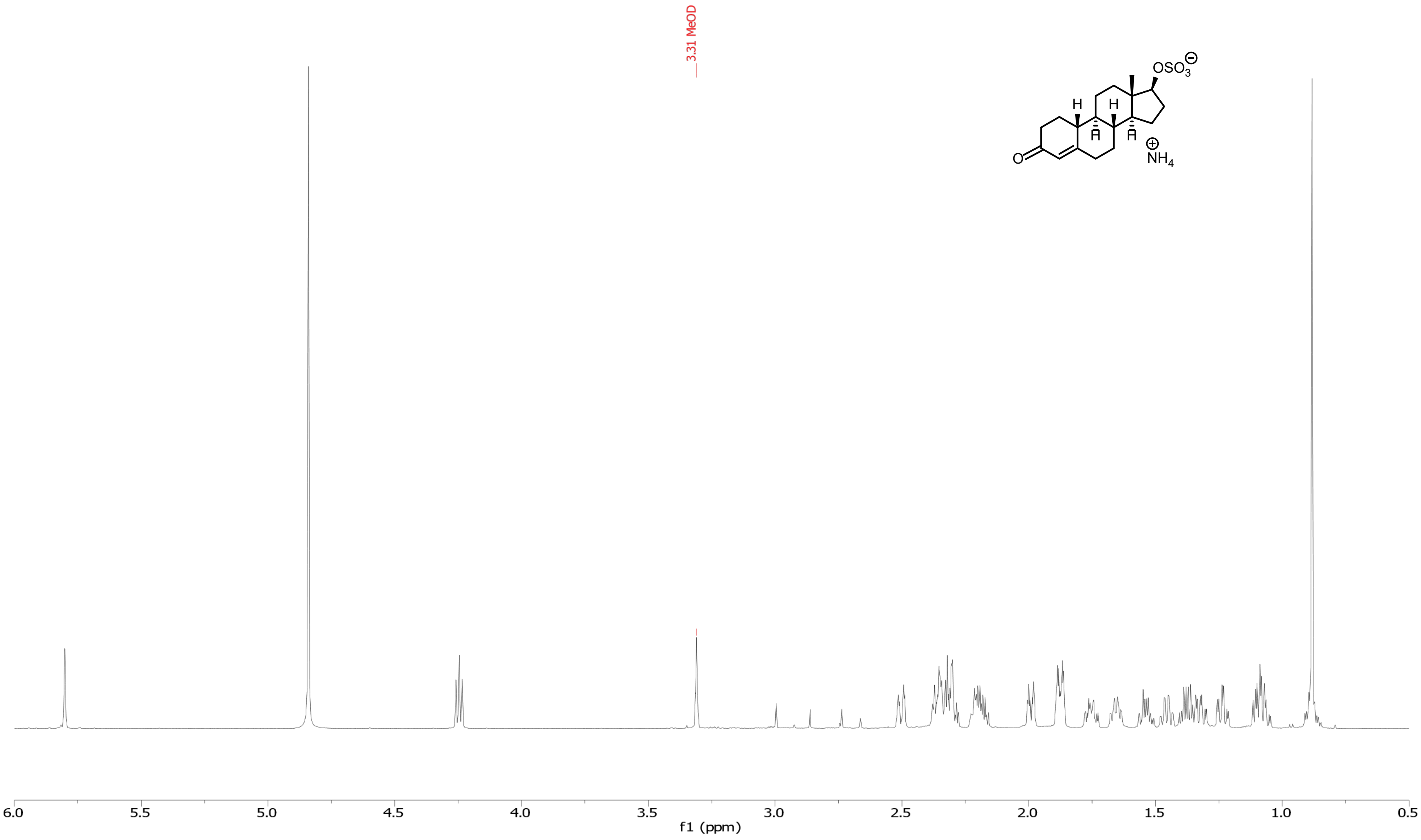

Nandrolone Sulfate, ammonium salt (S1) ^{13}C NMR 176 MHz, CD_3OD

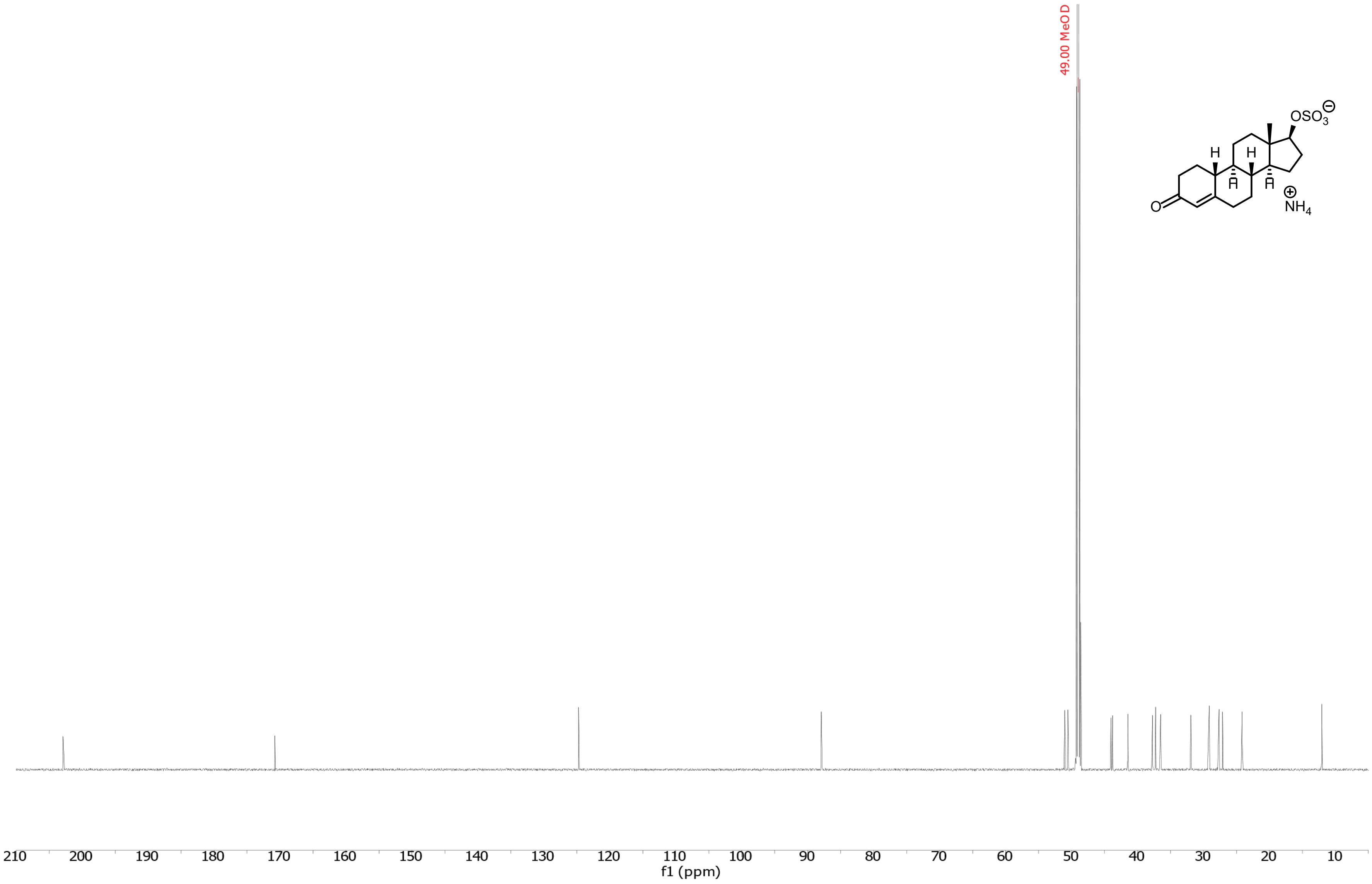

Cholane Diol Bis(sulfate), ammonium salt (S2), LRMS

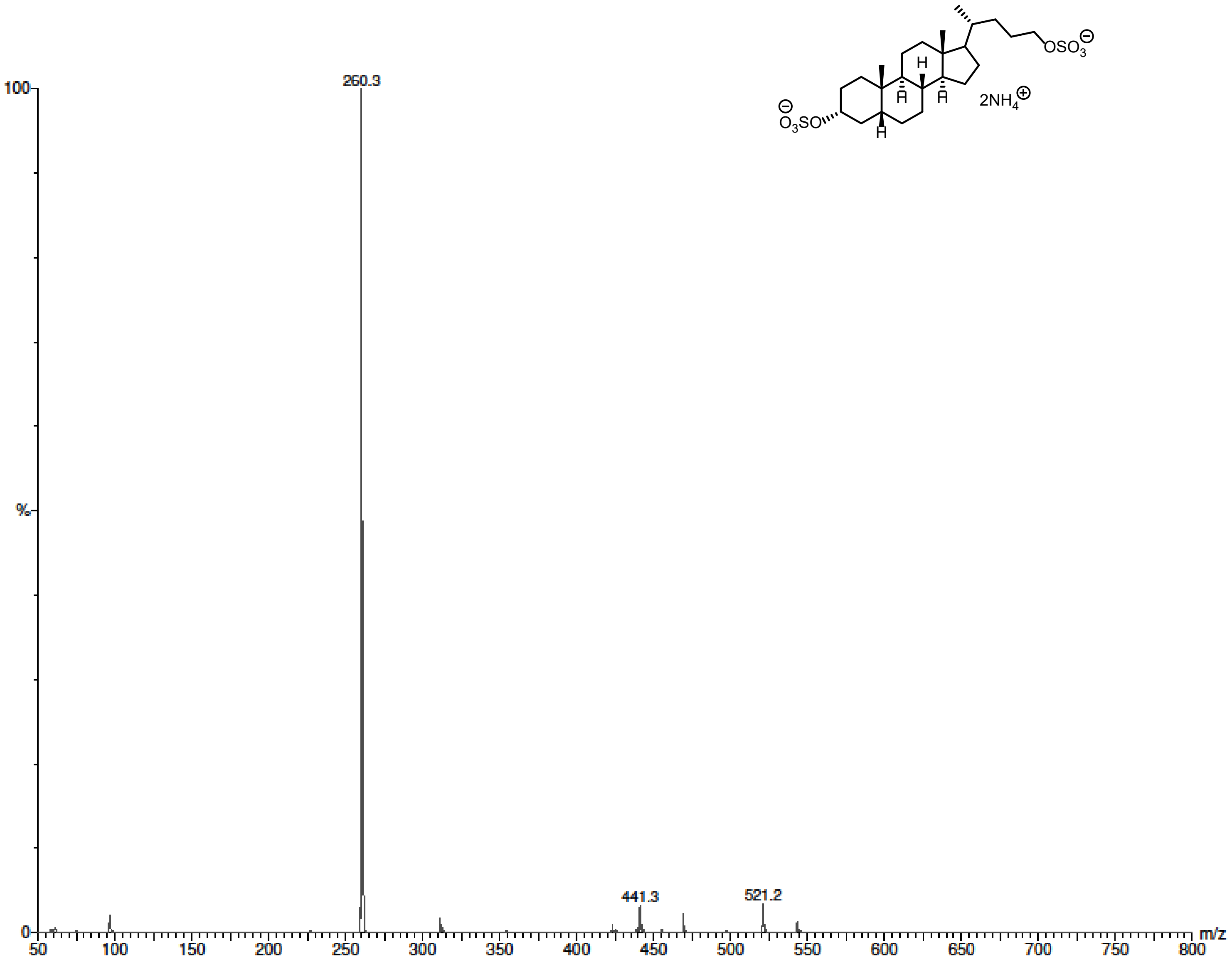

Cholane diol Bis(sulfate), ammonium salt (S2) ^{1}H NMR 400 MHz, CD3OD

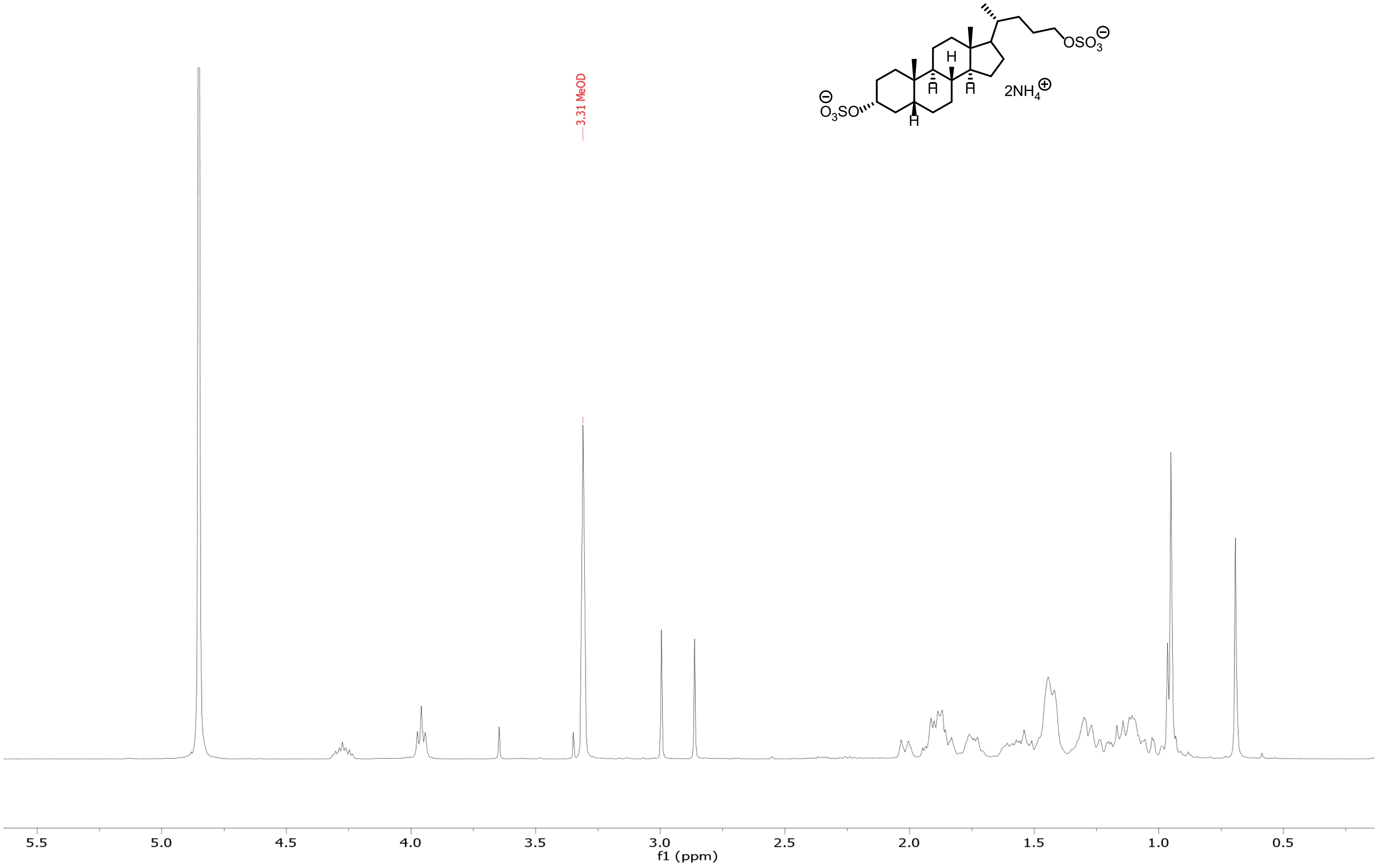

Cholane diol Bis(sulfate), ammonium salt (S2) ^{13}C NMR 101 MHz, CD_3OD

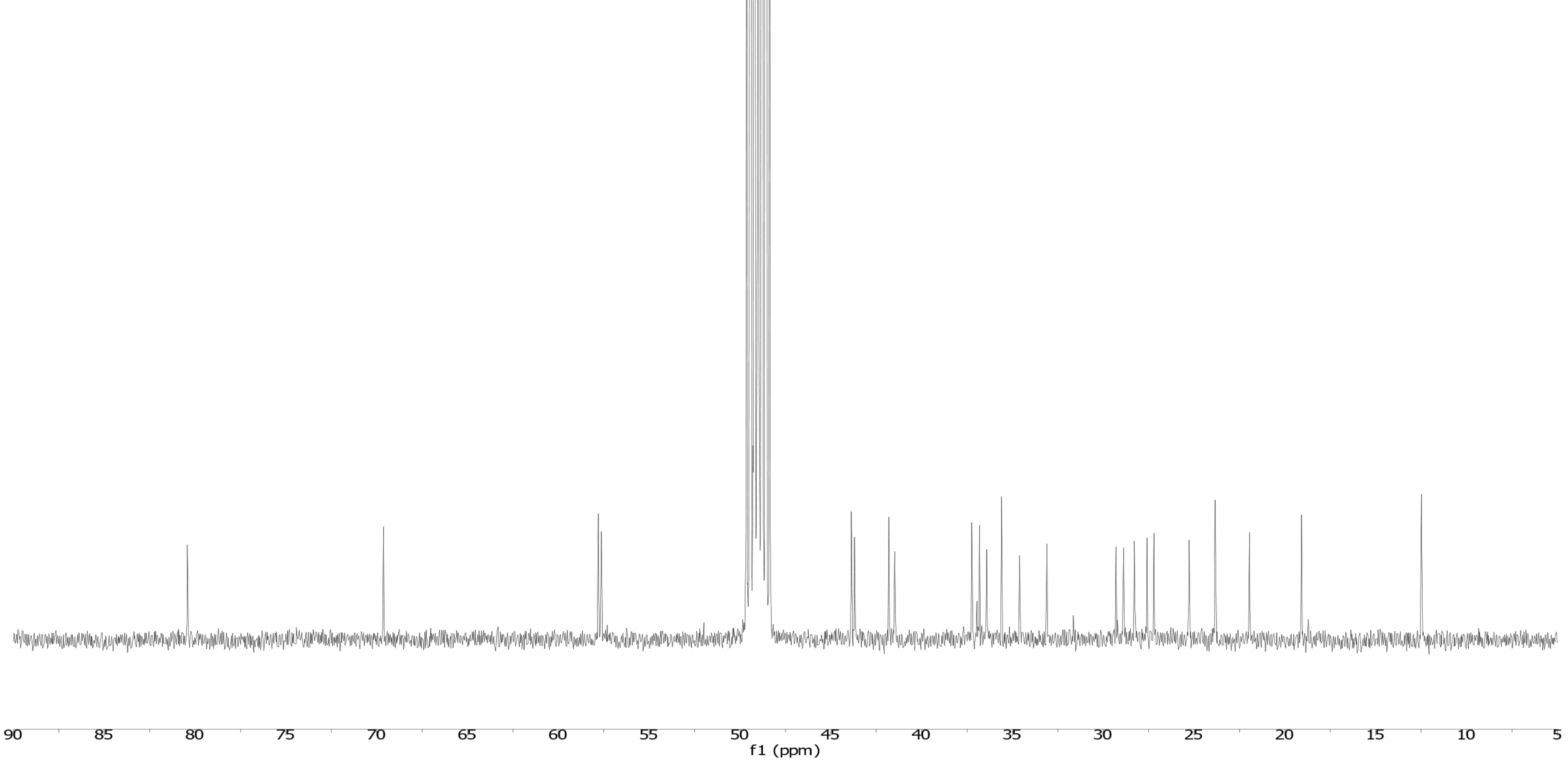

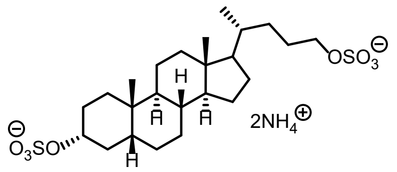

Epiandrosterone 3[$^{18}O_3$]-sulfate, ammonium salt (S3) LRMS

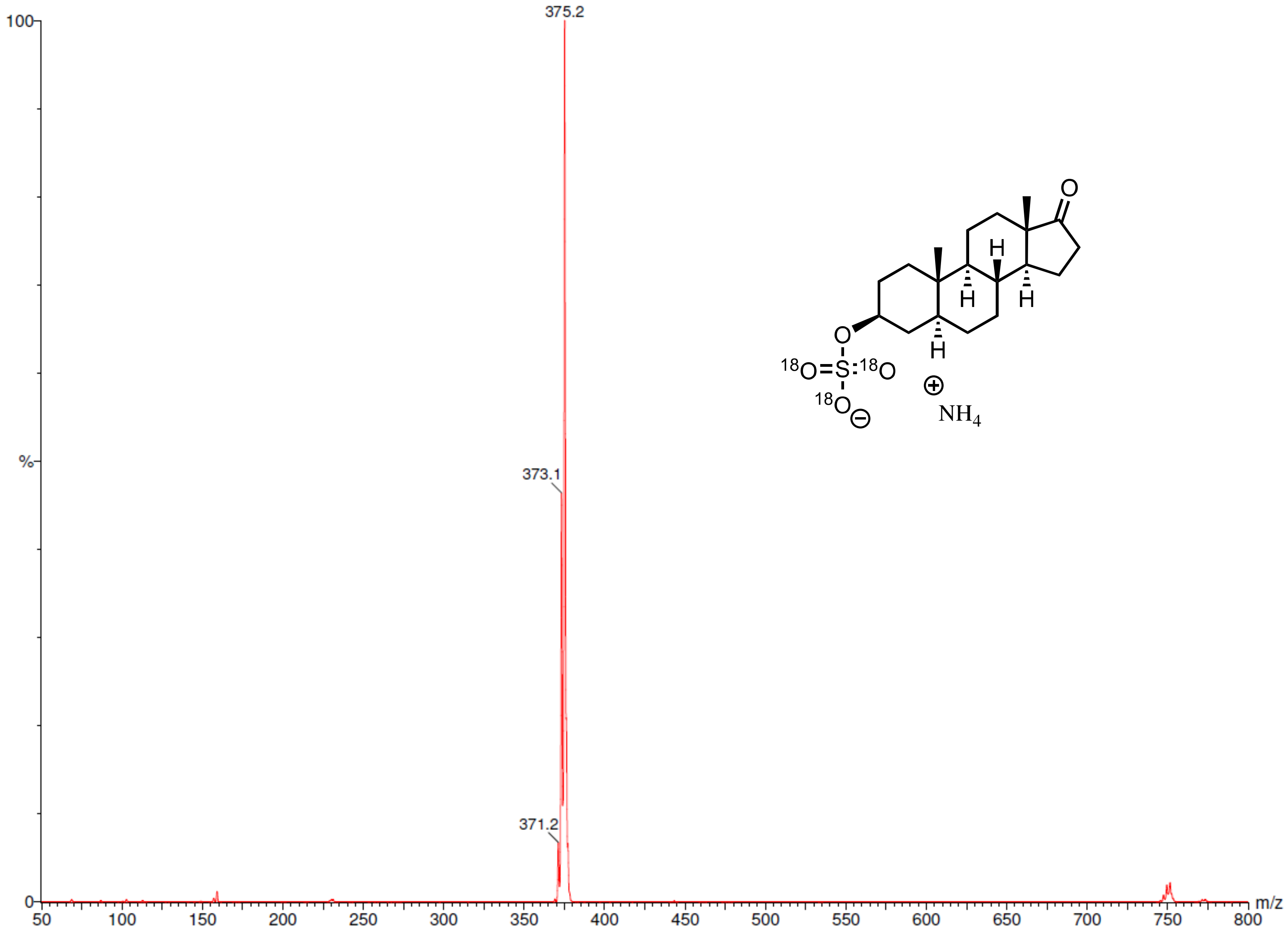

Epiandrosterone 3[$^{18}O_3$]-sulfate, ammonium salt (S3) ^{1}H NMR 400 MHz, CD_3OD

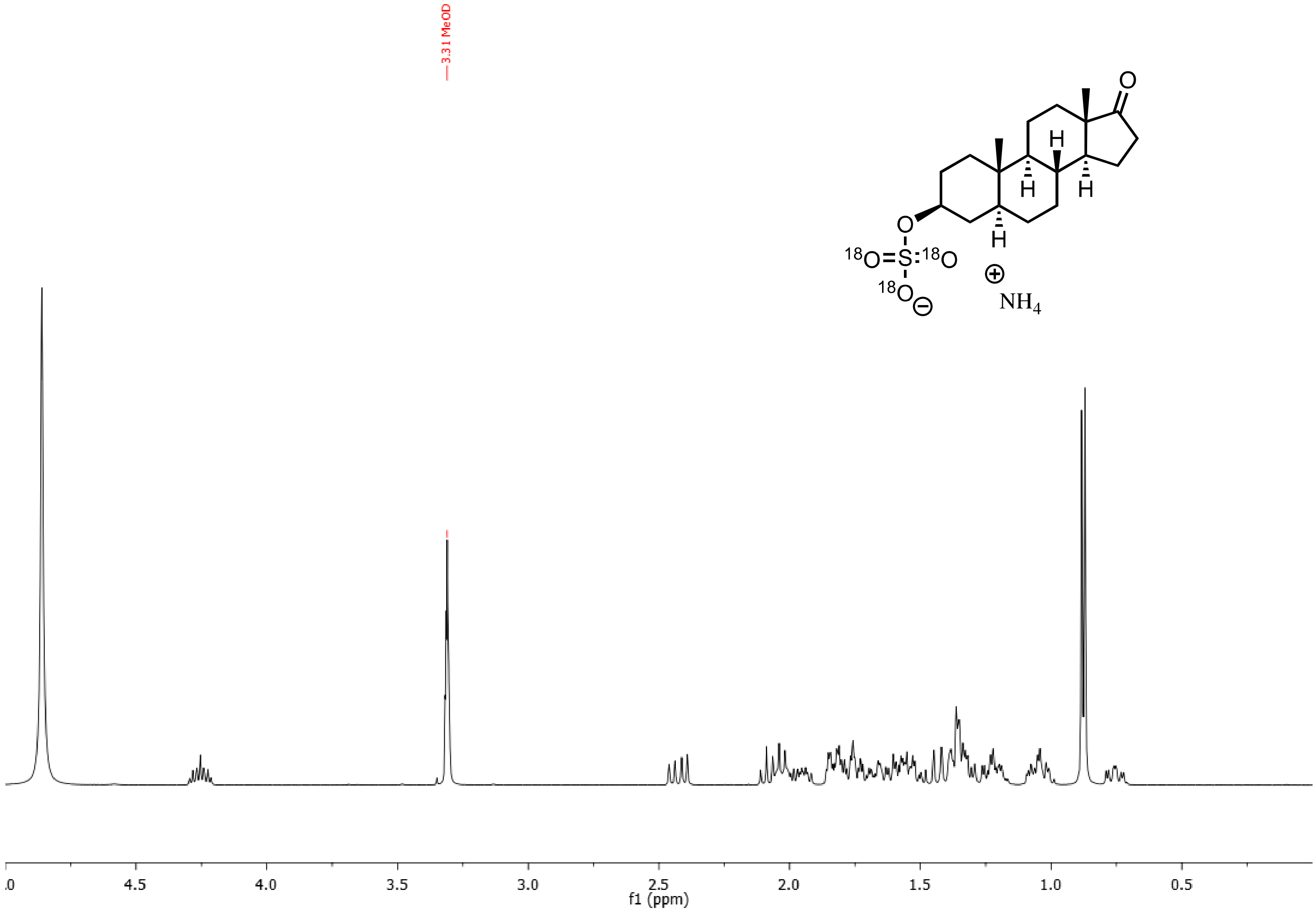

Epiandrosterone 3[$^{18}O_3$]-sulfate, ammonium salt (S3) ^{13}C NMR 101 MHz, CD_3OD

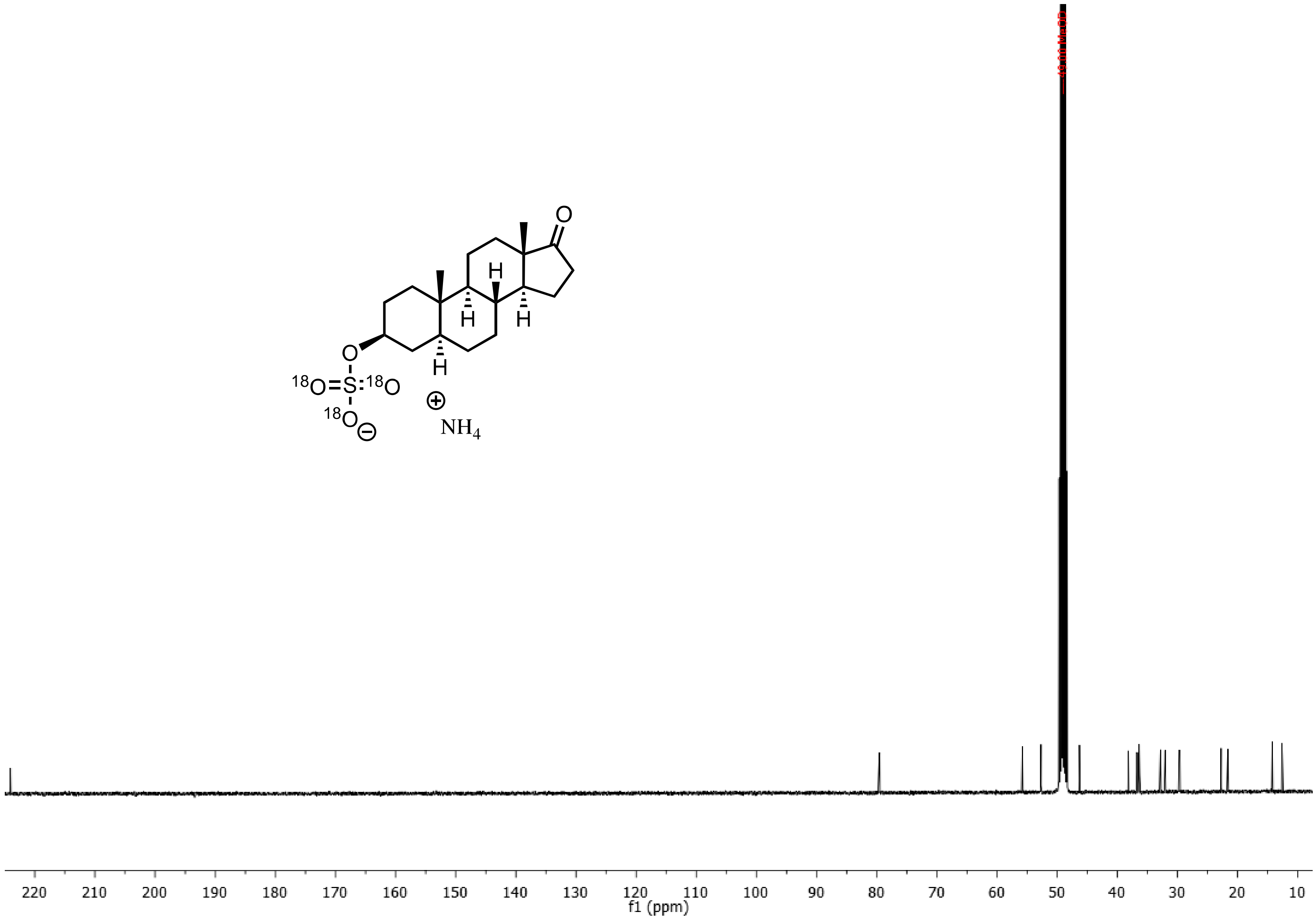

Testosterone-17-sulfate, ammonium salt (1) LRMS

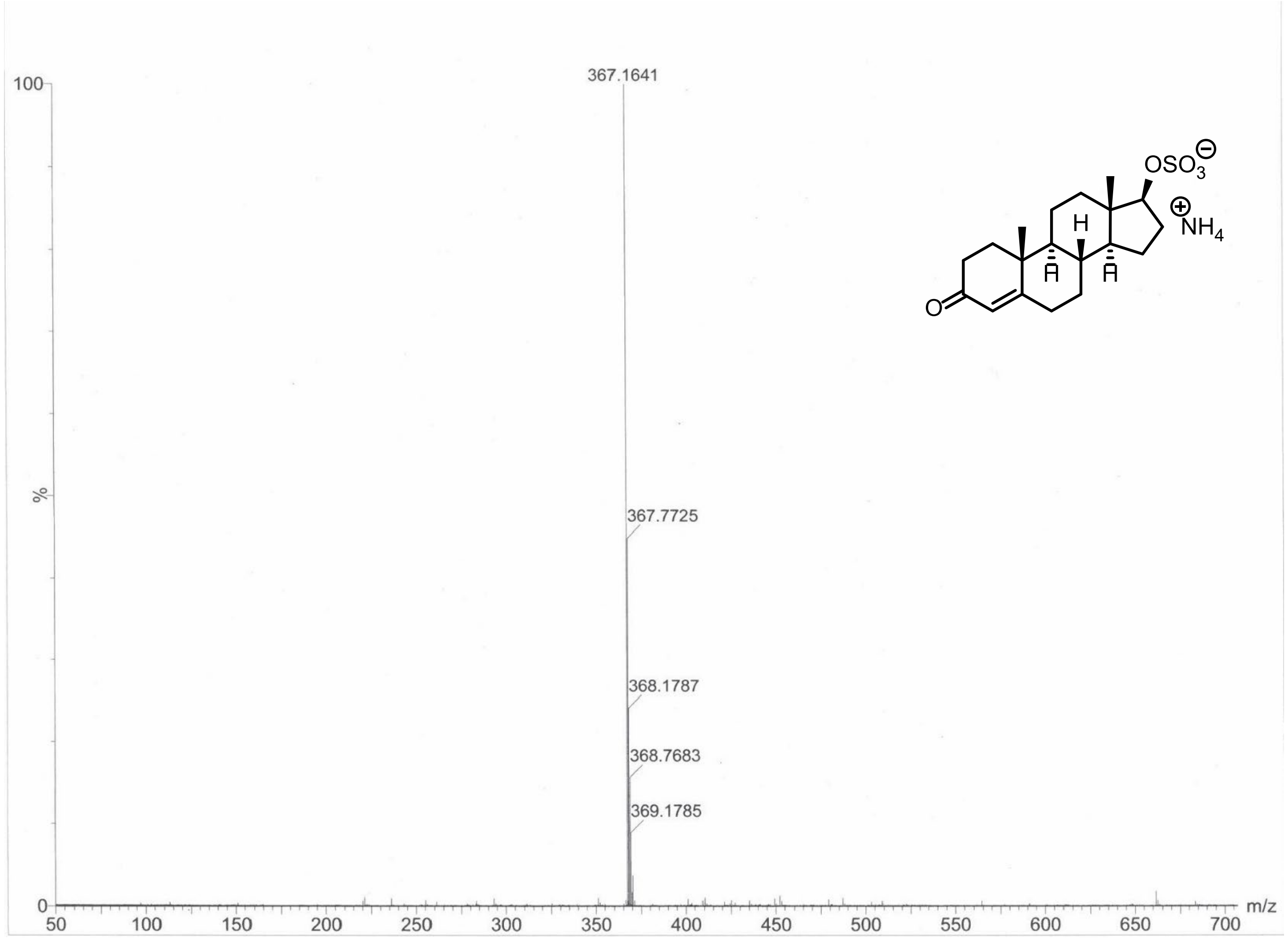

Testosterone-17-sulfate, ammonium salt (1) ^{1}H NMR 400 MHz, CD_3OD

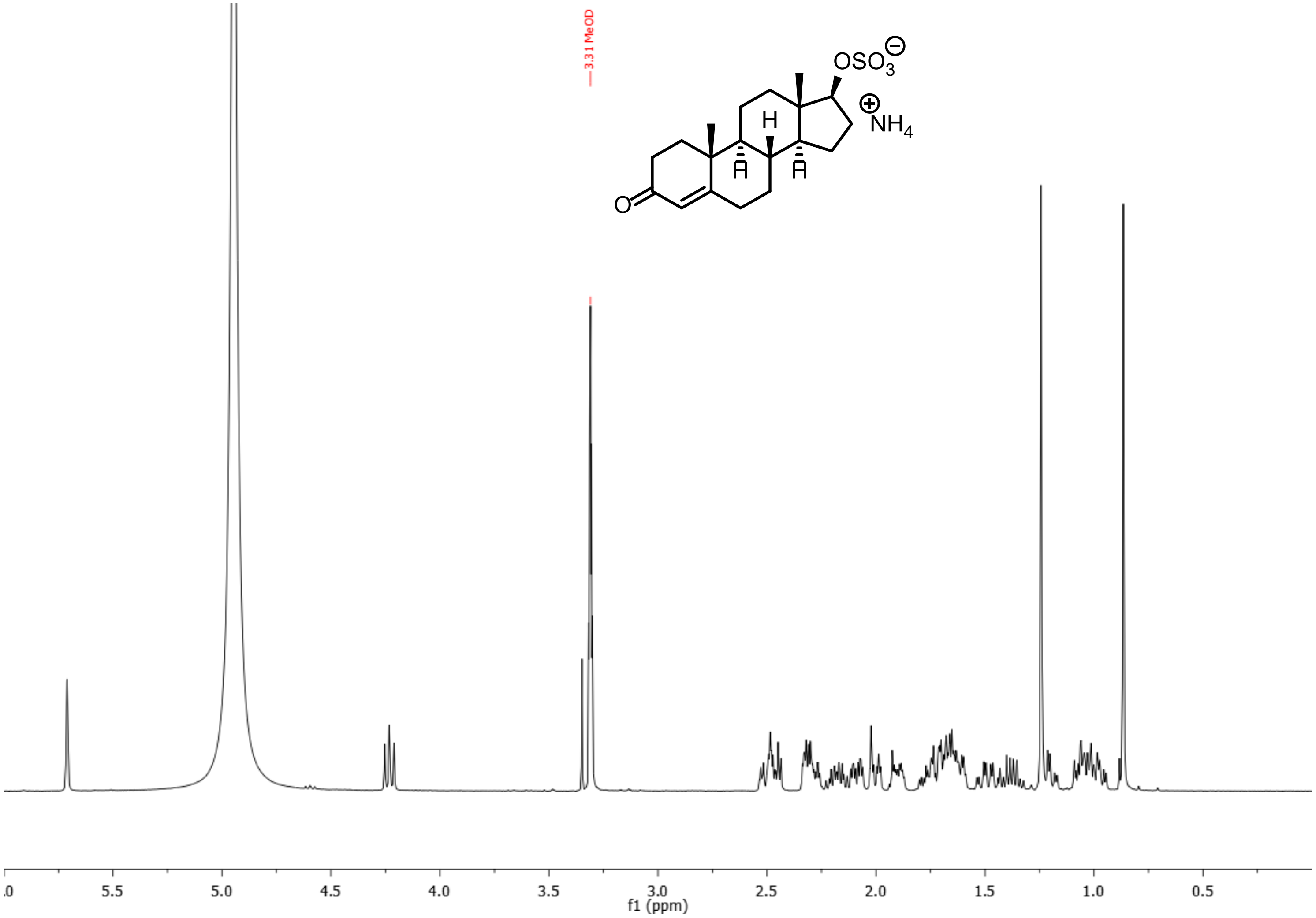

Epiandrosterone 3-sulfate, ammonium salt (2) LRMS

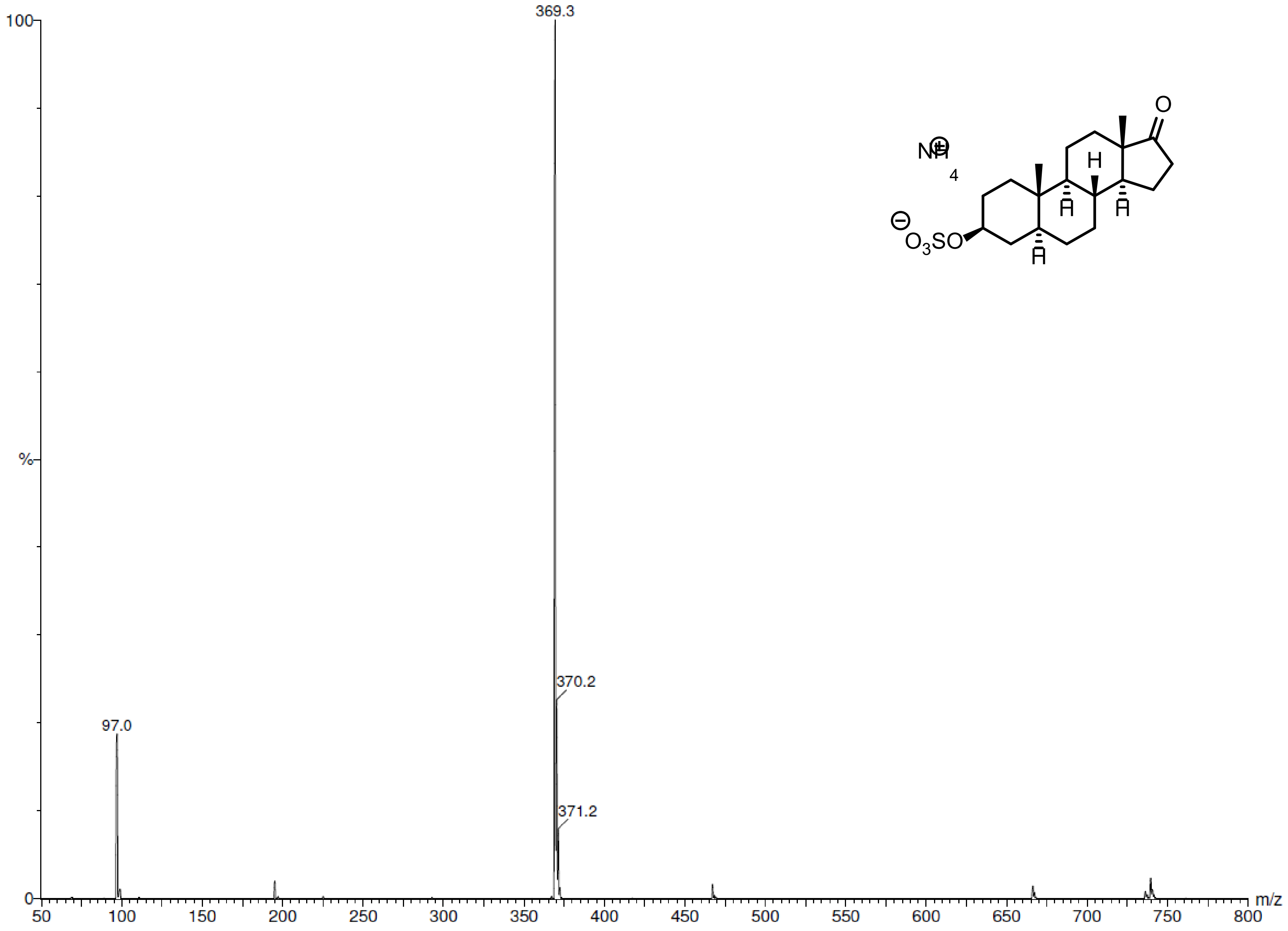

Epiandrosterone 3-sulfate, ammonium salt (2) ^{1}H NMR 400 MHz, CD_3OD

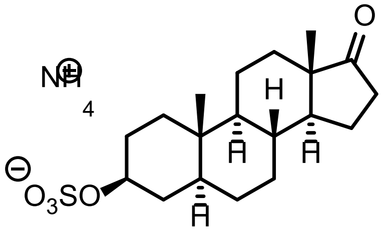

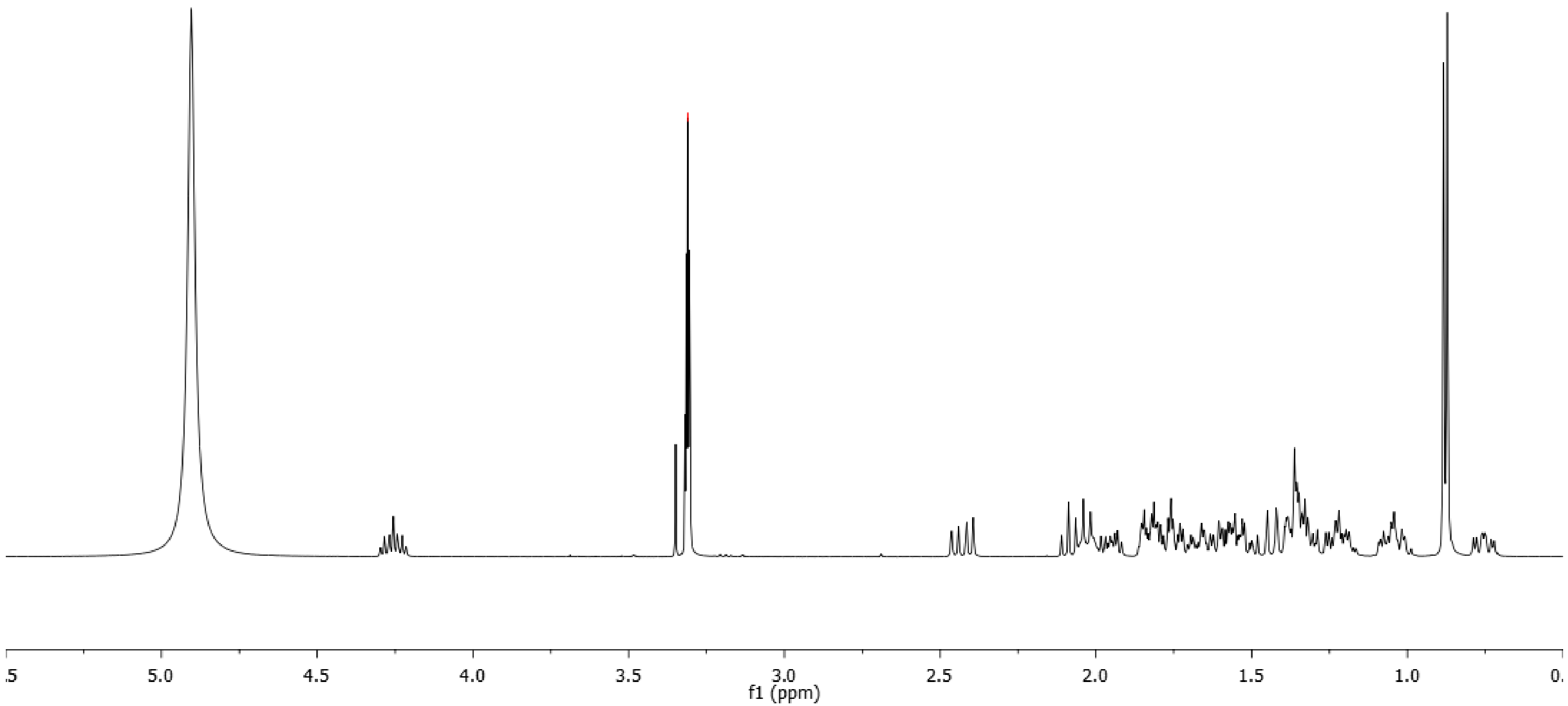

5α-Androstane-3β,17β-diol 3-sulfate, ammonium salt (S5) LRMS

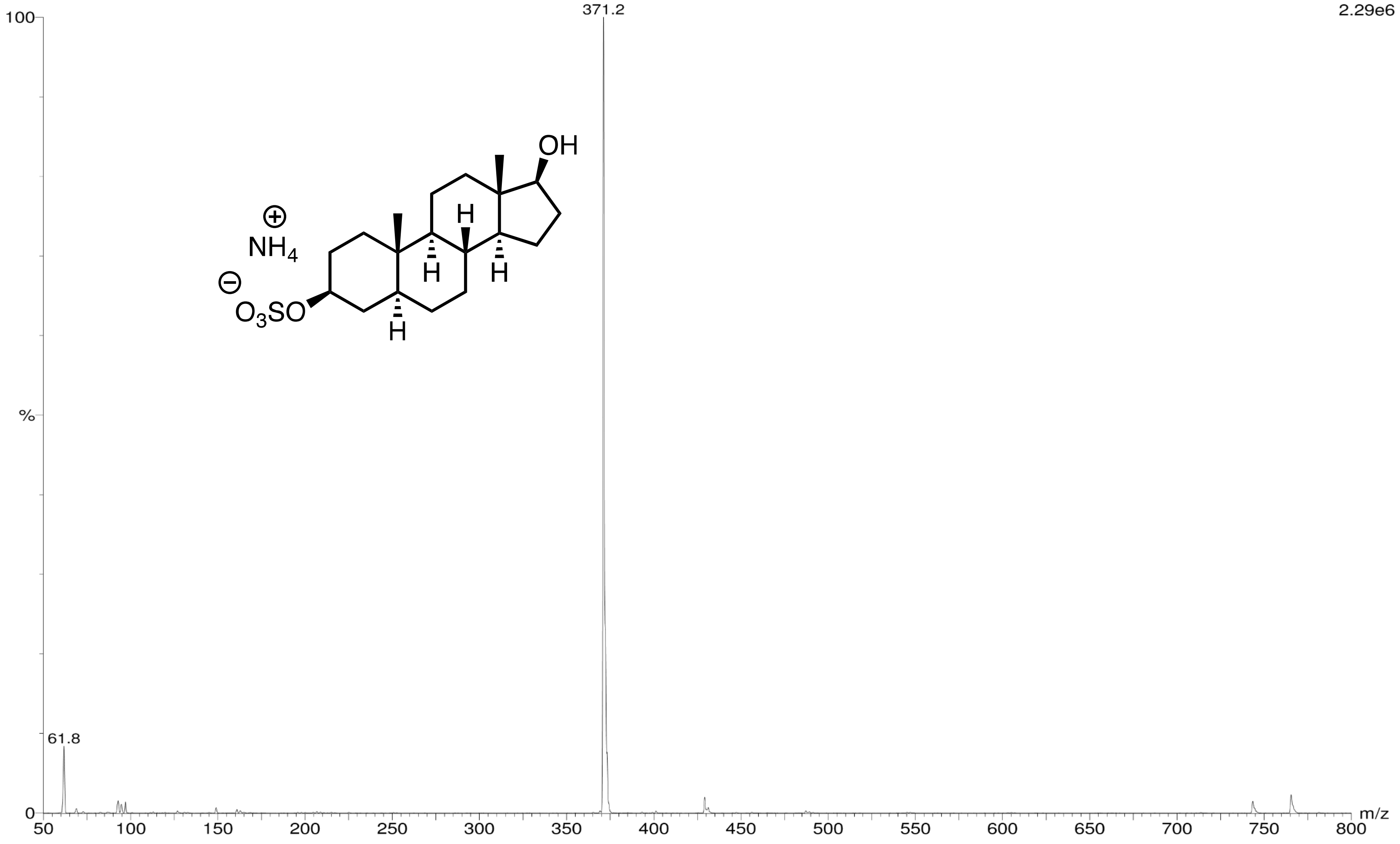

5α-Androstane-3β,17β-diol 3-sulfate, ammonium salt (S5) ^{1}H NMR 400 MHz, CD_3OD

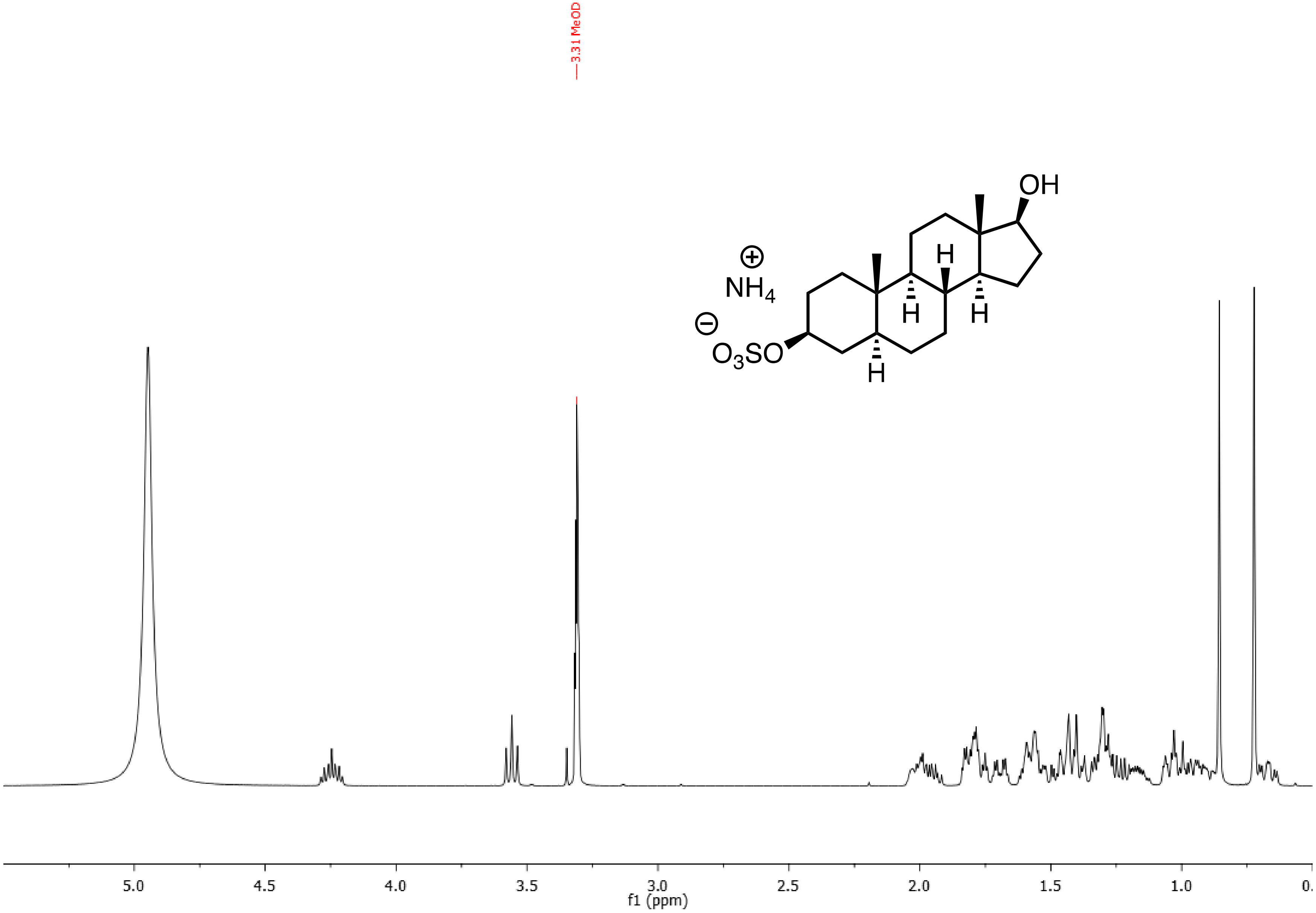

5α-Androstane-3β,17β-diol 3-sulfate, ammonium salt (S5) ^{13}C NMR 101 MHz, CD_3OD

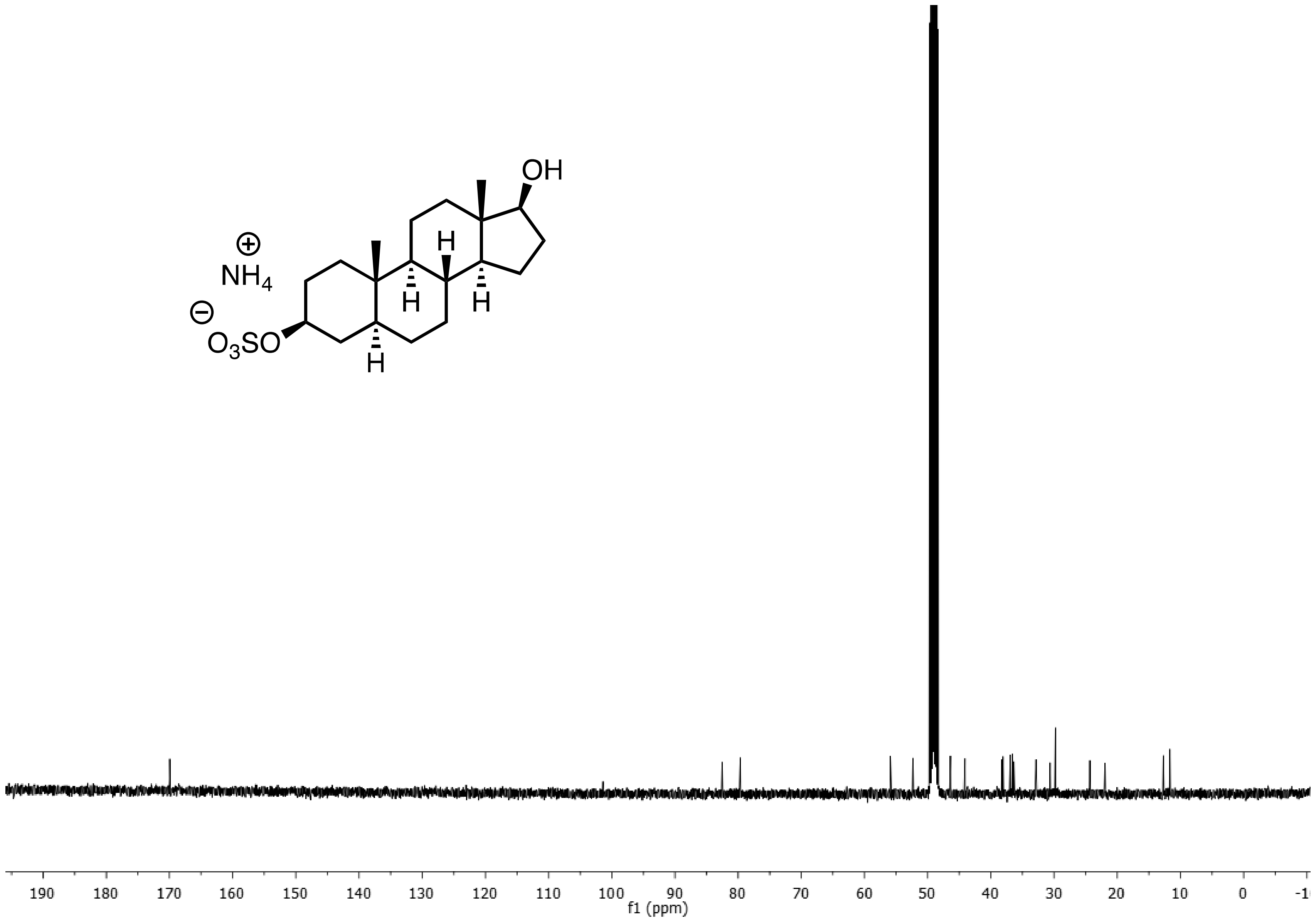

5α-Androstane-3β,17β[$^{18}O_3$]-diol bis(sulfate) (S4) LRMS

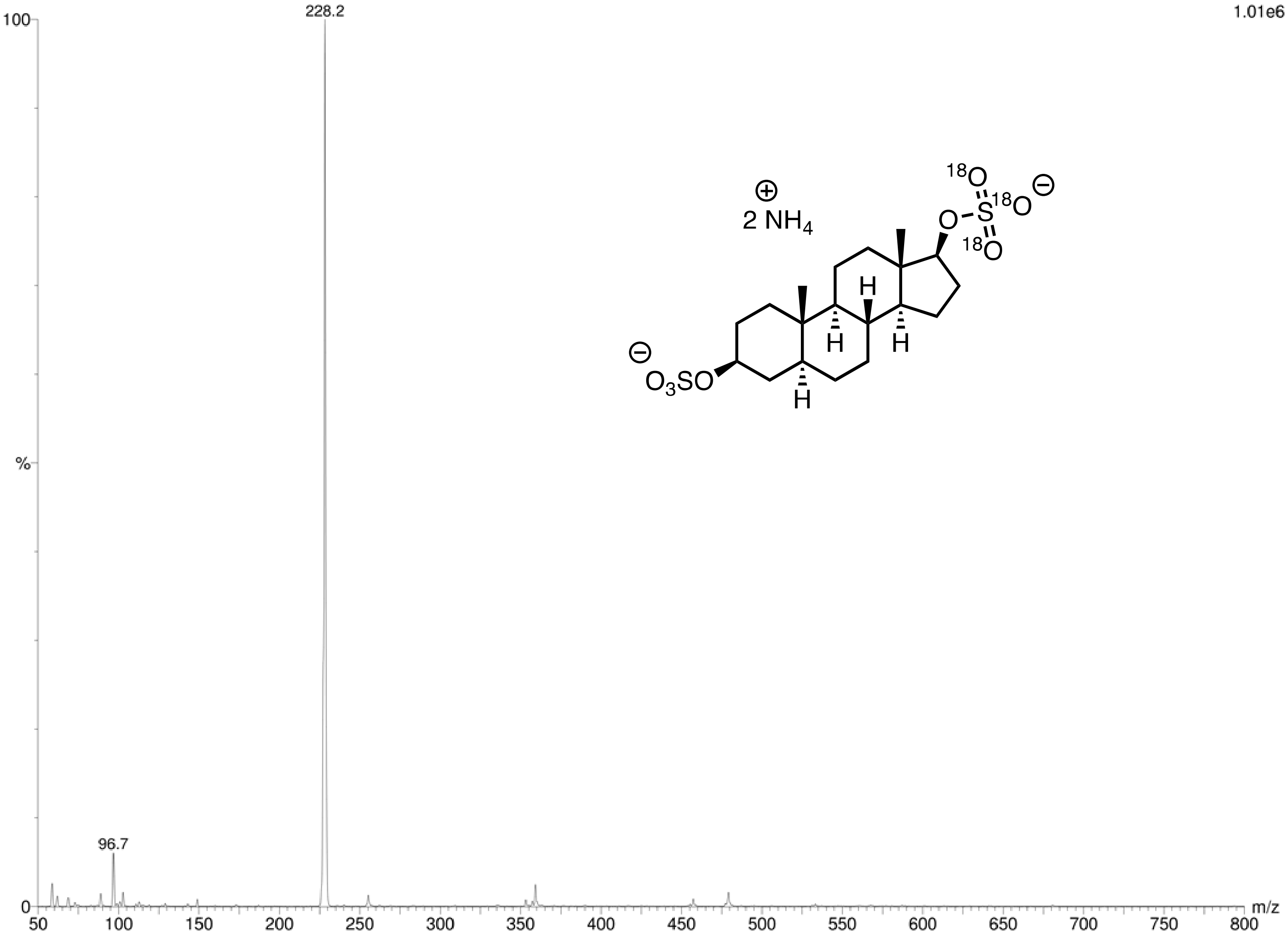

5α-Androstane-3β,17β[$^{18}O_3$]-diol bis(sulfate) (S4) ^1H NMR 400 MHz, CD_3OD

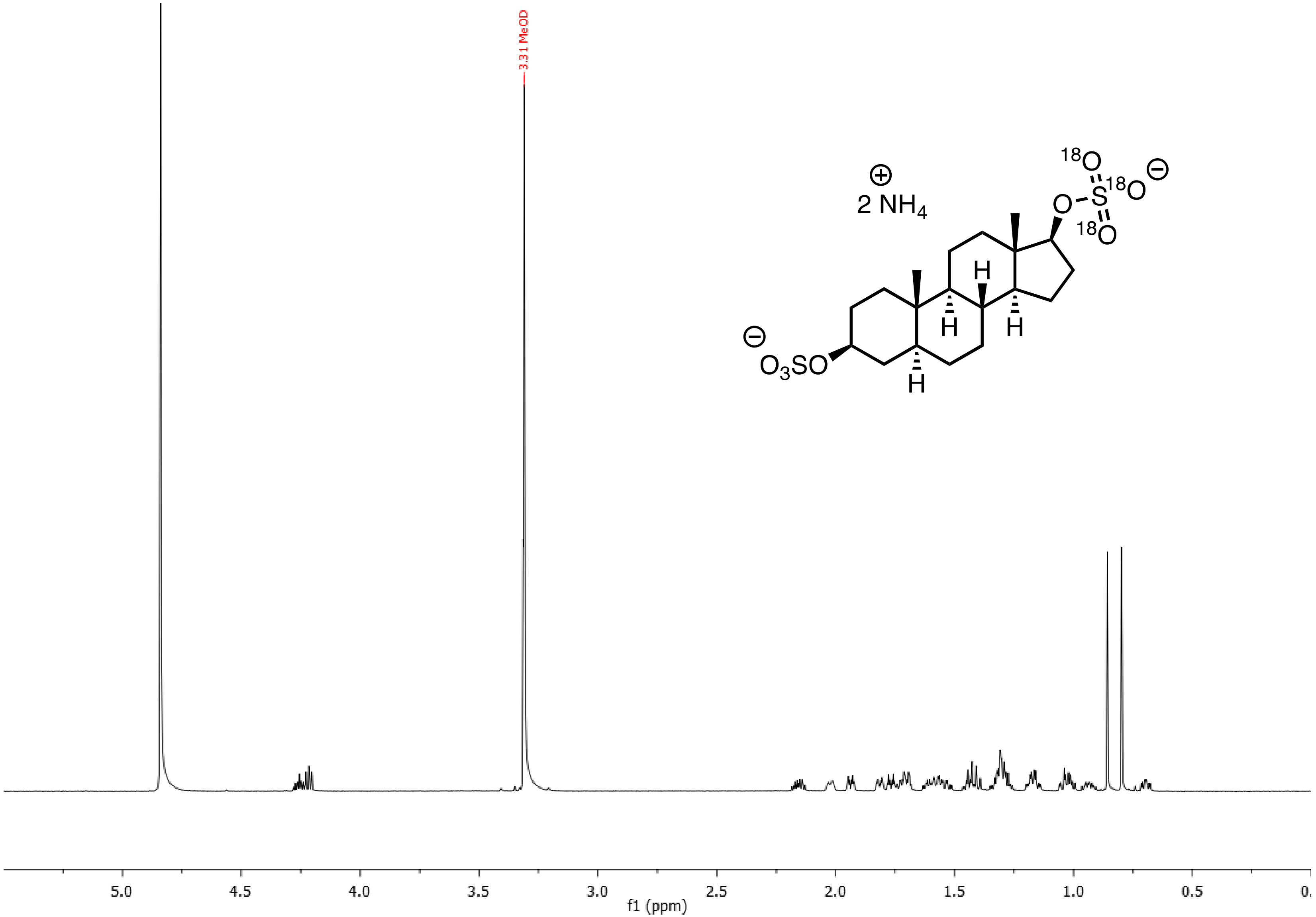

5α-Androstane-3β,17β[[18]O3]-diol bis(sulfate) (S4) ^{13}C NMR 151 MHz, CD3OD

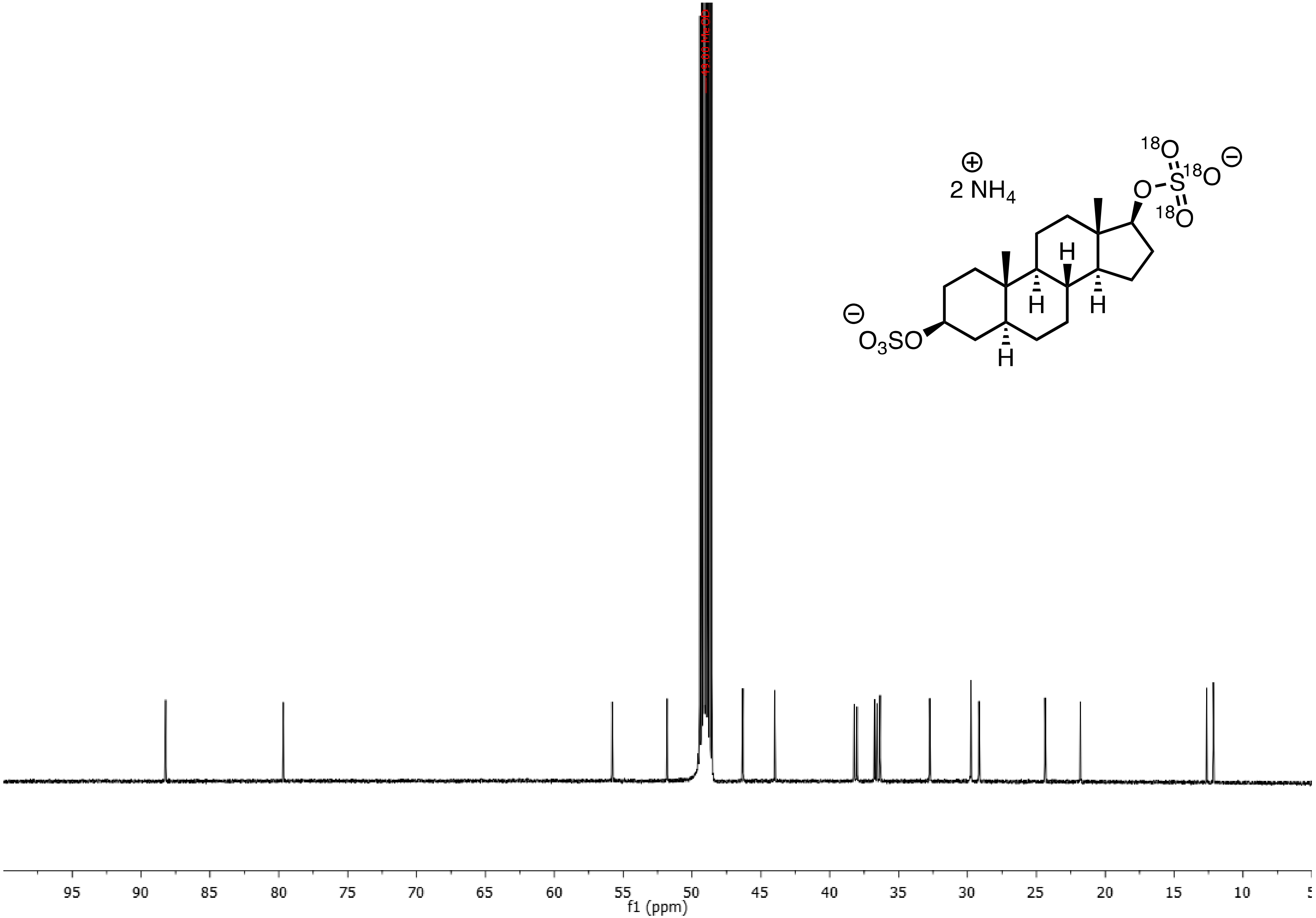

17α-Acetoxy-5α-androstane-3-one (S6) ¹H NMR 400 MHz, $CDCl_3$

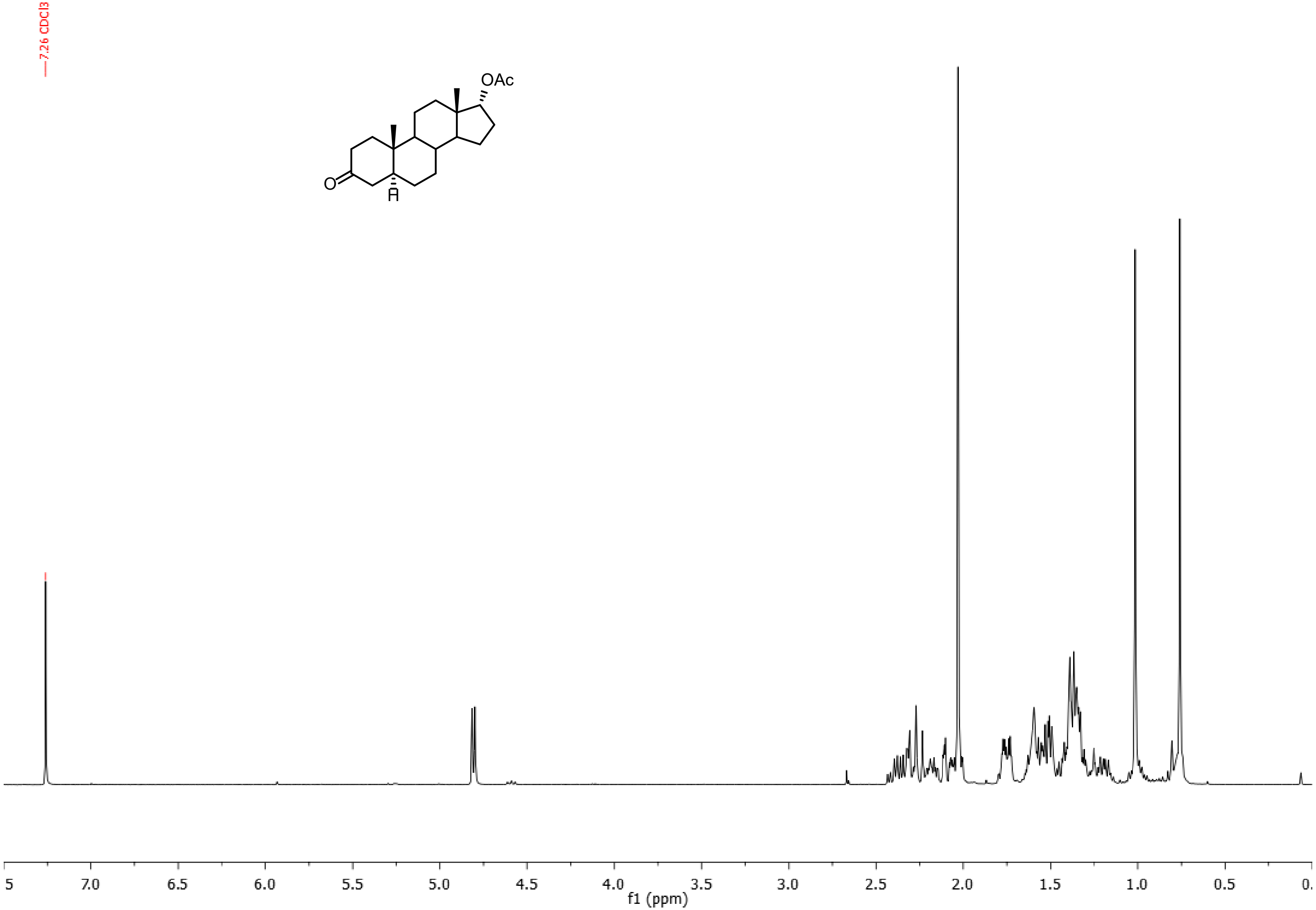

17α-Acetoxy-5α-androstane-3-one (S6) ^{13}C NMR 101 MHz, $CDCl_3$

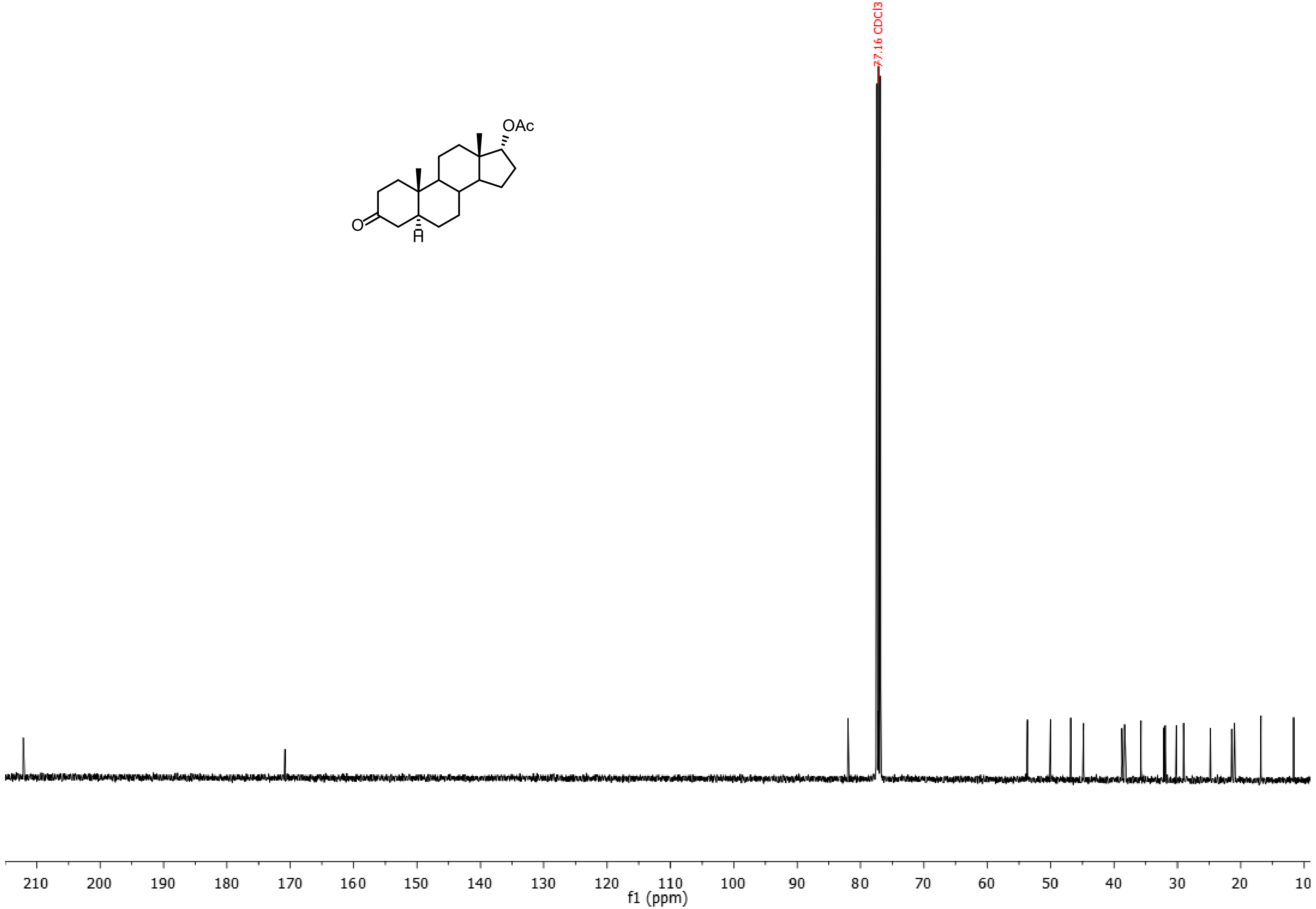

17α-Acetoxy-5α-androstane-3-one (S6) LRMS

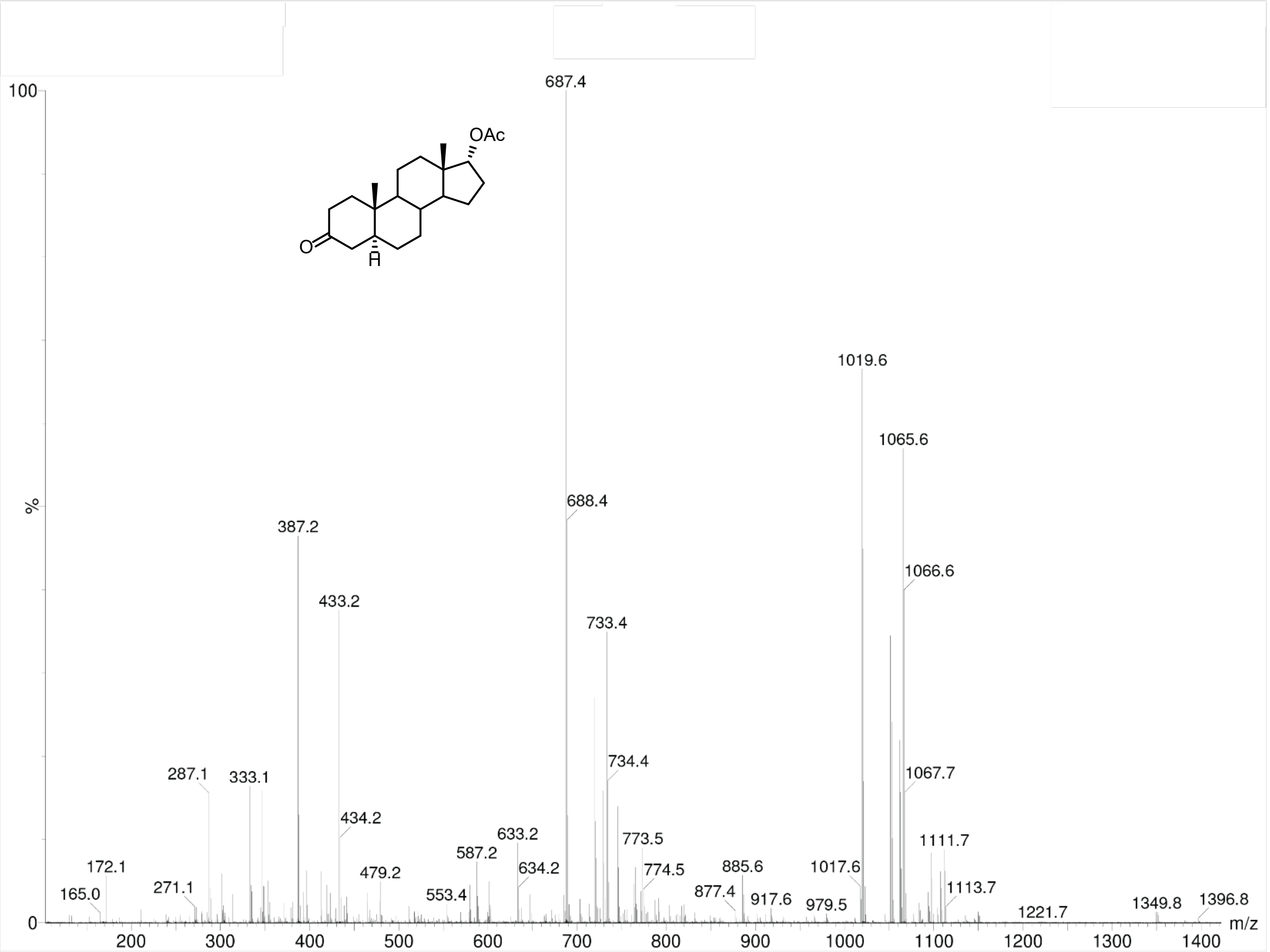

17α-Acetoxy-5α-androstane-3β-sulfate, ammonium salt (S7)^{1}H NMR 400 MHz, CD3OD

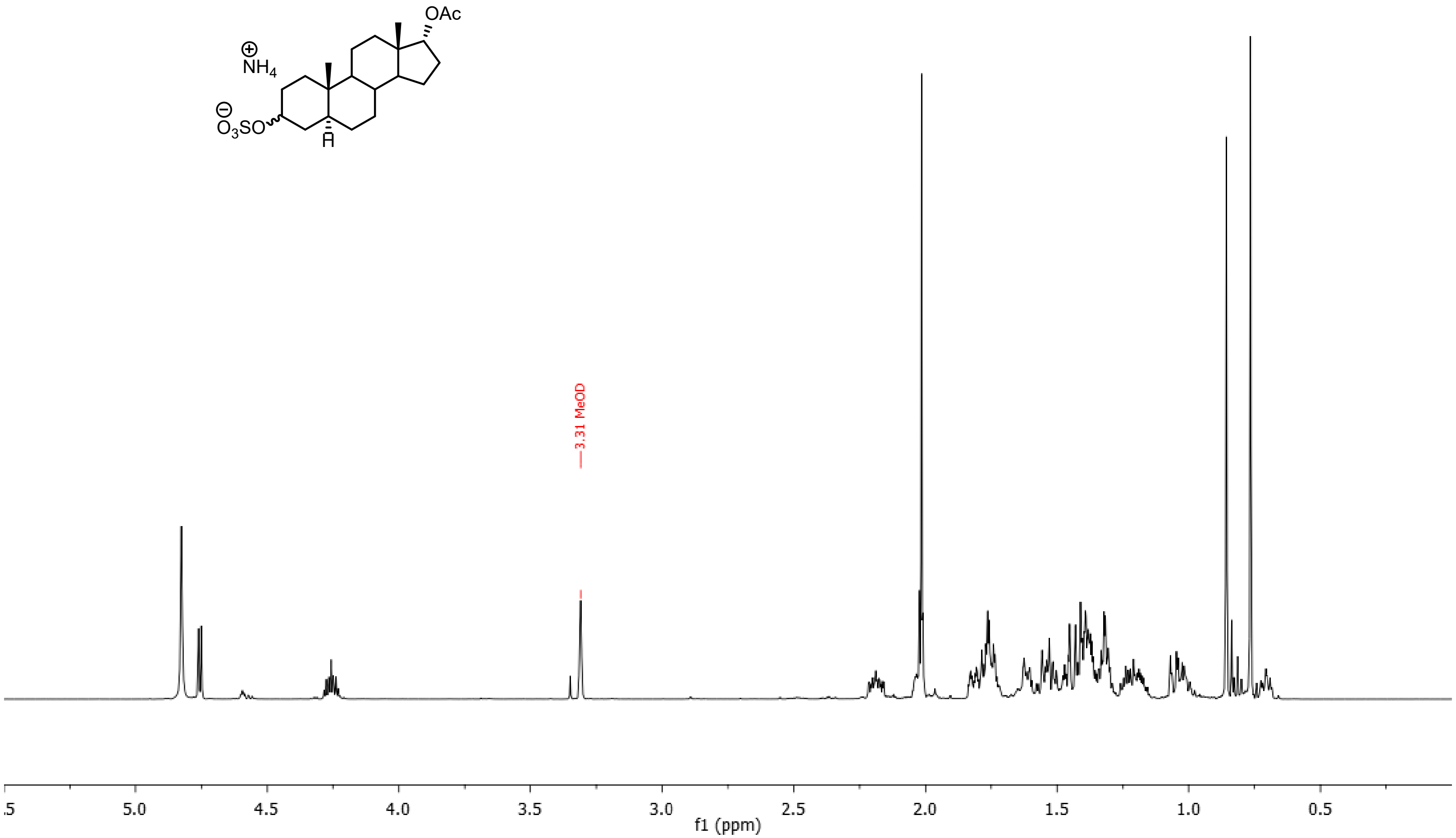

17α-Acetoxy-5α-androstane-3β-sulfate, ammonium salt (S7) ^{13}C NMR 101 MHz, CD_3OD

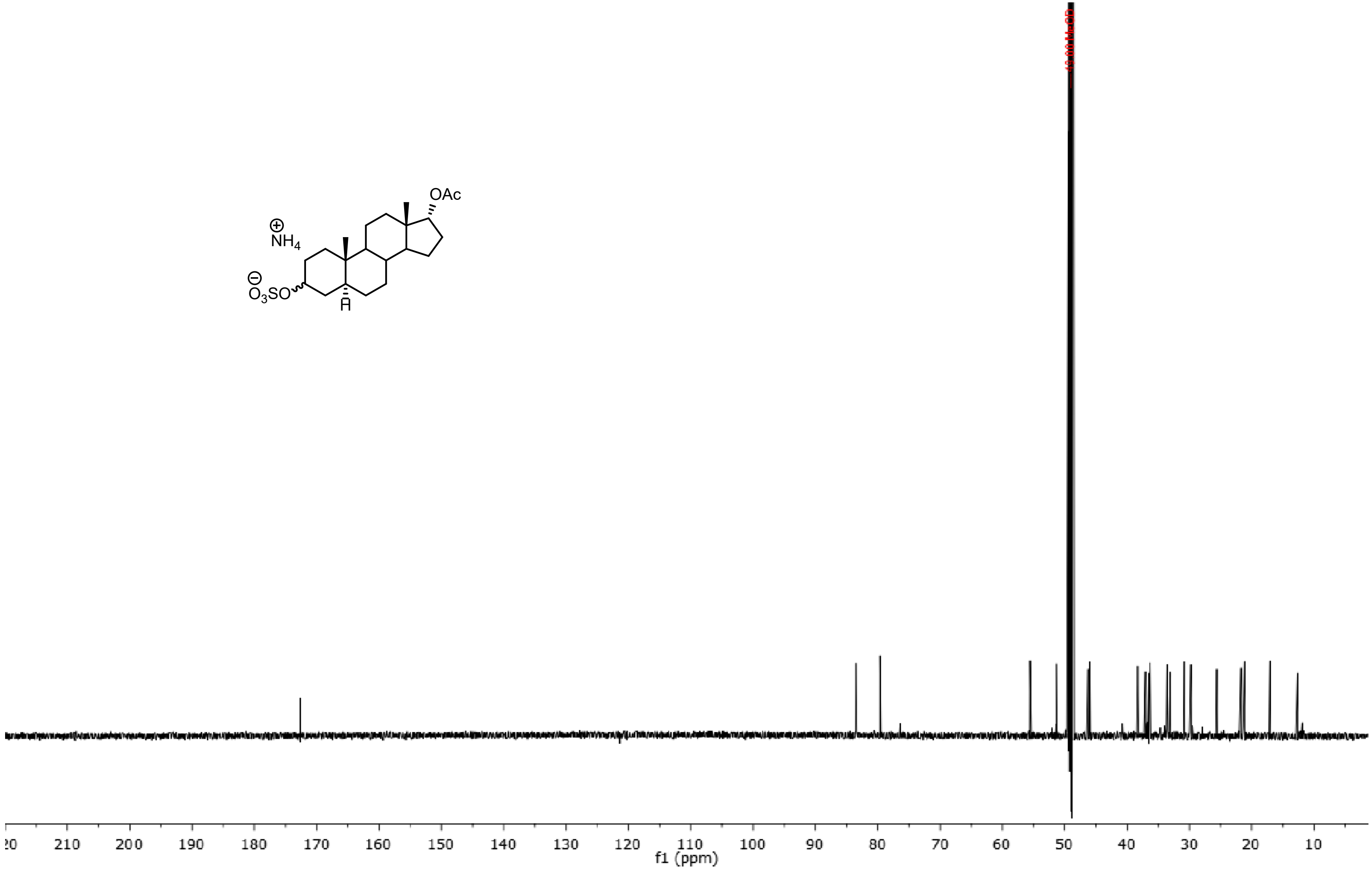

17α-Acetoxy-5α-androstane-3β-sulfate, ammonium salt (S7) LRMS

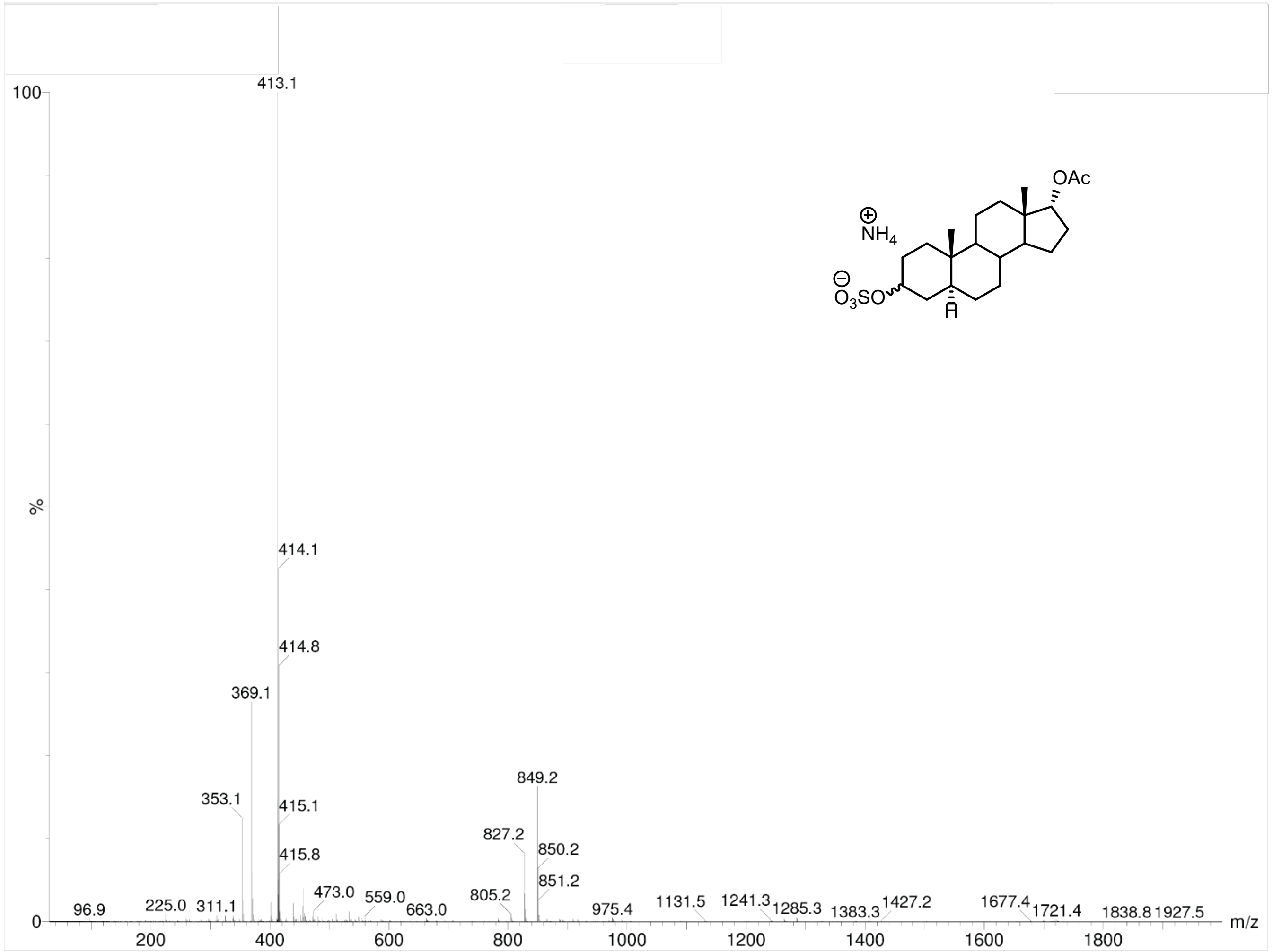

5α-Androstane-3β-17α-diol 17-sulfate, ammonium salt (3)^{1}H NMR 400 MHz, CD_3OD

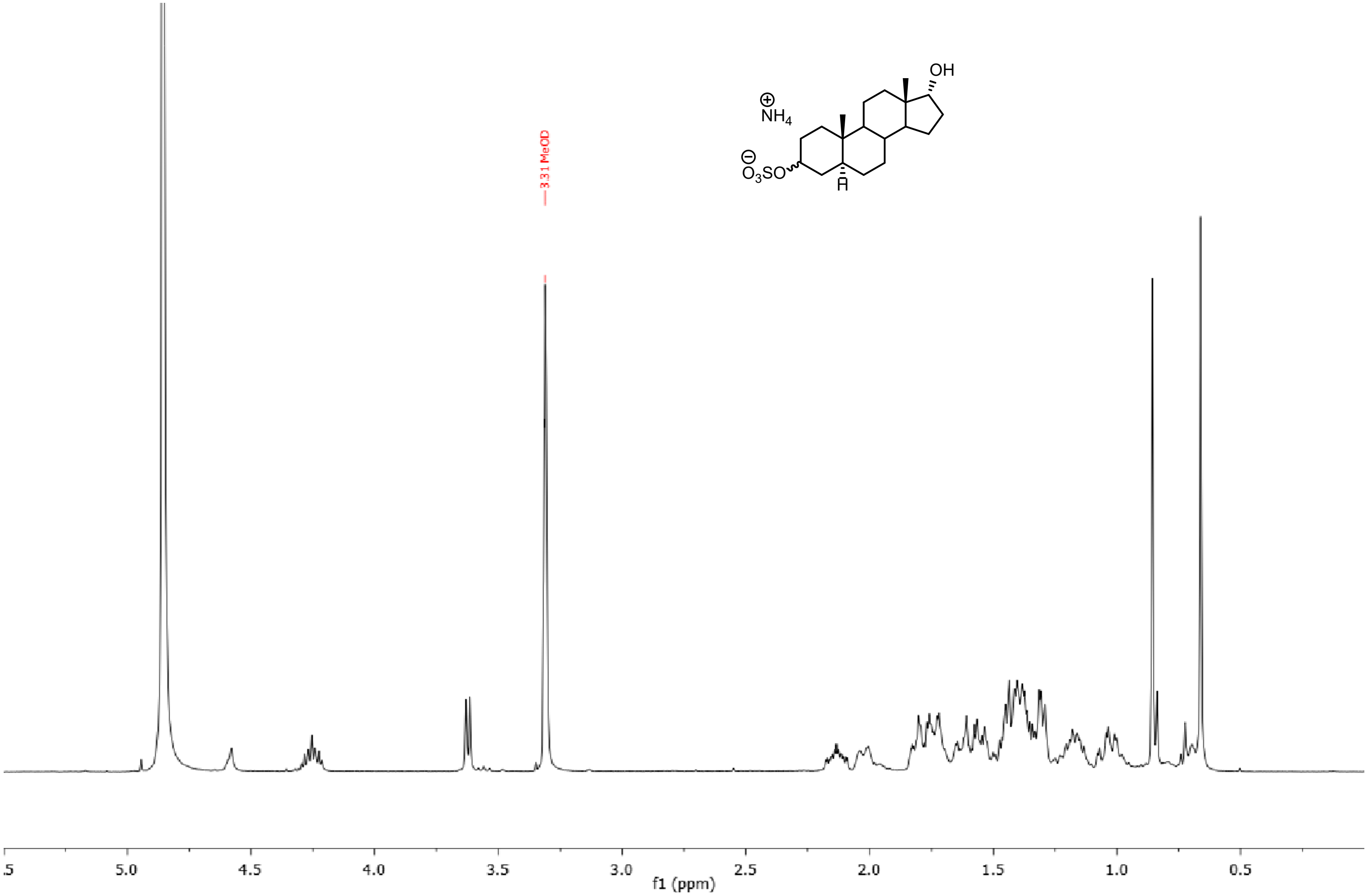

5α-Androstane-3β-17α-diol 17-sulfate, ammonium salt (3) ^{13}C NMR 101 MHz, CD_3OD

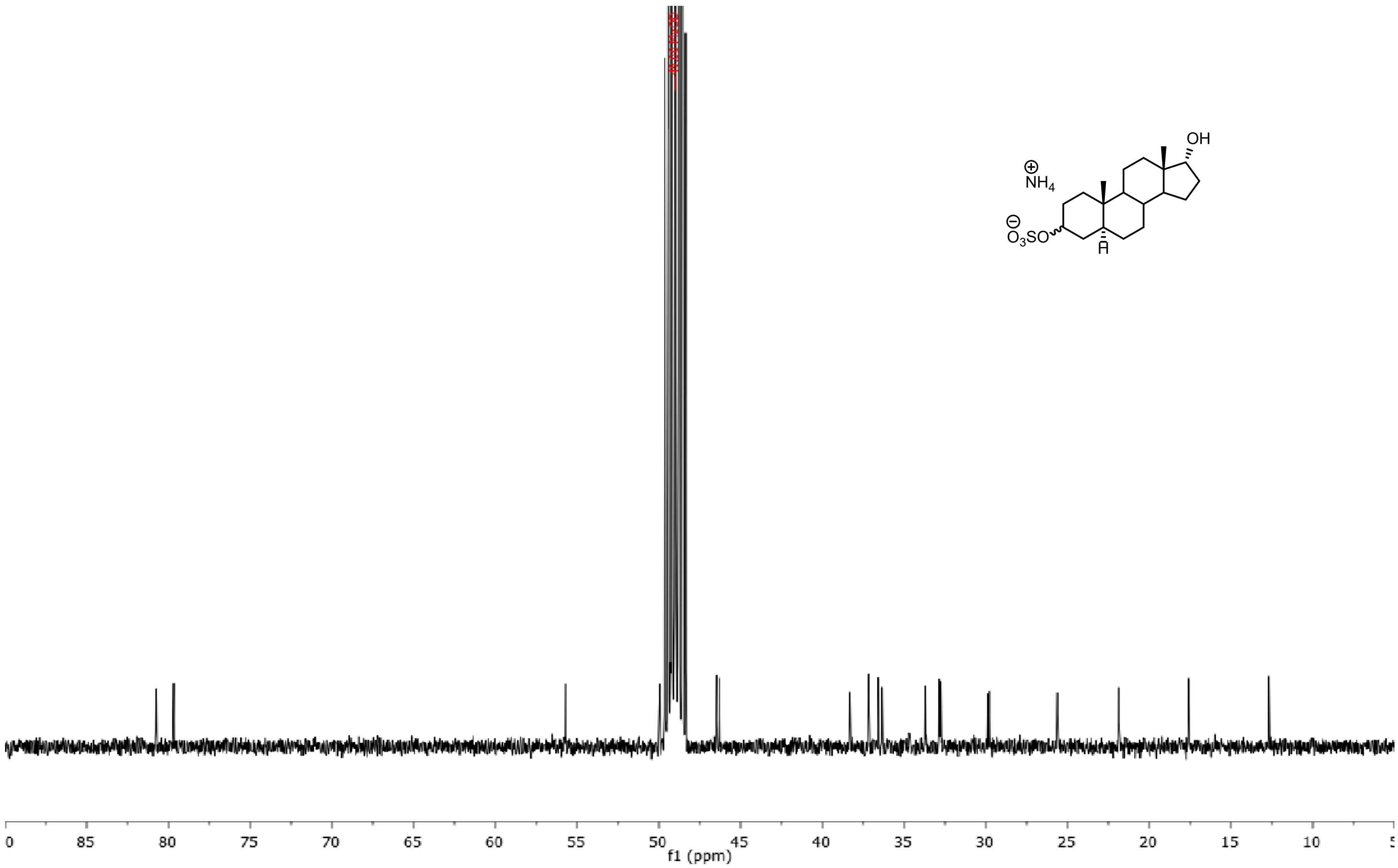

5α-Androstane-3β-17α-diol 17-sulfate, ammonium salt (3) LRMS

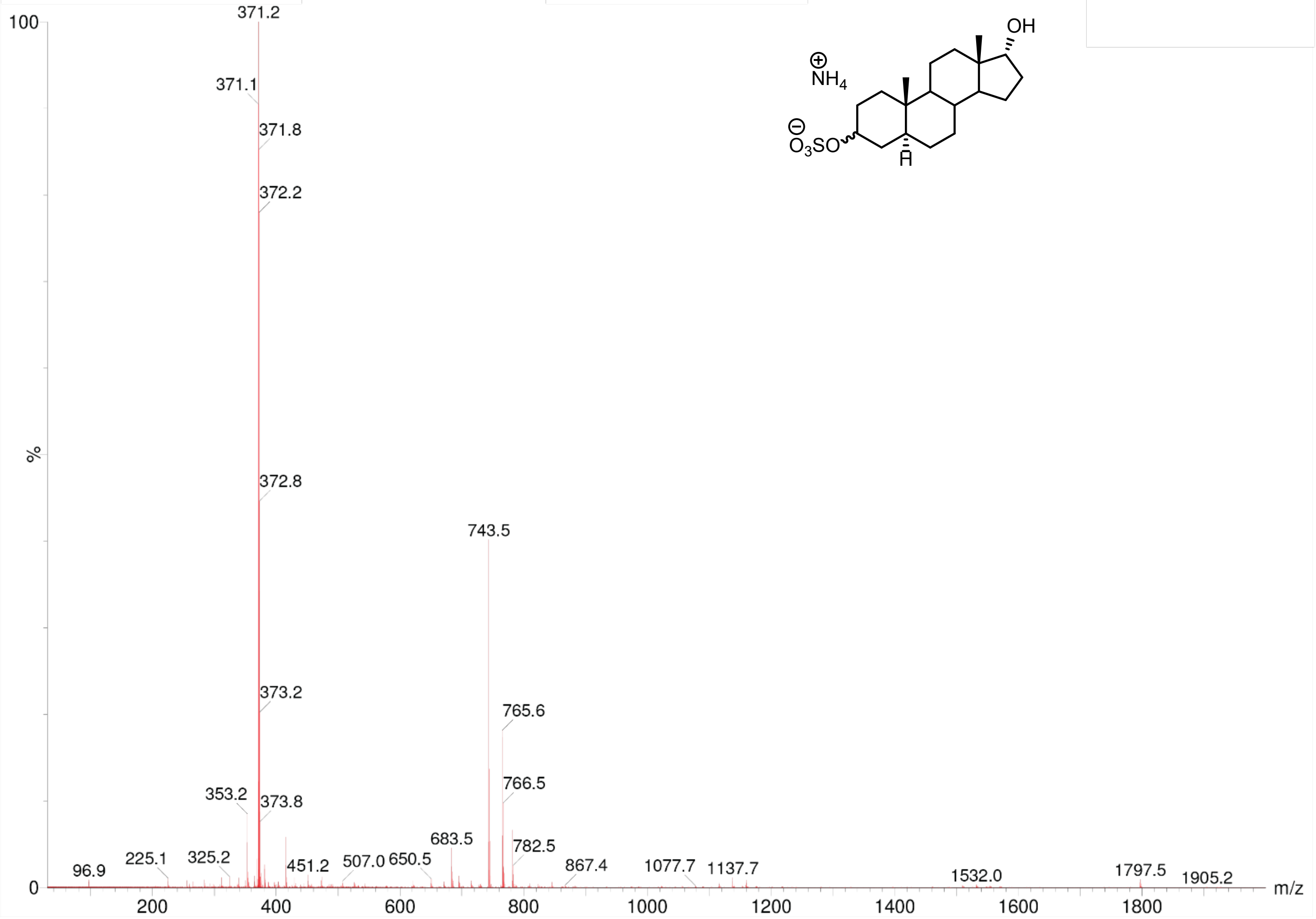

S.7 Script based data analysis

Example of R script for performing high throughput differential metabolite level analysis.

```
# Profiling urinary sulfate metabolites R analysis
#
# Written by Christopher Fitzgerald, PhD Candidate, & Dr. Teresa Neeman,
statistical consultant, Australian National University.
#
# This is a simple R script for processing LC-MS/MS data that has appended
sulfate ion data from spectrum analyser script.
#
# There are two main sections to the script.
# Firstly the data undergoes k-means clustering, and is annotated with a 1
(non-sulfate) or 2 (sulfate).
# Next, the data undergoes high throughput differential metabolite level
analysis. This is performed in the limma & Glimma packages from Bio-conductor.
# The data is then displayed as a volcano plot.

#______________________________________________________________________________________
#Packages used in this script
library(readxl)
library(ggplot2)
library(limma)
library(Glimma)
library(lattice)
library(factoextra)
library(ggfortify)
library(dplyr)
library(wrapr)

#_________________________________________________________________________________
#setting up directory
setwd()
#data is imported as an excel file and named
data.all<-read_excel()
data.all[is.na(data.all)] <- 0
str(data.all)
#Rows of data: define data bounds
data.all<-data.all[,]
names(data.all)
#subset normalised intensity data
data1<-subset(data.all,select = #enter columns that have your intensity data)
#keep row_id appended
row.names(data1)<-factor(data.all$row_id)

#_________________________________________________________________________________
# Scale zeros in data
# all zero values are given a number value, this is well under the value of an
actual vaule.
# Data is assessed manually and given an arbituary number to be scaled to.
data1[data1==0]<-1000

# Multidimensional scaling (MDS) Plot;
# allows a visual of the data spread "the plot is just a two dimensional space
to arrange the points"
# "the distances among each pair of points correlates as best as possible to
the dissimilarity between those two samples" - Environmental Computing
```

```
boxplot(data1)
data2<-log2(data1)
boxplot(data2, las=2)
#look at overall similarity between samples
plotMDS(data2, col=rep(1:3, each=3))


#Look at Mean-Variance relationship
var1<-function(x) mean(var(x[1:3]), var(x[4:6]))
sum1<-data.frame(list(mean1=apply(data2,1,mean),
                      var1=apply(data2,1,var1)))
sum1$sd<-sqrt(sum1$var1)
ggplot(sum1,aes(x=mean1, y=var1))+geom_point()+
  geom_smooth()



#_________Average shilhouette
method______________________________________________

fviz_nbclust(Kmdata, kmeans, method = "silhouette")

# source: https://uc-r.github.io/kmeans_clustering Optimal number of clusters
in kmeans for the given data set.
# All data is checked for optimal clustering using the average silhouette.
Althought data can be clustered into more than two groupings
# based on further divisions of sulfate species.The interest of this study was
simply to group sulfate like features from non-sulfate features.
# The average shilhouette approach measures the quality of a clustering, that
is it determines how well each object lies within its cluster.
# It computes the average silhouette of observations for different values of
k. The optimal number of clusters k is the one that maximises the avearage
silhouette.
# over a range of possible values of k.
# Kaufman, L. and Rousseeuw, P.J., 2009. Finding groups in data: an
introduction to cluster analysis (Vol. 344). John Wiley & Sons.

#___________ k-means sorting of
data________________________________________________

colnames(data.all)
# put column numbers of all parameters you want to use in clustering,
# in this case all sulfate ion transistions, IR and MA, were selected for as
clustering parameters.

Kmdata <- data.all[,c(".SO3-_normalisedIntensity","HSO3-_normalisedIntensity",
                      ".SO4-_normalisedIntensity","HSO4-_normalisedIntensity",
                      "NeutralLoss_SO3_normalisedIntensity","NeutralLoss_H2SO4_normalisedI
                      "Ratio","Max Count")]
(Kmd <- kmeans(Kmdata,2))
data.all$cluster=as.character(Kmd$cluster)
plot(Kmdata, col = data.all$cluster)
points(data.all$centers, col = 1:2, pch = 8, cex = 2)
```

```
#Check there is a new column called 'cluster'# and that the data has been
grouped into sulfates and non-sulfates.
names(data.all)
str(Kmd)

#__________Scatter plot with Kmeans clustering_____________________

# this clusters the data based on the inputted parameters below into two
groups.
# The grouping with the higher average IR and MA are assigned Sulfate and
correspond to grouping "2" in the cluster column.

Kmeansplot<-ggplot(data=data.all, aes(data.all$`Max
Count`,data.all$`Ratio`,color = factor(cluster)))+
  geom_point()+
  scale_color_manual(name="Compound type", values=c("black","orange"), labels
= c('Non-Sulfate','Sulfate'))+
  labs(x= "Maximum Abundance (MA)", y = "Intensity Ratio (IR)")+
  ggtitle("")+
  theme(plot.title = element_text(hjust = 0.5)) + theme_bw() +
  theme(panel.grid.major = element_blank(), panel.grid.minor =
element_blank())+
  theme_bw() + theme(panel.border = element_blank(),
                     panel.grid.major = element_blank(),
                     panel.grid.minor = element_blank(),
                     axis.line = element_line(colour = "black"))
print (Kmeansplot)

#___________Descriptive data for sulfates and
nonsulfates__________________________________________

# This was used to generate statistics that are shown in Table S.2 of the SI.

Sum <- data.all %>% group_by(cluster) %>%
  select(`Max Count`,Ratio) %>%
  summarise_all(list(mean = mean,
                     median = median,
                     std = sd,
                     min = min,
                     max = max))


#Interquartile range for sulfates and nonsulfates
tbl_MA <- data.all %>%  group_by(cluster) %>%
  summarise(SD = sd(`Max Count`),
            Mean = mean(`Max Count`),
            Median = as.numeric(median(`Max Count`)),
            "Trimmed Mean" = mean(`Max Count`, trim = 0.2),
            "Geometric Mean" = psych::geometric.mean(`Max Count`),
            "Harmonic Mean" = psych::harmonic.mean(`Max Count`),
            IQR = IQR(`Max Count`),
            "%25 Q" = quantile(`Max Count`, .25),
            "%50 Q" = quantile(`Max Count`, .5),
```

```
            "%75 Q" = quantile(`Max Count`, .75))

tbl_IR <- data.all %>%  group_by(cluster) %>%
  summarise(SD = sd(`Ratio`),
            Mean = mean(`Ratio`),
            Median = as.numeric(median(`Ratio`)),
            "Trimmed Mean" = mean(`Ratio`, trim = 0.2),
            "Geometric Mean" = psych::geometric.mean(`Ratio`),
            "Harmonic Mean" = psych::harmonic.mean(`Ratio`),
            IQR = IQR(`Ratio`),
            "%25 Q" = quantile(`Ratio`, .25),
            "%50 Q" = quantile(`Ratio`, .5),
            "%75 Q" = quantile(`Ratio`, .75))


#__________Box Plot of k-means clustering data____________

# Max count Product Ion abundance
ggplot(data.all, aes(x= data.all$cluster, y=data.all$`Max Count`,
fill=data.all$cluster)) +
  geom_violin(width=0.75, trim = TRUE) +
  geom_boxplot(width=0.1, alpha = .1)  +
  theme(legend.position="none",
        plot.title = element_text(size=11)) +
  ggtitle("Product Ion Abundance") +
  ylab ("Maximum Abundance (%)")+
  xlab("") +
  scale_x_discrete(breaks=c("1","2"),
                   labels=c("Non-Sulfate\nn=2333", "Sulfate\nn=911")) +
  theme(plot.title = element_text(hjust = 0.5)) + theme_bw() +
  theme(panel.grid.major = element_blank(), panel.grid.minor =
element_blank())+
  theme_bw() + theme(panel.border = element_blank(),
                     panel.grid.minor = element_blank(),
                     axis.line = element_line(colour = "black"))+
  guides(fill=guide_legend(title=NULL))+
  scale_fill_manual(values=c("grey", "orange"),
                    name="Compound type",
                    breaks=c("1", "2"),
                    labels=c("Non-Sulfate", "Sulfate"))+
  coord_flip()


# Intensity Ratio box plots
ggplot(data.all, aes(x= data.all$cluster, y=data.all$Ratio,
fill=data.all$cluster)) +
  geom_violin(width = .75, trim = TRUE) +
  geom_boxplot(width=.1, alpha = .1)  +
  theme(legend.position="none",
        plot.title = element_text(size=11)) +
  ggtitle("Proportion of Sulfate Ions") +
  ylab ("Intensity Ratio (%)")+
  xlab("") +
  scale_x_discrete(breaks=c("1","2"),
```

```
                   labels=c("Non-Sulfate", "Sulfate")) +
  theme(plot.title = element_text(hjust = 0.5)) + theme_bw() +
  theme(panel.grid.major = element_blank(), panel.grid.minor =
element_blank())+
  theme_bw() + theme(panel.border = element_blank(),
                     panel.grid.minor = element_blank(),
                     axis.line = element_line(colour = "black"))+
  guides(fill=guide_legend(title=NULL))+
  scale_fill_manual(values=c("grey", "orange"),
                    name="Compound type",
                    breaks=c("1", "2"),
                    labels=c("Non-Sulfate", "Sulfate"))


#__________________ Limma analysis_________________________

# This was used to undergo high throughput differential metabolite level
analysis.

Group <- factor(rep(c(#set groupings here), each=3), levels = c(#set groupings
here))
design1 <- model.matrix(~0+Group)
colnames(design1)<-c(#set groupings here)

fit <- lmFit(data2, design=design1)

# Look at contrasts between groups
cont.matrix <- makeContrasts(#contrast data groupings. i.e., X-Y, X-Z, X-A,.
                             levels = design1)

fit1 <- contrasts.fit(fit, cont.matrix)
fit1 <- eBayes(fit1)
summa.fit1<-decideTests(fit1)
summary(summa.fit1)

names(fit1)

topTable(fit1, coef=1)
fit1$cluster <-data.all$cluster
fit1$p.value_log<--1*log10(fit1$p.value)
fit1$mz<-data.all$`m/z`

str(fit1)
names(fit1)

#_______________Volcano Plot__________________

# Make volcano plot for coef as defined above (design1)

results1 <- topTable(fit1, coef=1,number=#)

maintitle <- c(maint = "#treatment name", subt = "#treatment name sulfates
only")
```

```
# define bounds for volcano plot

Category <- (results1$adj.P.Val<0.01)*1 +
(abs(results1$logFC)>3)*(results1$adj.P.Val<0.01)*1
Category <- (Category,labels=c("not sig","sig, <8FC","sig,>8FC"))

# plot volcanoplot
VP <- ggplot(results1,aes(x=logFC, y=-1*log(adj.P.Val,10), colour=Category))+
  geom_point()+ scale_colour_manual(values = c("grey", "blue", "red"))
VP + labs (title = maintitle[1], x = expression("log"[2]*"(FC)"), y =
expression("log"[10]*"(adj.p-value)")) +
  theme(plot.title = element_text(hjust = 0.5)) + theme_bw() +
  theme(panel.grid.major = element_blank(), panel.grid.minor =
element_blank())+
  theme_bw() + theme(panel.border = element_blank(),
                     panel.grid.major = element_blank(),
                     panel.grid.minor = element_blank(),
                     axis.line = element_line(colour = "black"))

#_________________Volcano plot of just sulfates____________

#  This isolates features based their k-means clustering groupings. and plots
them as a volcanoplot.

data_cluster <- data.all[,c("row_id","cluster")]
results2 <- results1
results2$row_id<-row.names(results2)
results_cluster<-merge.data.frame(results2,data_cluster)

results_sulfate <-subset(results_cluster, cluster==2)

Category2<-(results_sulfate$adj.P.Val<0.01)*1 +
(abs(results_sulfate$logFC)>3)*(results_sulfate$adj.P.Val<0.01)*1
Category2<-factor(Category2,labels=c("not sig","sig, <8FC","sig,>8FC"))

VP <- ggplot(results_sulfate,aes(x=logFC, y=-1*log(adj.P.Val,10),
colour=Category2))+
  geom_point()+ scale_colour_manual(values = c("grey", "blue", "red"))

VP + labs (title = maintitle[2], x = expression("log"[2]*"(FC)"), y =
expression("log"[10]*"(adj.p-value)")) +
  theme(plot.title = element_text(hjust = 0.5)) + theme_bw() +
  theme(panel.grid.major = element_blank(), panel.grid.minor =
element_blank())+
  theme_bw() + theme(panel.border = element_blank(),
                     panel.grid.major = element_blank(),
                     panel.grid.minor = element_blank(),
                     axis.line = element_line(colour = "black"))
```

Python based Scripts

Spectrum analyser script

In [ ]:

```
# Spectrum Analyser
#
# Written by Dr Adam J. Carroll, RSB/RSC Joint Mass Spectrometry Facility, Australian N
ational University
#
# This is a simple python script for processing MS/MS spectra stored in Mascot Generic
 Format (.mgf) files to
# extract qualitative and quantitative information about evidence supporting fragmentat
ion mechanisms of interest
# and output the findings into a tab-delimited data matrix suitable for multivariate an
alysis.
#
# When executed, the script looks in the current working directory for .mgf files and p
rocesses them all, one-by-one,
# generating a report matrix for each .mgf file. The script parses the spectra in each
 .mgf and then looks for MS/MS
# signals with m/z values matching with user-settable reporter ions, ion losses and neu
tral losses.

# The customisable parameters may be set in the 'CUSTOMISABLE PARAMETERS' section just
 below (see explanations there).

#Import dependencies
import re, glob, os
from natsort import natsorted

#Set constants
PROTON_MASS = 1.00727647

### CUSTOMISABLE PARAMETERS ###

# If the .mgf files to process are in a different folder than the script, uncomment the
line below and set the full path to the folder containing the .mgf files
os.chdir('')
# The charge state to assign if no charge state is associated with the parent ion in th
e .mgf file
defaultCharge = -1

# The minimium signal intensity (in counts) required for a signal to be reported as a r
eporter ion signal
absoluteReporterIonIntensityThreshold = 0

# The minimum relative signal intensity (as % of base peak intensity) required for a si
gnal to be reported as a neutral loss signal
neutralLossTransitionRelativeIntensityThreshold = 5

# The annotations and m/z values of reporter ion signals to look for and report on
reporterIons = {".SO3-": 79.9573, "HSO3-": 80.9652, ".SO4-": 95.9523, "HSO4-": 96.9601}

# The masses of singly charged ion losses to look for evidence of in the form of daught
er ions derived from multiply charged parent ions
ionLosses = {"IonLoss_.SO3-": 79.9573, "IonLoss_HSO3-": 80.9652, "IonLoss_.SO4-": 95.95
23, "IonLoss_HSO4-": 96.9601}

# The masses of neutral losses to look for evidence of
neutralLosses = {"NeutralLoss_SO3": 79.9568,"NeutralLoss_H2SO4":97.9674}

# The maximum ppm mass difference a signal can have from a theoretical value to be cons
idered a match to a reporter ion or daughter ion from an ion loss or neutral loss
```

```
                if abs(ppmError) < ppmErrorThreshold:
                    internalNeutralLossTransitions.append(n + ": " + transitionTag + "
(" + '{:.2f}'.format(ppmError) + " ppm discrepancy)")

        # We want to calculate the sum total of the signal intensities ascribed to inte
rnal neutral loss events
        # However, at this point, the intensity information we want to sum is buried in
the transitionTag strings in the internalNeutralLossTransitions list
        # so we have to do some hacky regular expression matching and string manipulati
on to get the numbers out and sum them up. This task could no doubt be handled more eff
iciently
        matches = re.findall(r'(?<=\[IntensitySum: )[0-9.]+', "|".join(internalNeutralL
ossTransitions))

        for m in matches:
            intensitySum = float(m.replace("[IntensitySum: ", "").replace("]", ""))
            totalInternalNeutralLossIntensity += intensitySum

        # Reset some variables for storing information about matching signals in the cu
rrent spectrum
        reporterIonIntensities = dict(zip(list(reporterIons.keys()), [0] * len(reporter
Ions))) #make a dict filled with zeros
        ionIntensityListStrings = [""] * len(reporterIons)

        # For convenience, pull the raw spectrum data into the variable 'spectrum'
        spectrum = spectra[spec]["spectrum"]

        # Loop through all the signal peaks in the spectrum
        for peak in spectrum:
            # And then loop through all the target signals in reporterIons to see if th
ey match the current peak
            for reporterName, reporterMZ in reporterIons.items():
                # If the reporterIons entry has a nonzero m/z value
                if reporterMZ != 0:
                    # Calculate the m/z difference in ppm between the current m/z peak
 and the expected m/z of the current reporter ion
                    ppmError = ppmErrorCalc(peak["mz"], reporterMZ);
                    # If the ppm error is below the threshold
                    if abs(ppmError) < ppmErrorThreshold:
                        # and the intensity of the peak is above the absolute threshold
for reporting as a reporter ion match
                        if peak["intensity"] > absoluteReporterIonIntensityThreshold:
                            # add the desired signal intensity (normalised or raw) to a
dictionary of values
                            reporterIonIntensities[reporterName] = peak[exportedSignal]
                else:
                    reporterIonIntensities[reporterName] = 0

        # Prepare string representations of all the reporter ion intensities
        ionIntensityList = list(reporterIonIntensities.values())
        ionIntensityListStrings = ['{:.6f}'.format(x) for x in ionIntensityList]

        # Extract the raw data file name from the entry title from the .mgf
        if 'File:""' in spectra[spec]["title"]:
            titleParts = spectra[spec]["title"].split(".")
            fileText = titleParts[0]
        else:
            matches=re.findall(r'\"(.+?)\"',spectra[spec]["title"])
            if matches[0] == None:
                fileText = ""
            else:
```

In [ ]:

```
# Spectrum Analyser
#
# Written by Dr Adam J. Carroll, RSB/RSC Joint Mass Spectrometry Facility, Australian N
ational University
#
# This is a simple python script for processing MS/MS spectra stored in Mascot Generic
 Format (.mgf) files to
# extract qualitative and quantitative information about evidence supporting fragmentat
ion mechanisms of interest
# and output the findings into a tab-delimited data matrix suitable for multivariate an
alysis.
#
# When executed, the script looks in the current working directory for .mgf files and p
rocesses them all, one-by-one,
# generating a report matrix for each .mgf file. The script parses the spectra in each
 .mgf and then looks for MS/MS
# signals with m/z values matching with user-settable reporter ions, ion losses and neu
tral losses.

# The customisable parameters may be set in the 'CUSTOMISABLE PARAMETERS' section just
 below (see explanations there).

#Import dependencies
import re, glob, os
from natsort import natsorted

#Set constants
PROTON_MASS = 1.00727647

### CUSTOMISABLE PARAMETERS ###

# If the .mgf files to process are in a different folder than the script, uncomment the
line below and set the full path to the folder containing the .mgf files
os.chdir('')
# The charge state to assign if no charge state is associated with the parent ion in th
e .mgf file
defaultCharge = -1

# The minimium signal intensity (in counts) required for a signal to be reported as a r
eporter ion signal
absoluteReporterIonIntensityThreshold = 0

# The minimum relative signal intensity (as % of base peak intensity) required for a si
gnal to be reported as a neutral loss signal
neutralLossTransitionRelativeIntensityThreshold = 5

# The annotations and m/z values of reporter ion signals to look for and report on
reporterIons = {".SO3-": 79.9573, "HSO3-": 80.9652, ".SO4-": 95.9523, "HSO4-": 96.9601}

# The masses of singly charged ion losses to look for evidence of in the form of daught
er ions derived from multiply charged parent ions
ionLosses = {"IonLoss_.SO3-": 79.9573, "IonLoss_HSO3-": 80.9652, "IonLoss_.SO4-": 95.95
23, "IonLoss_HSO4-": 96.9601}

# The masses of neutral losses to look for evidence of
neutralLosses = {"NeutralLoss_SO3": 79.9568,"NeutralLoss_H2SO4":97.9674}

# The maximum ppm mass difference a signal can have from a theoretical value to be cons
idered a match to a reporter ion or daughter ion from an ion loss or neutral loss
```

```
        for n in ionLosses:

            # If the precursor is multiply charged, there is a chance of observing a da
ughter ion formed by ion loss
            if abs(spectra[spec]["z"]) > 1:
                daughter_z = abs(spectra[spec]["z"]) - 1 #the charge of the daughter io
n will be 1 less than that of the parent
                daughter_m = spectra[spec]["m"] - ionLosses[n] #the mass of the daughte
r ion will be the mass of the parent ion minus the mass of the ion that is being lost
                daughter_mz = daughter_m / daughter_z #the m/z of the daughter ion from
the ion loss
                reporterIons[n] = daughter_mz # store the calculated m/z of the daughte
r ion in the reporterIons dictionary so we can look for it in the spectrum a bit later
            # If the precursor is not multiply charged, we won't bother looking for any
daughter ions from ion losses
            else:
                reporterIons[n] = 0

        # Reset some variables that will store information about signal annotations of
 the current spectrum for output later
        internalNeutralLossTransitions = []
        totalInternalNeutralLossIntensity = 0

        # For each of the neutral losses in the dictionary at the top of the script
        for n in neutralLosses:

            # First, we'll deal with neutral losses from the precursor ion, taking into
account the charge state of the precursor

            # Calculate the m/z shift expected from loss of this neutral mass, taking i
nto account the charge state of the precursor
            NL_delta_mz = neutralLosses[n] / abs(spectra[spec]["z"])

            # Calculate the actual m/z expected from the neutral loss by subtracting th
e delta m/z from the precursor m/z
            NL_abs_mz = spectra[spec]["mz"] - NL_delta_mz

            # Store this m/z in the theoretical reporter ions dictionary so we can look
for it in the spectrum later
            reporterIons[n] = NL_abs_mz

            # Next, we'll deal with neutral losses from all ions in the MS/MS spectrum
 by looking for pairs of signals
            # whose m/z difference is consistent with the mass of a target neutral loss
(internal neutral loss).
            # Note there are a couple of caveats to note here: if any parent signal is
 present in the MS/MS spectrum,
            # it will be treated the same as any other fragment and will therefore be i
ncluded with real 'internal' neutral
            # losses. Secondly, the code only matches neutral loss transitions occuring
between singly charged ions.

            # For each of the MS/MS fragment pairs we calculated delta m/z for earlie
r...
            for transitionTag, transition in spectra[spec]["deltaMasses"].items():
                # Calculate the ppm error between the observed m/z difference and the e
xpected m/z difference for the current neutral loss type
                ppmError = ppmErrorCalc(transition["deltaMass"], neutralLosses[n])
                # If the ppm error is below the threshold, add the details of the match
between the current mass transition
                # and the expected neutral loss transition to a list for reporting
```

```
                pepmassline = line.replace("PEPMASS=","")
                pepmassfields = pepmassline.split(" ")

                if len(pepmassfields) == 1:
                    precursorIntensity = 0
                else:
                    precursorIntensity = float(pepmassfields[1])

                mz = float(pepmassfields[0])

                spectra[specID]["mz"] = mz

                spectra[specID]["precursorIntensity"] = precursorIntensity
            elif "CHARGE=" in line:
                z = int(line.replace("CHARGE=","").replace("-", ""))
                if "-" in line:
                    spectra[specID]["z"] = -1 * z
                else:
                    spectra[specID]["z"] = z
            else:
                signalLineParts = line.split(" ")
                MS2mz = float(signalLineParts[0])
                MS2intensity = float(signalLineParts[1])
                spectra[specID]["spectrum"].append({'mz': MS2mz, 'intensity': MS2intens
ity})
                spectra[specID]["spectralData"]["mzs"].append(MS2mz)
                spectra[specID]["spectralData"]["intensities"].append(MS2intensity)
            if spectra[specID]["z"] == 0:
                spectra[specID]["z"] = defaultCharge

    # Once all the lines in the .mgf file have been parsed and spectral details stored
 in the spectra dictionary...
    for spec in spectra:

        # Use the normaliseSpectrum helper function to add an array of normalised inten
sities to the spectrum entry
        spectra[spec] = normaliseSpectrum(spectra[spec])

        # Use the computeDeltaMasses helper function to add a dictionary of qualifying
 mass transitions and their delta masses to the spectrum entry so we can try to match t
hem to neutral losses and ion losses
        spectra[spec] = computeDeltaMasses(spectra[spec])

    # Initialise variables required for generating the output report for this .mgf
    matrixReport = ""
    row_id = 0

    # Loop through the spectra held in the spectra dictionary
    for spec in spectra:

        # Increment row_id, which will be a unique id for each row of the output file
        row_id += 1

        # Before we search for

        # We have the precursor m/z already from parsing of the .mgf entry, so we add i
t to the reporterIons dictionary which will be used as the list of theoretical m/z valu
es to look for in the spectrum
        reporterIons["Precursor"] = spectra[spec]["mz"]

        # For each of the ion losses in the dictionary at the top of the script
```

```
def processMGF (mgfFile):

    global reporterIons

    # Derive an output report name from the input .mgf file and exportedSignal
    reporterIonMatrixReportFile = mgfFile.replace(".mgf", "_RepIonMatrix_5ppm" + export
edSignal + ".txt")

    # Open the .mgf file for reading
    f = open(mgfFile, 'r')

    # Initialise some variables
    specID = 0
    spectra = {}
    readingSpectrum = False

    # Loop through the lines in the .mgf file
    for line in f:

        # Strip whitespace and special characters from the right hand end of the line
        line = line.rstrip()

        # If the line contains the string "BEGIN IONS", we have encountered the beginni
ng of a new spectrum entry,
        # So we get ready to parse and store its contents in a new entry in a dictionar
y called 'spectra'
        if "BEGIN IONS" in line:
            readingSpectrum = True
            specID += 1
            spectra[specID] = {"title":"", "rt":0, "mz":0, "z":0}
            spectra[specID]["spectrum"] = []
            spectra[specID]["spectralData"] = {}
            spectra[specID]["spectralData"]["mzs"] = []
            spectra[specID]["spectralData"]["intensities"] = []
            spectra[specID]["z"] = 0
            continue

        # If the line contains the string "END IONS", we have encountered the end of th
e current spectrum entry
        # At this point, we calculate derived parameters, destroy the entry if it is in
valid (contains no signals) and exit parsing mode by setting readingSpectrum to false
        elif "END IONS" in line:
            spectra[specID]["mw"] = (spectra[specID]["mz"] * abs(spectra[specID]["z"]))
- (PROTON_MASS * spectra[specID]["z"])
            spectra[specID]["m"] = (spectra[specID]["mz"] * abs(spectra[specID]["z"]))
            if len(spectra[specID]["spectralData"]["mzs"]) == 0:
                del spectra[specID]
            readingSpectrum = False

        # If we are currently parsing a spectrum entry in the .mgf file because we are
 between BEGIN IONS and END IONS,
        # look for substrings in the line and parse out the important values accordingl
y
        if readingSpectrum:

            if "TITLE=" in line:
                spectra[specID]["title"] = line.replace("TITLE=","")

            elif "RTINSECONDS=" in line:
                spectra[specID]["rt"] = float(line.replace("RTINSECONDS=","")) / 60
            elif "PEPMASS=" in line:
```

```
nd mass2 exceed the neutralLossTransitionRelativeIntensityThreshold
            # and if they are not the same mass...
            if spectralData["normalisedIntensities"][mass1_i] > neutralLossTransitionRe
lativeIntensityThreshold and spectralData["normalisedIntensities"][mass2_i] > neutralLo
ssTransitionRelativeIntensityThreshold and mass1 != mass2:

                # Calculate the absolute difference between the two m/z values (i.e. th
e delta mass)
                deltaMass = abs(mass2-mass1)

                # Prepare string representations of the normalised intensities of mass
1, mass2 and their sum total, each to 2 decimal places
                intensity1 = '{:.2f}'.format(spectralData["normalisedIntensities"][mass
1_i])
                intensity2 = '{:.2f}'.format(spectralData["normalisedIntensities"][mass
2_i])
                totalTransitionIntensity = '{:.2f}'.format(spectralData["normalisedInte
nsities"][mass1_i] + spectralData["normalisedIntensities"][mass2_i])

                # Prepare string representations of mass1 and mass2, each to 5 decimal
 places
                mass1string = '{:.5f}'.format(mass1)
                mass2string = '{:.5f}'.format(mass2)

                # We need a unique string key for the transition represented by these t
wo signals (let's call them signals A and B) that is the same regardless of whether A a
nd B are held in mass1 and mass2, respectively, or vice versa
                # So we make a 2-element list of string representations (tags) of the t
wo signals (with mass value at the start of the string)...
                masstaglist = [mass1string + " (" + intensity1 + ")", mass2string + "
 (" + intensity2 + ")"]

                # ... and reverse natural sort it and join it into one string with a "-
>" symbol before adding a string representation of the sum total intensity of the pair
 to the string.
                # This will give a string 'Higher mass signal m/z (intensity) -> Lower
 mass signal m/z (intensity)[IntensitySum:totalTransitionIntensity]'
                # This will serve as a unique string key for the transition so we can m
ake a dictionary
                massTransitionTag = "->".join(list(reversed(natsorted(masstaglist)))) +
"[IntensitySum: " + totalTransitionIntensity + "]"

                # If the dictionary of transitions doesn't already include this transit
ion...
                if massTransitionTag not in massTransitionDict:
                    # create an empty dictionary under the mass transition's unique tag
key.
                    massTransitionDict[massTransitionTag] = {}

                # For the current transition (under the unique tag key), record its del
ta mass under the key "deltaMass"
                massTransitionDict[massTransitionTag]["deltaMass"] = deltaMass

    # Add the dictionary of mass transitions and their delta masses to the spectrum, un
der the key "deltaMasses"
    spectrum["deltaMasses"] = massTransitionDict

    return spectrum

# The main function that processes all the spectra in an MGF file and generates the out
put report
```

```
ppmErrorThreshold = 5

# Used to specify whether to include raw or normalised signal intensities in the output
report.
# For raw intensity, set to "intensity". For normalised intensity, set to "normalisedIn
tensity"
exportedSignal = "normalisedIntensity"
#exportedSignal = "intensity"
######

# A helper function to calculate the ppm error associated with an observed mass, relati
ve to an expected mass
def ppmErrorCalc (observed,expected):
    ppmError = ((observed - expected) / expected) * 1000000
    return ppmError

# A helper function to create a copy of a spectrum in which the signal intensities are
 all expressed as a percentage of the base peak intensity
def normaliseSpectrum (spectrum):

    spectralData = spectrum["spectralData"]
    maxIntensity = max(spectralData["intensities"])
    totalIntensity = sum(spectralData["intensities"])

    spectrum["spectralData"]["normalisedIntensities"] = []
    normalisedIntensities = []

    for i, val in enumerate(spectralData["intensities"]):
        spectrum["spectralData"]["normalisedIntensities"].append(100 * (spectralData["i
ntensities"][i]/maxIntensity))


    for i, val in enumerate(spectrum["spectrum"]):
        spectrum["spectrum"][i]["normalisedIntensity"] = 100 * (spectrum["spectrum"][i]
["intensity"] / maxIntensity)

    spectrum["maxIntensity"] = maxIntensity
    spectrum["totalIntensity"] = totalIntensity

    return spectrum

# Helper function to compute the m/z difference between every pair of m/z signals in a
 spectrum and store these in a dictionary
def computeDeltaMasses (spectrum):
    spectralData = spectrum["spectralData"]

    # Create a new dictionary to hold all the transitions for the current spectrum and
 their delta masses
    massTransitionDict = {}

    # Loop through all the m/z values in the spectrum, assigning them to the variable,
 mass1.
    # mass1_i is the index of m/z value in the "mzs" array of the spectrum
    for mass1_i, mass1 in enumerate(spectralData["mzs"]):

        # For every mass1, loop through all the m/z values in the spectrum again, assig
ning the value to mass2
        # mass2_i is the index of mass2 in the mzs array of the spectrum
        for mass2_i, mass2 in enumerate(spectralData["mzs"]):

            # If the normalised intensities (% of base peak instensity) of both mass1 a
```

In [ ]:

```
# Spectrum Analyser
#
# Written by Dr Adam J. Carroll, RSB/RSC Joint Mass Spectrometry Facility, Australian N
ational University
#
# This is a simple python script for processing MS/MS spectra stored in Mascot Generic
 Format (.mgf) files to
# extract qualitative and quantitative information about evidence supporting fragmentat
ion mechanisms of interest
# and output the findings into a tab-delimited data matrix suitable for multivariate an
alysis.
#
# When executed, the script looks in the current working directory for .mgf files and p
rocesses them all, one-by-one,
# generating a report matrix for each .mgf file. The script parses the spectra in each
 .mgf and then looks for MS/MS
# signals with m/z values matching with user-settable reporter ions, ion losses and neu
tral losses.

# The customisable parameters may be set in the 'CUSTOMISABLE PARAMETERS' section just
 below (see explanations there).

#Import dependencies
import re, glob, os
from natsort import natsorted

#Set constants
PROTON_MASS = 1.00727647

### CUSTOMISABLE PARAMETERS ###

# If the .mgf files to process are in a different folder than the script, uncomment the
line below and set the full path to the folder containing the .mgf files
os.chdir('')
# The charge state to assign if no charge state is associated with the parent ion in th
e .mgf file
defaultCharge = -1

# The minimum signal intensity (in counts) required for a signal to be reported as a r
eporter ion signal
absoluteReporterIonIntensityThreshold = 0

# The minimum relative signal intensity (as % of base peak intensity) required for a si
gnal to be reported as a neutral loss signal
neutralLossTransitionRelativeIntensityThreshold = 5

# The annotations and m/z values of reporter ion signals to look for and report on
reporterIons = {".SO3-": 79.9573, "HSO3-": 80.9652, ".SO4-": 95.9523, "HSO4-": 96.9601}

# The masses of singly charged ion losses to look for evidence of in the form of daught
er ions derived from multiply charged parent ions
ionLosses = {"IonLoss_.SO3-": 79.9573, "IonLoss_HSO3-": 80.9652, "IonLoss_.SO4-": 95.95
23, "IonLoss_HSO4-": 96.9601}

# The masses of neutral losses to look for evidence of
neutralLosses = {"NeutralLoss_SO3": 79.9568,"NeutralLoss_H2SO4":97.9674}

# The maximum ppm mass difference a signal can have from a theoretical value to be cons
idered a match to a reporter ion or daughter ion from an ion loss or neutral loss
```

```
                fileText = matches[0].replace('"', '').replace(',', '')

        # Add the tab-delimited row to a string containing the main body of the output
 report
        matrixReport += str(row_id) + "\t" + fileText + "|" + '{:.4f}'.format(spectra[s
pec]["rt"]) + "|" + '{:.6f}'.format(spectra[spec]["mz"]) + "|" + str(spectra[spec]["z"
]) + "\t" + fileText + "\t" + '{:.4f}'.format(spectra[spec]["rt"]) + "\t" + '{:.6f}'.fo
rmat(spectra[spec]["mw"]) + "\t" + '{:.6f}'.format(spectra[spec]["mz"]) + "\t" + str(sp
ectra[spec]["z"]) + "\t" + str(spectra[spec]["precursorIntensity"]) + "\t" + "\t".join(
ionIntensityListStrings)+ "\t" + '{:.1f}'.format(sum(ionIntensityList)-reporterIonInten
sities["Precursor"]) + "\t" + '{:.1f}'.format(sum(ionIntensityList)-reporterIonIntensit
ies["Precursor"]+ totalInternalNeutralLossIntensity) + "\t" + '{:.1f}'.format(spectra[s
pec]["totalIntensity"]) + "\t" + "; ".join(internalNeutralLossTransitions) + "\n"

    # Generate the tab-delimited header for the report, join it with the body and outpu
t to the file
    matrixReportHeader = "row_id\tAnalyteFullAnnotation\tDataFile\tRT [min]\tMolecular
 Weight\tm/z\tz\tPrecursor Intensity\t" + "\t".join([h + "_" + exportedSignal for h in
list(reporterIons.keys())])+ "\tTotal Reporter Intensity (without INL)\tTotal Reporter
 Intensity (with INL)\tTotal Intensity\tInternal Neutral Loss Transitions\n"
    matrixReport = matrixReportHeader + matrixReport
    reporterf = open(reporterIonMatrixReportFile, 'w')
    reporterf.write(matrixReport)
    reporterf.close()
    f.close()



# Find all the .mgf files in the current folder and process them all to generate a repo
rt for each one
for file in glob.glob("*.mgf"):
    print("Processing File: " + file)
    processMGF(file)

print("Done!")
```

Table Joiner Script

In [ ]:

```
#Table Joiner Script
#
# Written by Dr Adam J. Carroll, RSB/RSC Joint Mass Spectrometry Facility, Australian N
ational University
# This script was developed to map sulfate derived transition data onto aligneed MS1 da
ta on the basis of retention time and accurate precursor m/z

# load required libraries and set working directory
import sys
import os
import csv
import re

os.chdir()

#table headers row_id, RT [min], m/z
masterTable = "20210513_new_msdial_list.txt" #this is the Compounds table from CD after
opening in excel and saving as a tab-delimited .txt file
tableList = []

reportFile = "" # change this to whatever you want the output to be

primaryIndexHeader = "row_id"
idRegEx = r'.*'

join_ppm_tolerance =
join_RT_tolerance =  #Maximum RT Tolerance in sec

output_string = ""

def tab2dict(tableName, primaryIndexHeader, idRegEx):
    with open(tableName,encoding = "ISO-8859-1") as f:
        reader=csv.reader(f,delimiter='\t')
        headers = next(reader)
        #print(headers)
        numheaders = len(headers)
        columnindices = list(range(0, numheaders))

        header_indices = dict(zip(headers, columnindices))

        f.seek(0)
        next(f) # skip headings
        reader=csv.reader(f,delimiter='\t')

        tabdict = {}
        for line in reader:

            identifiers = re.findall(idRegEx, line[header_indices[primaryIndexHeader]],
re.IGNORECASE)

            for identifier in identifiers:
                tabdict[identifier.lower()] = {}
                tabdict[identifier.lower()]["dict"] = dict(zip(headers, line))
                tabdict[identifier.lower()]["list"] = line

    result = {}
    result["headers"] = headers
    result["tabdict"] = tabdict
    result["header_indices"] = header_indices
```

```
    result["tableName"] = tableName
    return result


masterDict = tab2dict(masterTable, primaryIndexHeader, idRegEx)


tableDicts = {}

joinedHeaders = masterDict["headers"]

for tableName in tableList:

    tableDicts[tableName] = tab2dict(tableName, "row_id", idRegEx)
    joinedHeaders.extend(tableDicts[tableName]["headers"])

joinedRows = []
joinedRows.append("\t".join(joinedHeaders))

for rowID, value in masterDict["tabdict"].items():
    masterDict["tabdict"][rowID]["matches"] = {}

    if rowID == "":
        continue

    print("Processing RowID: " + rowID)
    joinedRow = masterDict["tabdict"][rowID]["list"]

    rt1 = float(masterDict["tabdict"][rowID]["dict"]["RT [min]"]) * 60

    mw1 = float(masterDict["tabdict"][rowID]["dict"]["m/z"])

    for tableName in tableList:

        minRTDiff = join_RT_tolerance

        rowInit = False

        for table2rowID in tableDicts[tableName]["tabdict"]:

            #in case we don't find a match for this row, we need to initiate the top_ma
tch with blank cells
            if rowInit == False:
                masterDict["tabdict"][rowID]["top_match"] = [""]*len(tableDicts[tableNa
me]["tabdict"][table2rowID]["list"])
                rowInit = True

            rt2 = float(tableDicts[tableName]["tabdict"][table2rowID]["dict"]["RT [mi
n]"]) * 60
            mw2 = float(tableDicts[tableName]["tabdict"][table2rowID]["dict"]["m/z"])

            rtDiff = abs(rt2-rt1)

            mwDiff = abs(mw2-mw1)

            mwDiffppm = 1000000 * mwDiff / mw1;

            #if RT difference is less than join RT tolerance and MW ppm difference is l
ess that join ppm tolerance and RT difference is less than the current best (smallest)
 RT difference, update the top match for this row of the master table from this seconda
ry joining table
```

```
            if rtDiff < join_RT_tolerance and mwDiffppm < join_ppm_tolerance and rtDiff
< minRTDiff:

                masterDict["tabdict"][rowID]["top_match"] = tableDicts[tableName]["tabd
ict"][table2rowID]["list"]
                minRTDiff = rtDiff
                rowInit = True


        joinedRow.extend(masterDict["tabdict"][rowID]["top_match"])

    joinedRows.append("\t".join(joinedRow))

output_string = "\n".join(joinedRows)

text_file = open(reportFile, "w")
text_file.write(output_string)
text_file.close()
print("Done.")
```

S.8 Data Availability

Metabolomic data sets in '.RAW' format and in '.mzML' format have been uploaded to the MetaboLights online repository with the unique ID MTBLS3941.
